# Protein-confined imidazolium intermediates enable diverse biocatalytic C–C and C–N bond formations

**DOI:** 10.1038/s41929-026-01587-8

**Published:** 2026-07-31

**Authors:** Zachary Birch-Price, Amy E. Hutton, Fei Zhao, Mary Ortmayer, Veronika Stoianova, Christian Merten, Colin Levy, Richard Obexer, Anthony P. Green

**Affiliations:** 1 Manchester Institute of Biotechnology and Department of Chemistry, The University of Manchester, Manchester, UK; 2 Organische Chemie II, Fakultät für Chemie und Biochemie, Ruhr Universität Bochum, Bochum, Germany

**Keywords:** Biocatalysis, Enzyme mechanisms, X-ray crystallography

## Abstract

Developing enzymatic strategies to selectively construct C–C and C–heteroatom bonds is a major objective in modern biocatalysis. Here we combine genetic code reprogramming and laboratory evolution to establish a family of allylic transferase enzymes that can achieve a remarkable breadth of chemistry, enabling the generation of valuable motifs including γ‑butenolides, chiral amines and all-carbon quaternary centres. Our enzymes operate through the formation of electrophilic imidazolium intermediates that can be generated from reagents equipped with a *para*-nitrophenol leaving group to facilitate high-throughput evolution. These intermediates can be intercepted with diverse carbon and nitrogen nucleophiles to generate densely functionalized products featuring Cβ or Cγ stereocentres. Interestingly, structural analysis suggests that catalysis proceeds through an unanticipated ternary complex involving *para*-nitrophenol and the incoming substrate nucleophile. This study adds a family of C–C and C–N bond-forming enzymes to the biocatalytic repertoire and illustrates how artificial enzymes can achieve precise control over challenging chemical conversions.

**Figure:**
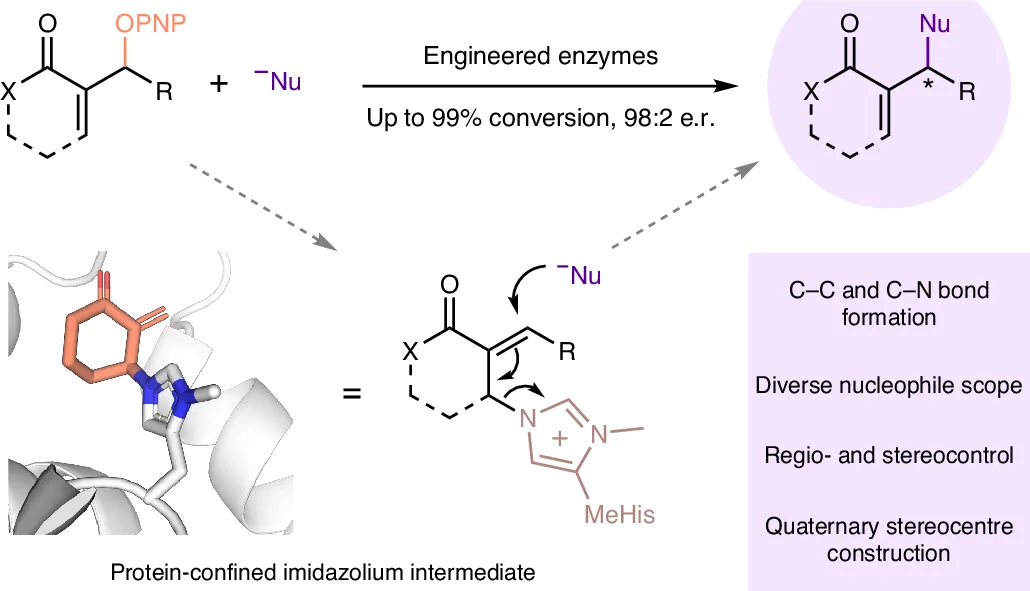

## Abstract (web summary)

Small catalytic nucleophiles can promote allylic transfer processes, but achieving regio- and stereocontrol is challenging. Here the authors develop a family of allylic transferase enzymes to mediate diverse C–C and C–N bond formations through the confinement of key intermediates within proteins.

## Main

Nucleophilic catalysis is a versatile mechanistic strategy used across chemistry and biology to accelerate a wide range of transformations. In biological systems, well-studied enzymes such as hydrolases^1^, acyltransferases^2,3^, phosphatases^4^, aldolases^5^ and glycosidases^6^ operate through the formation of transient covalent intermediates involving activated Ser, Cys, His, Asp or Lys nucleophiles. Beyond their fundamental interest, these enzyme classes are widely used as biocatalysts across the chemical industry for applications in, for example, pharmaceutical and commodity chemical manufacturing^7–10^, renewable feedstock utilization^11–13^ and bioremediation^14–16^. The scope of enzymatic nucleophilic catalysis has been further expanded through directed evolution and/or computational design, including the development of enzymes for non-biological transformations^17–27^.

**Fig. 1:**
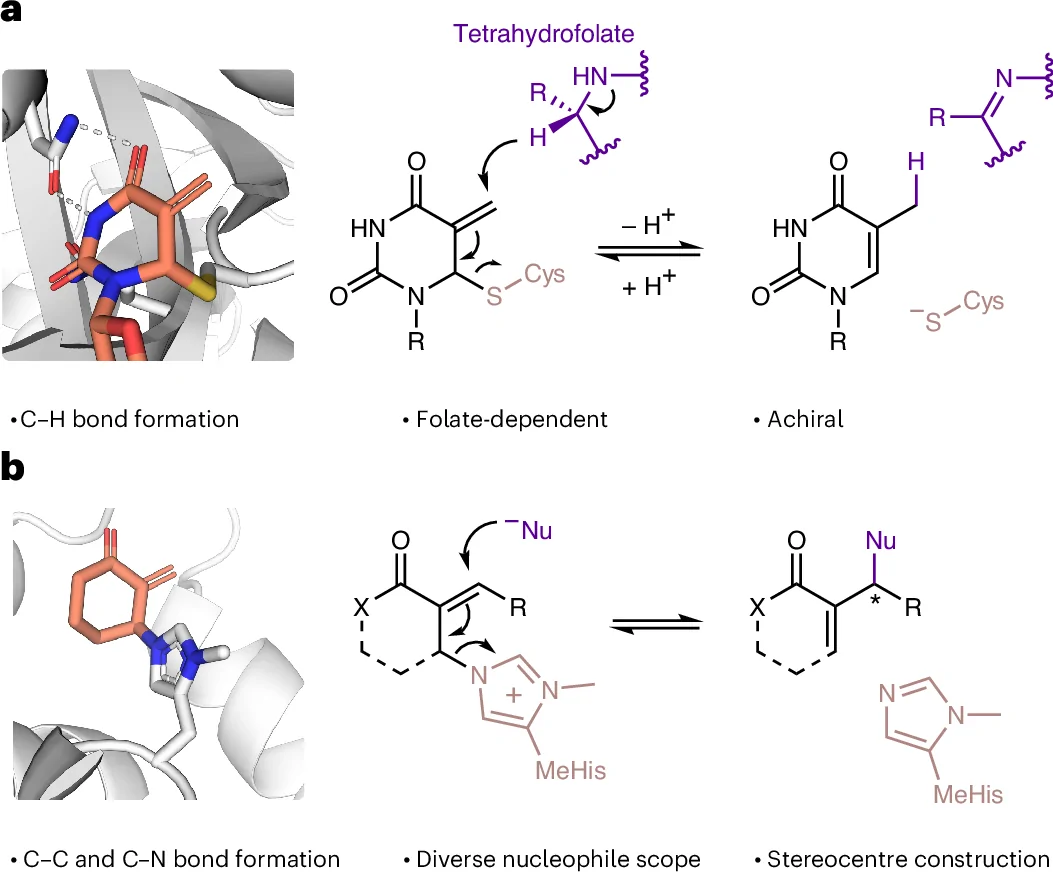
Reactivity of covalent electrophilic intermediates in natural and engineered enzymes. **a**, TS uses a Cys nucleophile to activate uracil and mediate addition of a hydride from tetrahydrofolate to the exocyclic methylene group of the electrophilic intermediate to generate thymidine. The active site representation is based on Protein Data Bank (PDB) entry 1KZI (ref. ^61^). **b**, Our approach involves the generation of analogous covalent intermediates that can be intercepted with a variety of nucleophiles to generate structurally diverse products. The active site representation is based on PBD entry 9R2D (vide infra).

In addition to these well-established enzyme families, other less-studied enzymes are known that operate through nucleophilic catalysis, which in principle could inspire catalytic approaches that unlock areas of chemical space not currently accessible to biocatalysis. One remarkable example is the enzyme thymidylate synthase (TS), which uses a catalytic Cys to activate the α,β-unsaturated carbonyl moiety of uracil, leading to a potent electrophilic intermediate featuring an exocyclic methylene unit^28,29^. In the native enzyme, this intermediate is intercepted by a hydride nucleophile supplied by tetrahydrofolate, leading to the methylated nucleobase thymidine (Fig. 1a). In principle, if we were able to generate analogous electrophilic intermediates in readily engineerable protein scaffolds, it should be possible to intercept these species with a variety of carbon-based nucleophiles to unlock valuable C–C bond-forming chemistries within enzyme active sites (Fig. 1b). While precedent for similar transformations can be found in the synthetic chemistry literature^30–33^, there are restrictions on the range of accessible electrophile and nucleophile coupling partners. For example, achieving regiocontrolled transformations can often be problematic due to the competing reactivity of the catalytic nucleophile and nucleophilic substrate, leading to mixtures of Cβ- and Cβ′-substituted products^34–37^. In principle, these limitations could be alleviated through enzyme catalysis, where potent catalytic nucleophiles can be contained within hydrophobic protein pockets adjacent to well-defined substrate binding sites. Within a suitably engineered active site, it should be possible to accurately control the order of catalytic versus substrate nucleophile addition at distinct electrophilic sites in the substrate (Cβ′) and intermediate (Cβ) states (Supplementary Fig. 1).

In this study, we capitalized on genetic code reprogramming methods^38^ to generate electrophilic imidazolium intermediates, akin to those found in TS, within an evolvable protein host. We subsequently found that this protein can be engineered to operate with diverse nucleophilic coupling partners to afford a family of enzymes for stereocontrolled C–C and C–N bond constructions.

## Results

### Engineering enantioselective allylic transferases

**Fig. 2:**
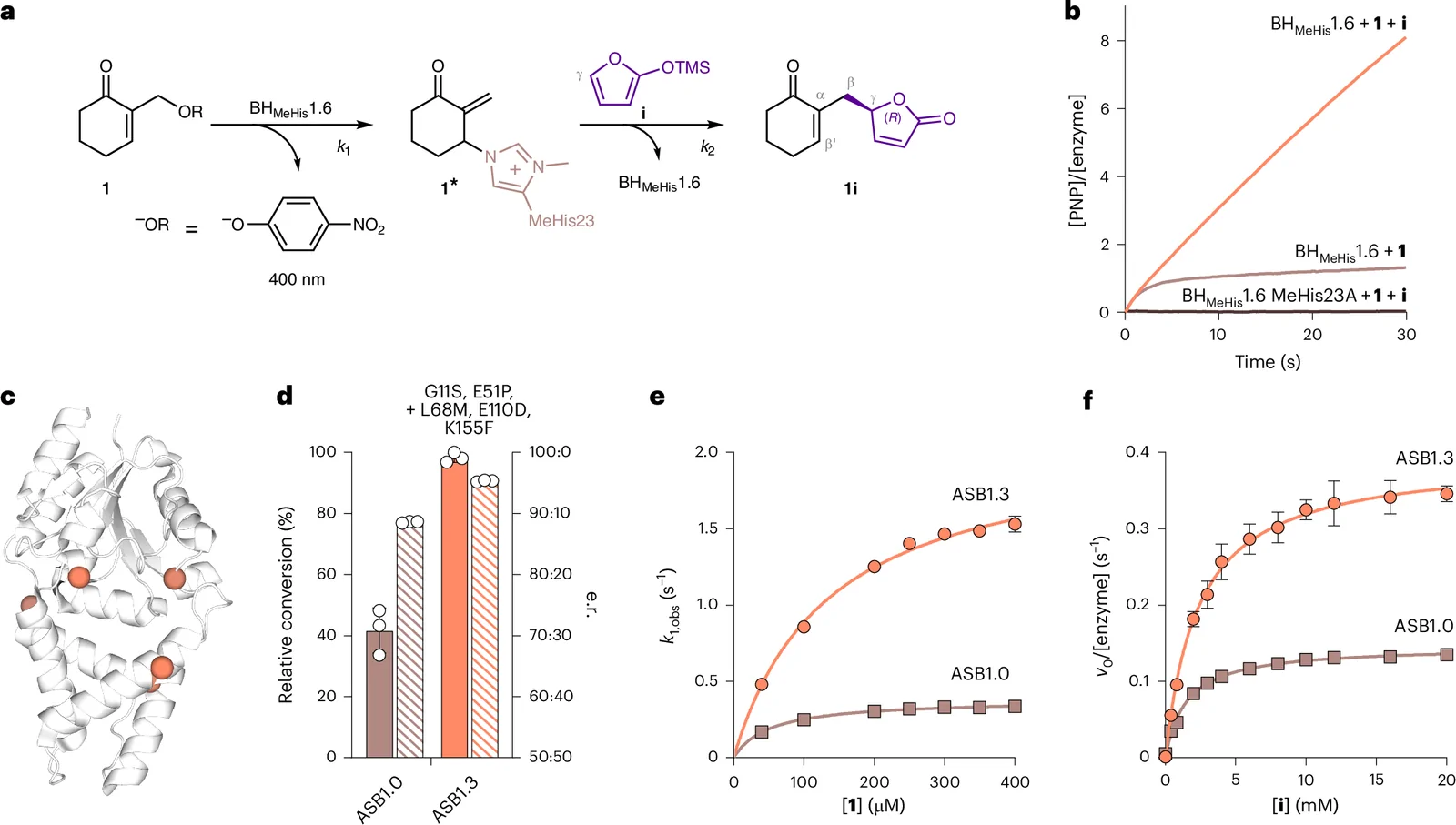
Directed evolution of an allylic transferase for γ-butenolide synthesis. **a**, Proposed allylic transferase mechanism for the formation of product **1i**. TMS, trimethylsilyl. **b**, Time courses for the reaction of substrate **1** with BH_MeHis_1.6 in the presence and absence of nucleophile **i**. The mutation of MeHis23 to Ala abolishes activity. Reactions were performed using 400 µM **1**, 8 mM **i** and 20 µM enzyme in phosphate-buffered saline (PBS; pH 7.4) with 5% (v/v) MeCN. PNP release was monitored by the absorbance at 400 nm. **c**, Mutational map of ASB1.3, showing the positions mutated during evolution (the spheres represent Cα atoms; Supplementary Table 1) mapped onto the structure of BH_MeHis_1.8 (five mutations from starting template BH_MeHis_1.6; PDB ID 8BP0 (ref. ^25^)). **d**, Bar chart showing the relative conversions (solid bars) and e.r. values (hatched bars) for the transformation of **1** and **i** to (*R*)-**1i** catalysed by ASB1.0 and ASB1.3. Conversions were determined using 400 µM **1**, 800 µM **i** and 0.4 µM enzyme in PBS (pH 7.4) with 5% (v/v) MeCN for 30 min. The e.r. values were determined under identical reaction conditions but using 2 µM enzyme. Data are presented as mean values of measurements performed in triplicate; error bars indicate the standard deviation. The absolute configuration of product **1i** was determined by vibrational circular dichroism (VCD) analysis^62^ (Supplementary Fig. 3a). **e**, Plot showing the rate constants for the formation of intermediate 1* (*k*_1,obs_) at varying concentrations of substrate **1** for ASB1.0 and ASB1.3. Reactions were performed using 20 µM enzyme in PBS (pH 7.4) with 5% (v/v) MeCN. Data are presented as mean values of measurements performed in triplicate; error bars indicate the standard deviation. **f**, Michaelis–Menten plots for the reaction of **1** and **i** catalysed by ASB1.0 and ASB1.3. Reactions were performed using 400 µM **1**, 1 µM enzyme and varying concentrations of nucleophile **i** in PBS (pH 7.4) with 5% (v/v) MeCN. Data are presented as mean values of measurements performed in triplicate; error bars indicate the standard deviation.

To identify a suitable starting template for engineering allylic transferases, we considered a family of Morita–Baylis–Hillman (MBH) enzymes recently engineered by our group to promote the coupling of cyclohexenone with aromatic aldehydes^25,39^. These enzymes harbour potent His or *N*δ-methylhistidine (MeHis) catalytic nucleophiles that could be repurposed to promote the target chemistry. To unlock allylic transfer activity, we initially designed a cyclohexenone-based electrophilic reagent **1** equipped with a *para*-nitrophenol (PNP) leaving group (Fig. 2a). Reaction of **1** with an appropriate catalytic nucleophile should generate a covalent intermediate featuring an electrophilic exocyclic methylene unit (1*) and release the PNP chromophore for facile spectrophotometric reaction monitoring. Subsequent reaction of the electrophilic intermediate with an appropriate substrate nucleophile should result in product formation and regeneration of the catalytic nucleophile for further turnovers.

**Extended Data Fig. 1:**
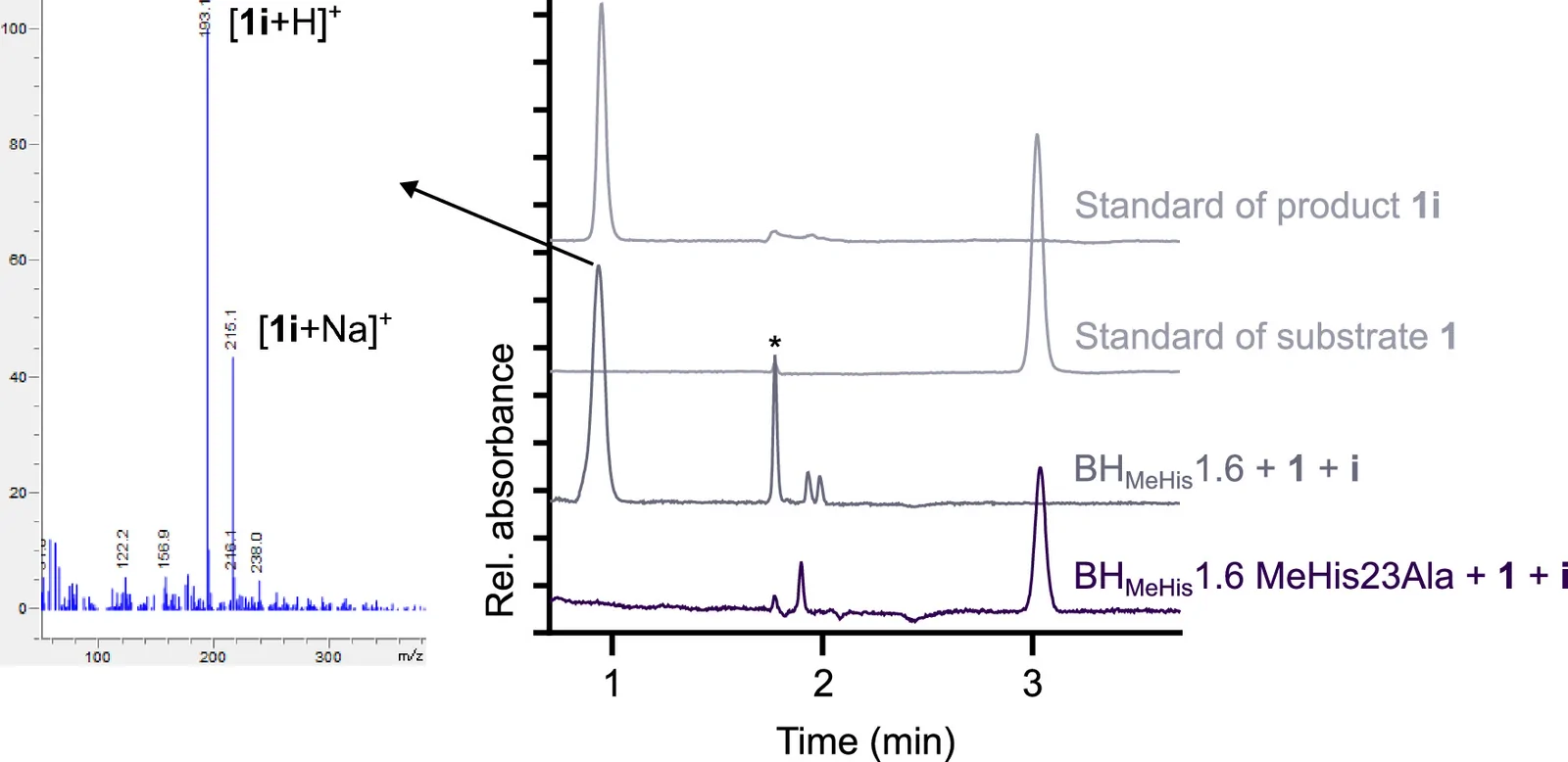
Transformation of 1 and i to 1i catalysed by BH_MeHis_1.6. LC-MS chromatograms (230 nm absorbance) of substrate **1** and product **1i**, and biotransformations of **1** and **i** with BH_MeHis_1.6 or BH_MeHis_1.6 MeHis23Ala. Mass spectrum of **1i** peak in BH_MeHis_1.6 trace shown on left. Reactions were performed using 50 µM **1**, 500 µM **i** and 5 µM enzyme in PBS pH 7.4 with 5% MeCN for 1 hour. * PNP peak.

Following evaluation of a broad selection of in-house MBH enzymes by monitoring absorbance changes at 400 nm, we identified one of our MeHis-containing variants, BH_MeHis_1.6, as a promising candidate for further development. Addition of an excess of **1** to BH_MeHis_1.6 resulted in a fast ‘burst phase’ arising from the release of 1 equiv. PNP relative to the BH_MeHis_1.6 concentration (Fig. 2b). This burst phase was abolished upon mutation of MeHis23 to Ala. These observations are consistent with the formation of a covalent enzyme intermediate involving MeHis23 that is stable in the absence of exogenous coupling partners. Subsequent addition of oxyfuran **i** as a nucleophilic coupling partner, which was expected to give rise to chiral butenolide motifs commonly found in bioactive natural products^40^, resulted in the production of multiple equivalents of PNP. Liquid chromatography–mass spectrometry (LC–MS) analysis of biotransformations using **1** and **i** as coupling partners revealed the formation of a single product with *m*/*z* = 193, consistent with the target product **1i** (Extended Data Fig. 1). This species was not observed in biotransformations performed with the MeHis23Ala variant of BH_MeHis_1.6, consistent with an enzymatic mechanism involving nucleophilic catalysis. To confirm the identity of **1i**, we performed a preparative-scale biotransformation and characterized the isolated product by NMR analysis (Methods). Notably, we were unable to produce a racemic standard of **1i** using small-molecule nucleophilic catalysts, underscoring the potential of engineered allylic transferases to mediate transformations that are challenging with existing methodologies.

**Extended Data Fig. 2:**
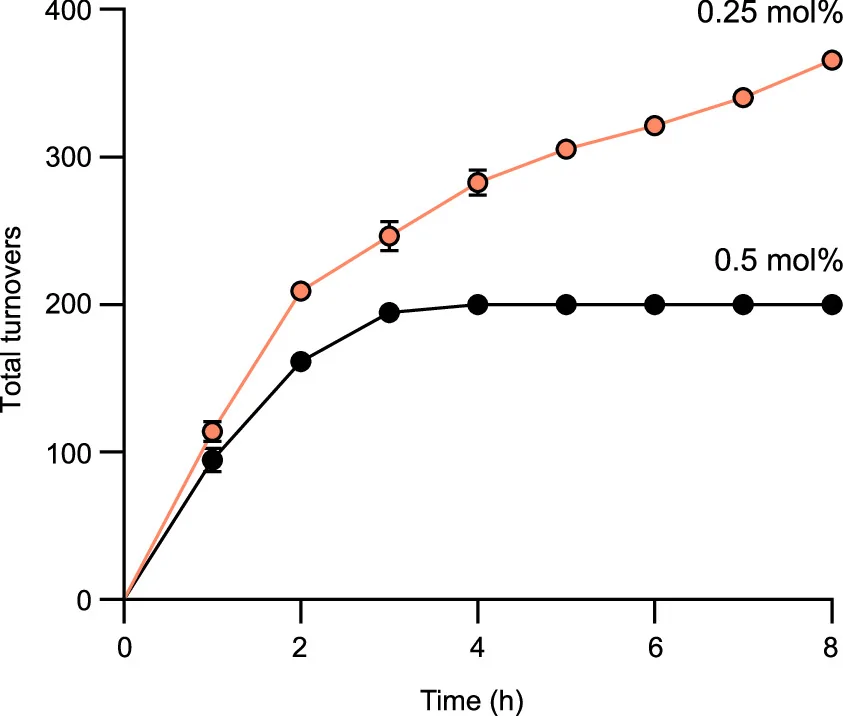
Total turnover numbers achieved by ASB1.3. Reactions were performed using **1** (200 µM) and **i** (400 µM) in PBS pH 7.4 with 5% MeCN, using either 0.5 mol% (black) or 0.25 mol% (orange) of ASB1.3. Data are presented as mean values of triplicate measurements, with error bars indicating standard deviation.

To improve enzyme activity and selectivity, BH_MeHis_1.6 (subsequently referred to as ASB1.0) was subjected to three rounds of laboratory evolution, facilitated by our recently engineered *ISO4-G1* pyrrolysyl tRNA synthetase (PylRS) that allows genetic incorporation of MeHis with high efficiency^41^. In each round, 20–24 residues were individually randomized using NNK (N = A, G, C or T and K = G or T) degenerate codons. Individual library members were arrayed in 96-well plates and evaluated as clarified cell lysates using the aforementioned spectrophotometric assay. The most active (~1%) clones from each round were further evaluated as purified proteins for improved activity and selectivity using HPLC analysis. Beneficial mutations identified in each round were subsequently combined by DNA shuffling. Following evaluation of >4,000 clones, an ASB1.3 variant emerged containing five mutations clustered around the active site (Fig. 2c) that displays a 2.4-fold improvement in reaction conversion under the assay conditions used for evolution (Fig. 2d). Pleasingly, evolution also led to improvements in enantioselectivity, with ASB1.3 affording (*R*)-**1i** with a 96:4 enantiomeric ratio (e.r.). Kinetic analysis of the initial and final variants revealed substantial increases in the formation rate of intermediate 1* (*k*_1,obs_ = 0.34 and 1.53 s^−1^ under saturating concentrations of **1** for ASB1.0 and ASB1.3, respectively; Fig. 2e) and overall catalytic turnover (*k*_cat_ = 0.13 and 0.35 s^−1^, respectively; Fig. 2f). ASB1.3 was also able to perform over 350 turnovers (Extended Data Fig. 2). To demonstrate its synthetic utility, we performed a preparative-scale biotransformation using 0.5 mol% ASB1.3 to afford optically enriched **1i** (15.8 mg) with 98% conversion and 69% isolated yield (Supplementary Fig. 2).

**Fig. 3:**
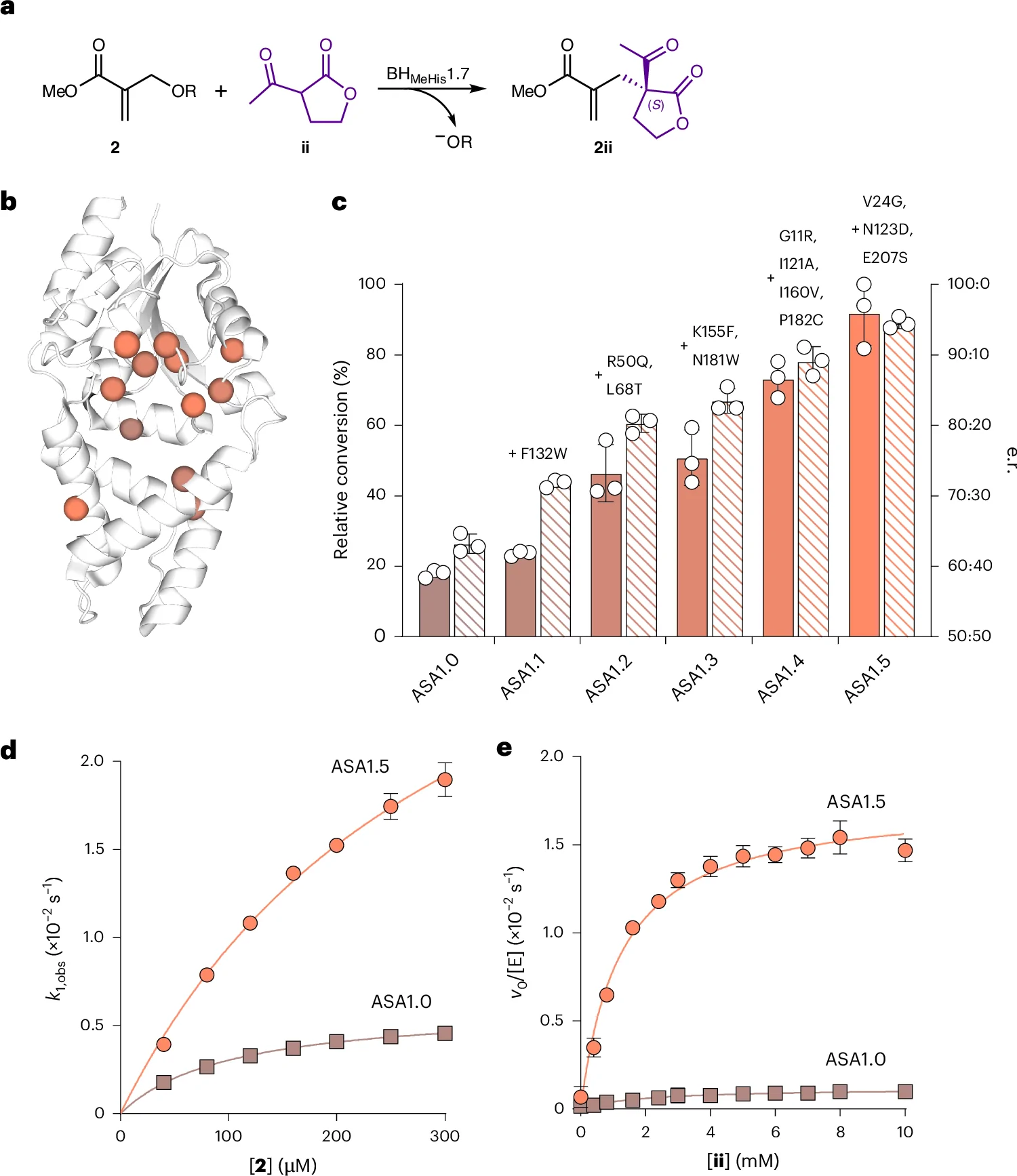
Directed evolution of an allylic transferase for construction of all-carbon quaternary centres. **a**, Reaction scheme for the conversion of **2** and **ii** to **2ii**. R = 4-NO_2_-Ph. **b**, Mutational map of ASA1.5, showing the positions mutated during evolution (the spheres represent Cα atoms; Supplementary Table 2) mapped onto the structure of BH_MeHis_1.8 (two mutations from starting template BH_MeHis_1.7; PDB ID 8BP0 (ref. ^25^)). **c**, Bar chart showing the relative conversions (solid bars) and e.r. values (hatched bars) for the transformation of **2** and **ii** to (*S*)-**2ii** catalysed by selected ASA variants along the evolutionary trajectory. Conversions were determined using 400 µM **2**, 800 µM **ii** and 2 µM enzyme in PBS (pH 7.4) with 5% (v/v) MeCN for 40 min. The e.r. values were determined under identical reaction conditions but using 20 µM enzyme for 3 h. Data are presented as mean values of measurements performed in triplicate; error bars indicate the standard deviation. The absolute configuration of product **2ii** was determined by VCD analysis^62^ (Supplementary Fig. 3b). **d**, Plot showing the rate constants for intermediate formation (*k*_1,obs_) at varying concentrations of substrate **2** for ASA1.0 and ASA1.5. Reactions were performed using 20 µM enzyme in PBS (pH 7.4) with 5% (v/v) MeCN. Data are presented as mean values of measurements performed in triplicate; error bars indicate the standard deviation. PNP release was monitored by the absorbance at 400 nm. **e**, Michaelis–Menten plots for the reaction of **2** and **ii** catalysed by ASA1.0 and ASA1.5. Reactions were performed using 300 µM **2** (solubility limit), 2 µM enzyme and varying concentrations of **ii** in PBS (pH 7.4) with 5% (v/v) MeCN. Data are presented as mean values of measurements performed in triplicate; error bars indicate the standard deviation.

**Extended Data Fig. 3:**
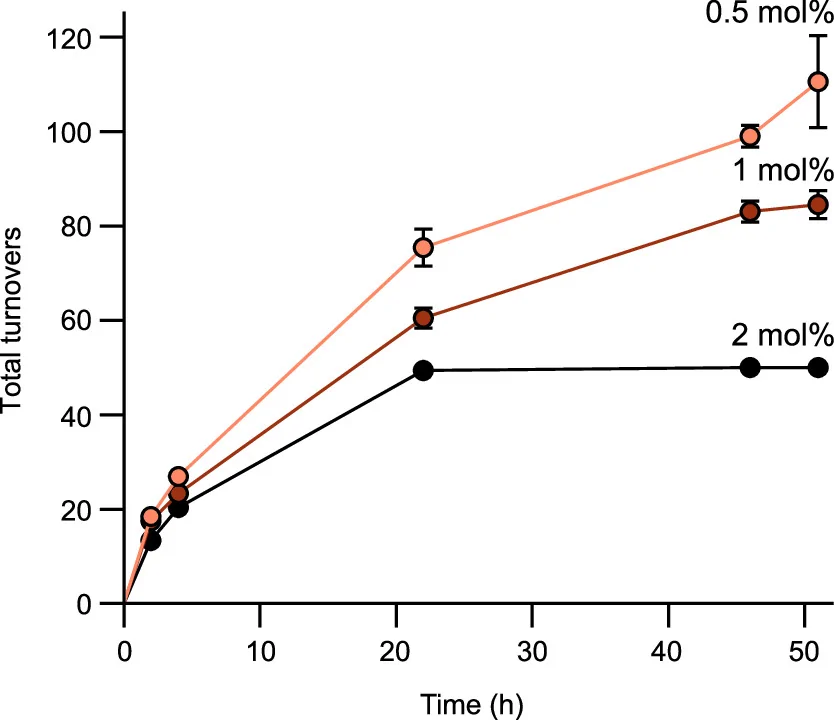
Total turnover numbers achieved by ASA1.5. Reactions were performed using **2** (100 µM) and **ii** (200 µM) in PBS pH 7.4 with 5% MeCN, using either 2 mol% (black), 1 mol% (brown) or 0.5 mol% (orange) of ASA1.5. Data are presented as mean values of triplicate measurements, with error bars indicating standard deviation.

To demonstrate the versatility of our allylic transferase biochemistry, we next explored the reaction of acrylate **2** as an electrophilic coupling partner with lactone **ii** to generate densely functionalized product **2ii** containing an all-carbon quaternary stereocentre (Fig. 3a). Compared with ketone-based electrophiles such as **1**, acrylates are more challenging to activate due to their reduced electrophilicity, and so far we have been unable to achieve biocatalytic MBH reactions with this class of substrates. Nevertheless, following evaluation of our in-house MBHases, we identified a BH_MeHis_1.7 variant (subsequently referred to as ASA1.0) with modest activity and selectivity towards the target transformation. To enhance enzyme efficiency, we performed directed evolution using workflows similar to those described above. Following evaluation of >7,000 clones, an ASA1.5 variant emerged containing 12 mutations (Fig. 3b). Compared with the parent template, this variant displays a 5.1-fold improvement in reaction conversion (18% versus 92%) and a substantial increase in selectivity for the formation of (*S*)-**2ii** (63:37 e.r. with ASA1.0 versus 95:5 e.r. with ASA1.5; Fig. 3c). These improvements arise from a 4.2-fold increase in *k*_1,obs_ (at the solubility limit of **2**; Fig. 3d) and a substantial 15-fold increase in catalytic turnover (*k*_obs_^app^ = 0.10 × 10^−2^ and 1.47 × 10^−2^ s^−1^ for ASA1.0 and ASA1.5, respectively; Fig. 3e). ASA1.5 can perform over 100 turnovers (Extended Data Fig. 3) and is suitable for preparative-scale biotransformations, with 19.7 mg **2ii** produced in 98% conversion (68% isolated yield) using only 1.25 mol% of ASA1.5 (Supplementary Fig. 4).

**Fig. 4:**
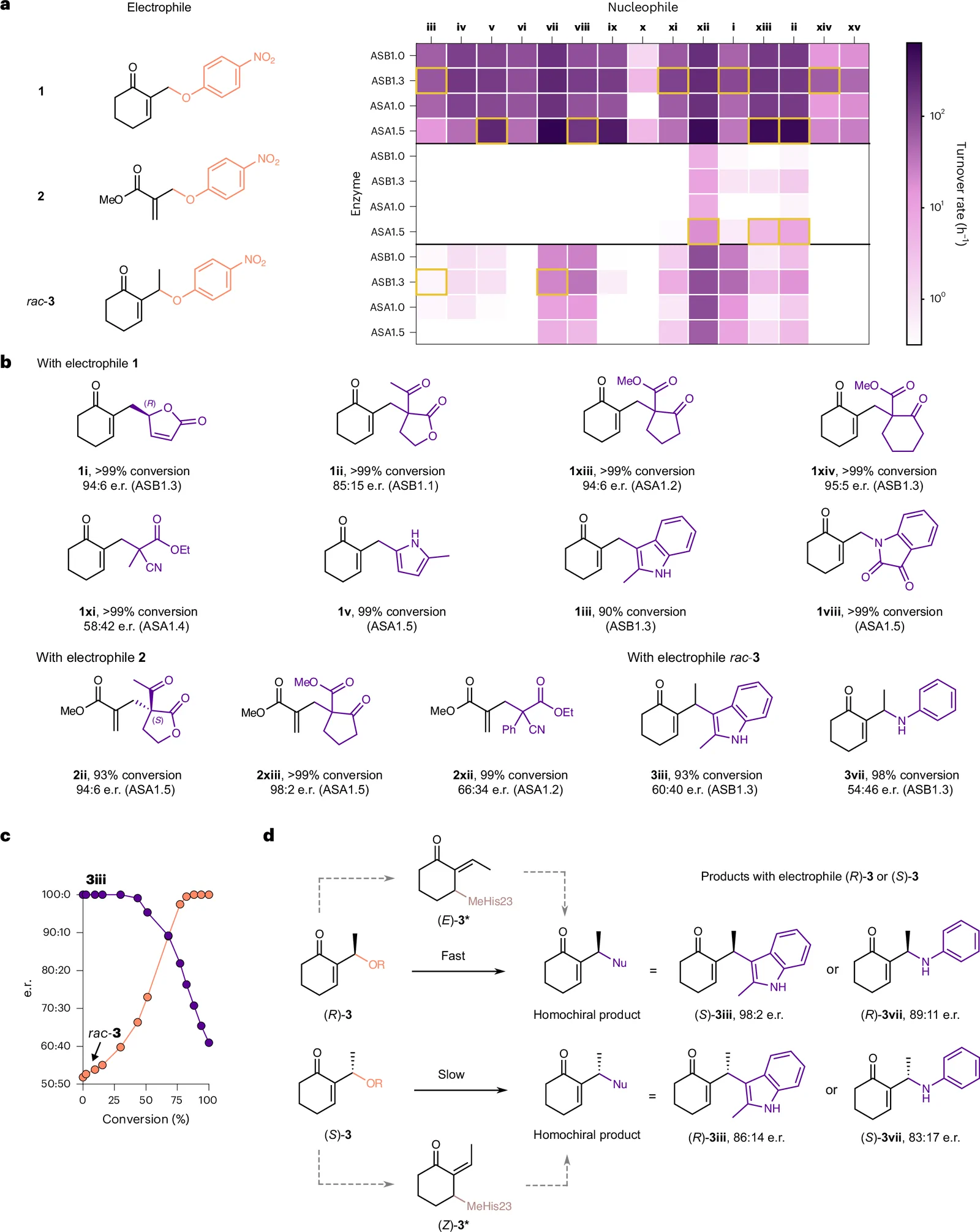
Substrate scope of allylic transferases. **a**, Heatmap showing initial turnover rates for different combinations of electrophile, nucleophile and enzyme. Yellow outlines indicate transformations selected for further characterization. Reactions were performed using 100 µM electrophile, 200 µM nucleophile and 10 µM enzyme in PBS (pH 7.4) with 5% (v/v) MeCN. PNP release was monitored by the absorbance at 400 nm. Data are presented as mean values of measurements performed in triplicate. The structures of nucleophiles **i**–**xv** are shown in Supplementary Fig. 5. **b**, Selected biotransformations catalysed by evolved allylic transferases. Conversions and e.r. values are presented as mean values of measurements performed in triplicate. The conditions for each biotransformation are given in Supplementary Table 3. **c**, Plot showing the conversion and e.r. of electrophile **3** and product **3iii** during ASB1.3-catalysed coupling of *rac*-**3** with nucleophile **iii**. The reaction was performed using 400 µM *rac*-**3**, 1,600 µM **iii** and 20 µM ASB1.3 in PBS (pH 7.4) with 5% (v/v) MeCN. Timepoints were taken at regular intervals over 4 h. **d**, Using optically pure (*R*)-**3** or (*S*)-**3** as substrate gives rise to divergent stereochemical outcomes. This transfer of stereochemical information likely proceeds via the selective formation of either the (*E*) or (*Z*) isomer of intermediate 3*. Reaction conditions are given in Supplementary Table 3. The absolute configuration of (*R*)-**3** was determined by VCD analysis^62^ (Supplementary Fig. 3c). The absolute configurations of **3iii** and **3vii** were assigned based on structural modelling (Supplementary Fig. 6). Source data

To explore the scope of our engineered allylic transferases, we capitalized on our spectrophotometric assay to rapidly profile their activity towards diverse nucleophile coupling partners **i**–**xv** (Fig. 4a and Supplementary Fig. 5). We also expanded our panel of electrophiles to include *rac*-**3**, which was expected to generate products with a Cβ stereocentre to complement the Cγ stereocentres generated in the reactions with prochiral nucleophiles. These assays showed that our engineered biocatalysts can couple electrophiles **1** and *rac*-**3** with diverse nucleophiles covering a broad range of chemical space, including substituted furans, indoles, pyrroles, cyanoesters, diketones and ketoesters as putative carbon nucleophiles, as well as anilines and isatins as nitrogen nucleophiles. A varied, but somewhat more restrictive nucleophile scope was observed using electrophile **2** as a coupling partner, likely reflecting the reduced reactivity of the imidazolium intermediates derived from acrylate coupling partners. For the vast majority of electrophile and nucleophile coupling partners, directed evolution has afforded enzymes with increased activity, and in some cases (**2** + **xiii** and **3** + **ix**) has unlocked activity that was not observable with the parent enzymes.

**Extended Data Fig. 4:**
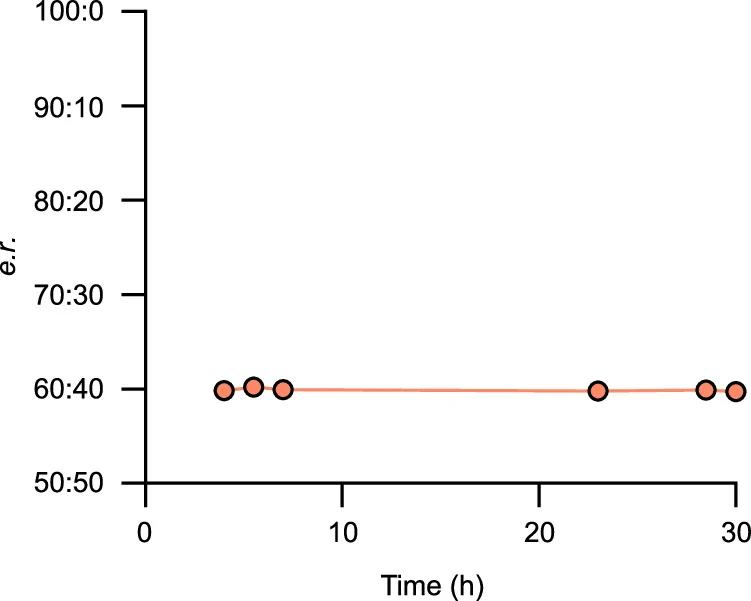
Configurational stability of product 3iii under reaction conditions. Plot of *e.r*. values of product **3iii** *vs*. time. Reaction was performed using 400 µM *rac*-**3**, 1600 µM **iii** and 20 µM ASB1.3 in PBS pH 7.4 with 5% MeCN. Reaction conversion was >99.9% after 4 hours (first timepoint) as verified by UPLC analysis. Data are presented as mean values of triplicate measurements, with error bars indicating standard deviation.

**Extended Data Fig. 5:**
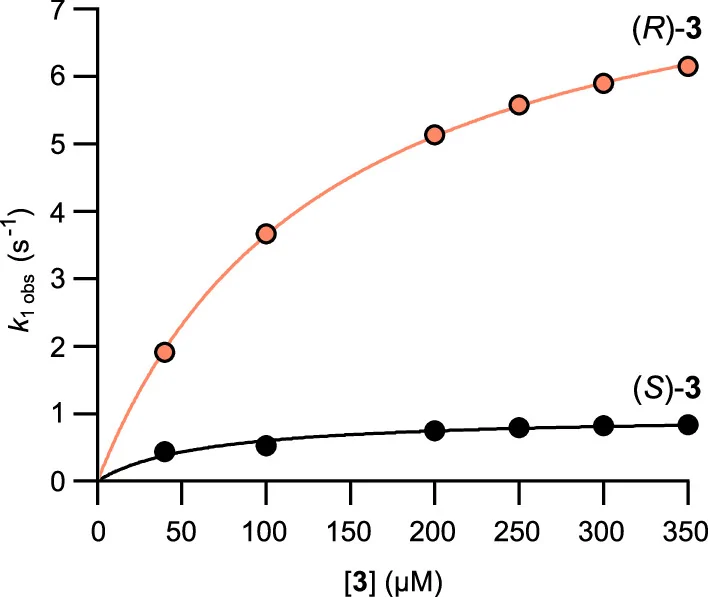
Substrate preference of ASB1.3 for (*R*)-3 over (*S*)-3. Plot showing the rate of formation of intermediate 3* (*k*_1 obs_) at varying concentrations of substrate (*R*)-**3** (orange) and (*S*)-**3** (black) for ASB1.3. Reactions were performed using 20 µM enzyme in PBS pH 7.4 with 5% v/v MeCN. PNP release was monitored by absorbance at 400 nm. Data are presented as mean values of triplicate measurements, with error bars indicating standard deviation.

Based on this screening data, we selected a range of biotransformations (outlined in yellow in Fig. 4a) for more detailed analysis to confirm the identity of products generated and quantify reaction conversions and selectivities (Fig. 4b). This analysis confirmed that our allylic transferases can mediate diverse C–C and C–N bond formations to generate a wide range of products with high conversions and appreciable levels of stereocontrol. In all cases a single reaction product was formed, with no observable by-products other than PNP. During reaction optimization for transformations involving *rac*-**3**, we observed that product e.r. varied as a function of reaction conversion, with high selectivities observed at low conversions that were then eroded as conversion increased (Fig. 4c). This trend coincided with an increasing e.r. of the remaining substrate **3** during the reaction. We also noted that the product was configurationally stable under the reaction conditions (Extended Data Fig. 4). These observations are consistent with a model in which (1) the stereochemistry of the starting material dictates the stereochemistry of the product formed and (2) the enantiomers of the starting material react at different rates. To test this hypothesis, we performed biotransformations with individual enantiomers of **3** in combination with coupling partners **iii** and **vii** (Fig. 4d). In both cases, despite progressing through planar reaction intermediates, we observed that the reactions proceeded in a stereospecific fashion to give divergent stereochemical outcomes depending on the stereochemistry of the starting material. This transfer of stereochemical information is likely mediated by the formation of either the (*E*) or (*Z*) isomer of covalent intermediate 3*, which in turn is dictated by the stereochemistry of reagent **3**. We also observed that ASB1.3 has an innate preference for (*R*)-**3** over (*S*)-**3**, with a 7.4-fold difference in the rate of formation of intermediate 3* observed (*k*_1,obs_ = 6.15 and 0.83 s^−1^, respectively, at the solubility limit of **3**; Extended Data Fig. 5).

### ASB1.3 structure and mechanism

**Fig. 5:**
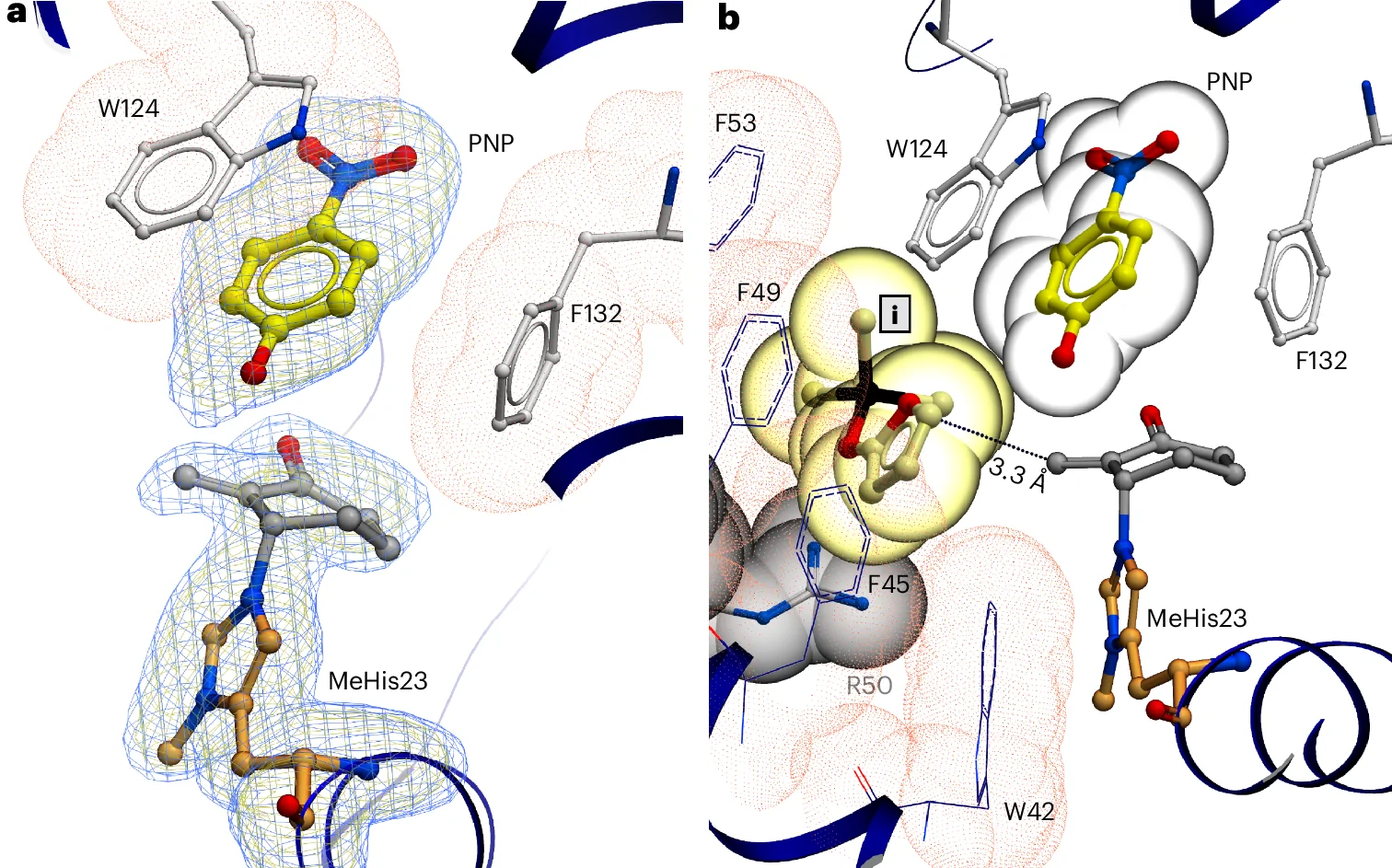
Structural analysis of ASB1.3 with a covalent imidazolium intermediate. **a**, Active-site view of the 1*–ASB1.3 complex, showing the covalent linkage between MeHis23 (orange carbon atoms) and intermediate 1* (grey carbon atoms). PNP (yellow carbon atoms) is bound in an aromatic pocket formed by Trp124 and Phe132 (light-grey carbon atoms). The 2*F*_o_ − *F*_c_ (*F*_o_, observed structure factors; *F*_c_, calculated structure factors) electron density map is shown as a mesh contoured at 1.0*σ* (blue) and 2.0*σ* (yellow). **b**, Docking of nucleophile **i** (light-yellow carbon atoms and Corey–Pauling–Koltun (CPK) spheres) in the X-ray structure of the 1*–ASB1.3 complex shows that **i** occupies a pocket formed by Trp42, Phe45, Phe49, Arg50 and Phe53 and packs adjacent to the bound PNP (yellow carbon atoms and white CPK spheres).

**Extended Data Fig. 6:**
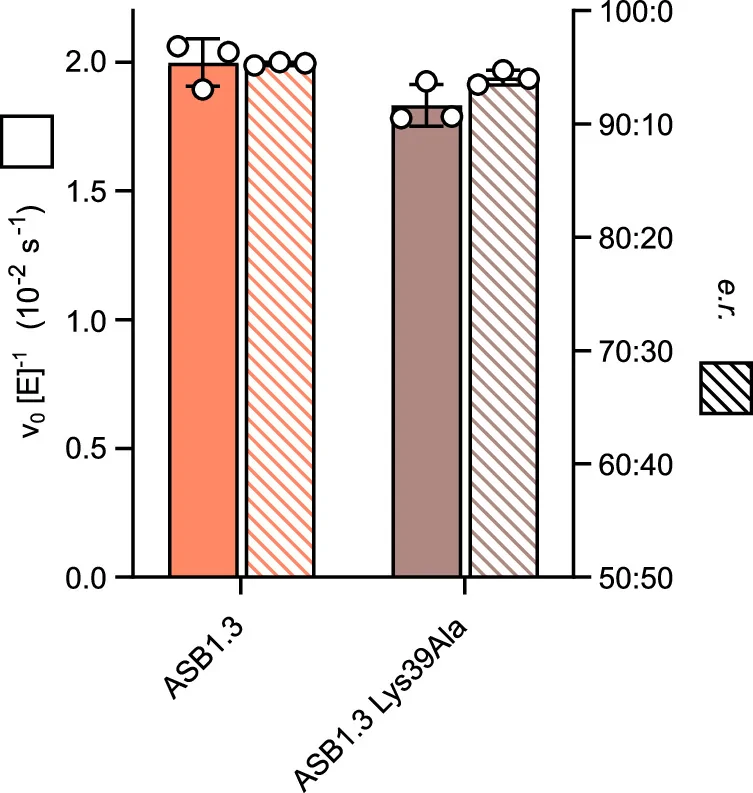
Effect of Lys39Ala mutation on ASB1.3 activity. Bar chart showing the reaction rates (solid bars) and *e.r*. values (hatched bars) for the reaction of **1** and **i** catalysed by ASB1.3 and ASB1.3 Lys39Ala. Reaction rates were measured using 100 µM **1**, 200 µM **i** and 10 µM enzyme in PBS pH 7.4 with 5% MeCN. PNP release was monitored by absorbance at 400 nm. *E.r*. values were determined under identical reaction conditions but using 400 µM **1**, 800 µM **i** and 2 µM enzyme for 30 min. Data are presented as mean values of triplicate measurements, with error bars indicating standard deviation.

**Extended Data Fig. 7:**
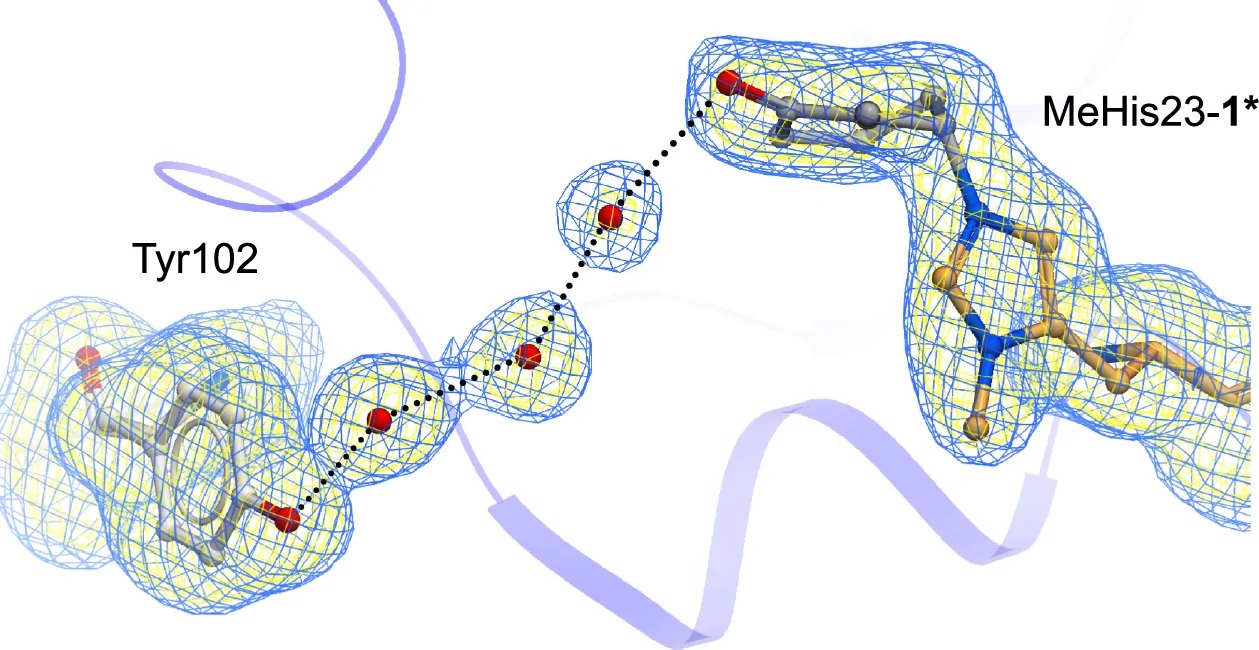
Crystallographic water network between intermediate 1* and Tyr102. 2F_O_-F_C_ electron density map is shown as a mesh contoured at 1.0 σ (blue) and 2.0 σ (yellow).

**Extended Data Fig. 8:**
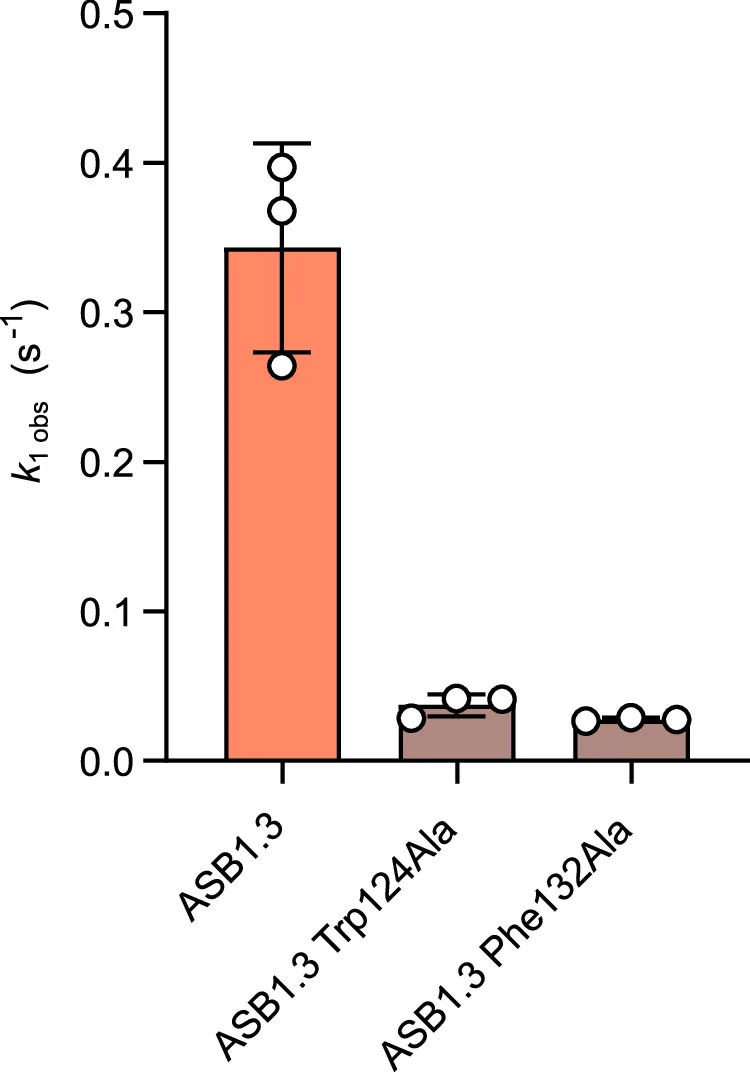
Effect of Trp124Ala and Phe132Ala mutations on intermediate formation rate in ASB1.3. Bar chart showing the rates of formation of intermediate 1* (*k*_1 obs_) for ASB1.3, ASB1.3 Trp124Ala and ASB1.3 Phe132Ala. Reactions were performed using 40 µM **1** and 20 µM enzyme in PBS pH 7.4 with 5% MeCN. PNP release was monitored by absorbance at 400 nm. Data are presented as mean values of triplicate measurements, with error bars indicating standard deviation.

**Extended Data Fig. 9:**
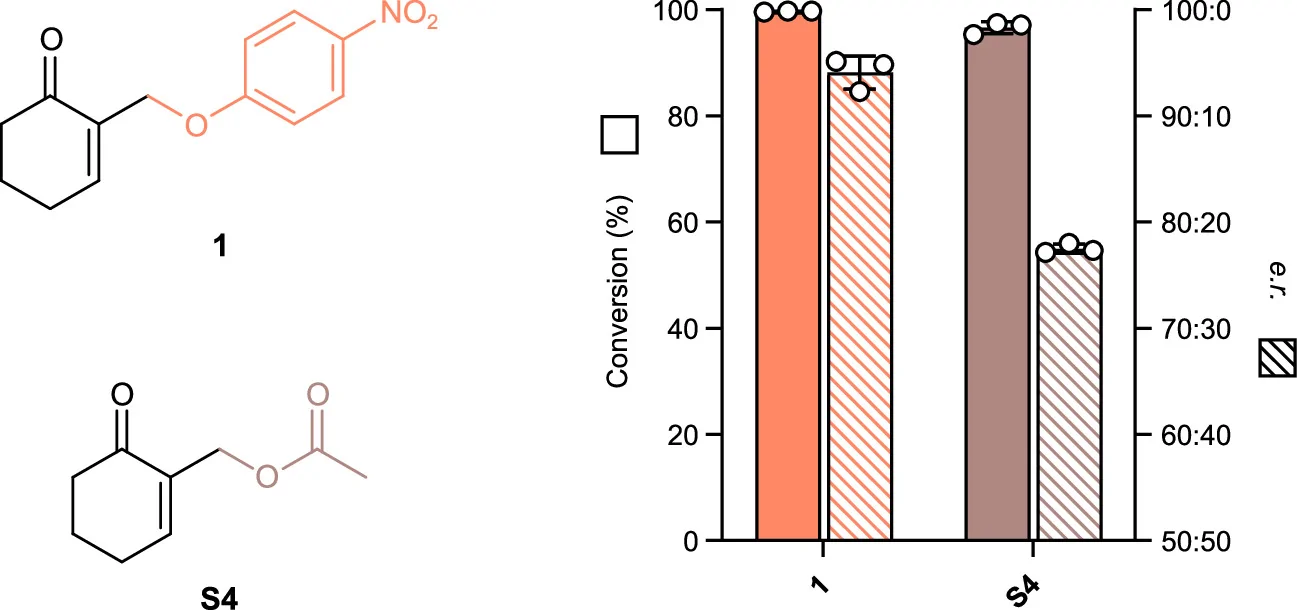
Effect of leaving group identity. Bar chart showing the conversions (solid bars) and *e.r*. values (hatched bars) for the transformation of either **1** or **S4** and **i** to **1i** catalysed by ASB1.3. For **1**, reactions were performed using 400 µM **1**, 800 µM **i** and 5 µM enzyme in PBS pH 7.4 with 5% MeCN for 3 hours. For **S4**, reactions were performed under identical conditions but using **S4** in place of **1** and 20 µM enzyme for 20 hours. Data are presented as mean values of triplicate measurements, with error bars indicating standard deviation.

To gain insights into the catalytic mechanism of our allylic transferases, we solved an X-ray structure of the intermediate state of ASB1.3 (1*–ASB1.3) by soaking apo crystals with substrate **1** (Fig. 5a). Structural analysis was facilitated by the introduction of a K39A surface mutation, which had a negligible effect on activity (Extended Data Fig. 6) and improved data quality. The structure shows the expected electrophilic intermediate bound through Nε of MeHis23 as intended. The intermediate carbonyl is coordinated by Tyr102 via an ordered water network that likely stabilizes anionic intermediates formed along the reaction coordinate (Extended Data Fig. 7). Interestingly, the PNP leaving group derived from **1** is also observed with high occupancy in a hydrophobic pocket shaped by Trp124 and Phe132. Mutation of these aromatic residues to Ala led to substantial reductions in the rate of intermediate formation (around tenfold; Extended Data Fig. 8). The high occupancy of PNP led us to wonder whether selective catalysis proceeds through a ternary complex involving both the leaving group and the incoming nucleophile **i**. To test this hypothesis, we carried out a biotransformation to generate product **1i** using electrophile **S4**, an analogue of **1** equipped with an acetate leaving group in place of PNP (Extended Data Fig. 9). Although high reaction conversion was maintained, reaction with **S4** led to a substantial reduction in enantioselectivity (77:23 e.r. with **S4** versus 94:6 e.r. with **1**), consistent with the PNP ligand playing a role in the selectivity-determining step. Subsequent docking analysis showed that nucleophile **i** can be readily accommodated within the active site in a productive pose for addition to the exocyclic methylene unit of intermediate 1* (Fig. 5b) to afford (*R*)-**1i**, consistent with the experimentally determined product stereochemistry. In contrast, when the PNP leaving group is omitted from the structure, nucleophile **i** docks in a non-productive position within the PNP binding pocket. Together, these observations support a mechanistic model in which the PNP leaving group released during intermediate 1* formation remains bound in the active site pocket and helps to orientate nucleophile **i** for stereoselective C–C bond formation.

## Conclusions

In this study, we introduced a versatile class of activated electrophilic imidazolium intermediates to biocatalysis. These species can be efficiently generated from reagents equipped with a PNP leaving group to facilitate real-time reaction monitoring, rapid substrate profiling and high-throughput directed evolution. Interestingly, structural analysis of our evolved enzymes suggests that PNP may play a role in guiding the selectivity of the downstream chemistry following intermediate formation, highlighting that hydrophobic chromophores commonly used for enzyme engineering can play unexpected roles in catalysis.

Even at an early stage of development, this class of imidazolium intermediates has unlocked a remarkable breadth of chemistry, enabling diverse C–C and C–N bond formations to generate valuable motifs, including γ-butenolides^40^, chiral amines^42^, substituted indoles^43^ and all-carbon quaternary centres^44^. Our evolved enzymes can control the selectivity of reactions involving either prochiral nucleophiles or substituted imidazolium intermediates to generate densely functionalized products featuring Cβ or Cγ stereocentres. Moving forward, there are opportunities to develop enzymes that control the absolute and relative stereochemistry at these two adjacent stereocentres, further diversifying the range of accessible products.

Compared with many transformations involving nucleophilic catalysis, allylic substitutions impose the additional requirement to control the order of catalytic versus substrate nucleophile addition at distinct electrophilic sites in the substrate and intermediate states^34–37^. Our study shows how artificial enzymes are uniquely well suited to address this challenge and allow us to expand upon the range of suitable electrophile and nucleophile coupling partners to achieve transformations that are inaccessible with established small-molecule catalysts. In this way, our study underscores how the remarkable precision achievable with protein catalysts, coupled with their high degree of engineerability, can unlock chemical transformations that would be difficult to achieve with existing methodologies^45–52^.

## Methods

### Materials

All chemicals and biological materials were obtained from commercial suppliers. Lysozyme, DNase I, kanamycin sulfate and chloramphenicol were purchased from Sigma-Aldrich; polymyxin B sulfate from Alfa Aesar; Luria–Bertani (LB) Miller agar, LB Miller medium, 2× yeast extract tryptone (2×YT) medium and arabinose from Formedium; *Escherichia coli* 5α, Q5 DNA polymerase, T4 DNA ligase and restriction enzymes from New England BioLabs; *N*δ-methylhistidine from Bachem; *E. coli* DH10B from Thermo Fisher; and oligonucleotides were synthesized by Integrated DNA Technologies.

### Construction of plasmids

Mutations were inserted into BH_MeHis_1.*x* (ref. ^25^) genes by overlap extension PCR (see Supplementary Table 8 for primer sequences). The genes were subcloned using NdeI and XhoI restriction sites into a pBbE8K vector^53^ containing a carboxy-terminal 6xHis tag. A previously developed pEVOL plasmid containing the PylRS_MeHis_/tRNA_CUA_ system from *ISO4-G1* (ref. ^41^) was used to incorporate MeHis.

### Protein production and purification

For the expression of all enzyme variants, chemically competent *E. coli* DH10B cells containing pEVOL_PylRS_MeHis_/tRNA_CUA_ were transformed with the relevant pBbE8K construct. Single colonies of freshly transformed cells were grown for 18 h in 5 ml LB Miller medium containing kanamycin sulfate (25 µg ml^−1^) and chloramphenicol (25 µg ml^−1^). Starter cultures were used in a 1:100 (v/v) ratio to inoculate 50–400 ml 2×YT medium supplemented with kanamycin sulfate (25 µg ml^−1^), chloramphenicol (25 µg ml^−1^) and MeHis (1 mM). Cultures were grown at 37 °C and 180 r.p.m. to an optical density at 600 nm (OD_600_) between 0.6 and 0.8. Protein expression was induced with (l)-arabinose (final concentration 10 mM) and cultures were incubated for 20 h at 25 °C and 180 r.p.m.

Cells were collected by centrifugation (3,220 *g* for 10 min), resuspended in lysis buffer (50 mM HEPES, 300 mM NaCl, 20 mM imidazole, pH 7.5) and lysed by sonication (1 s on/off pulse, 50% intensity for 10 min). Lysates were clarified by centrifugation (27,216 *g* for 20 min) and supernatants were subjected to affinity chromatography using nickel nitrilotriacetic acid (Ni-NTA) agarose resin (Qiagen). After binding for 1 h at 4 °C with gentle agitation, the resin was drained and washed with 20 ml lysis buffer. Purified protein was eluted using elution buffer (50 mM HEPES, 300 mM NaCl, 250 mM imidazole, pH 7.5).

Proteins were desalted using 10DG desalting columns (Bio-Rad) into PBS buffer (pH 7.4) and analysed by SDS–PAGE. Proteins were aliquoted, flash-frozen in liquid nitrogen and stored at −80 °C. Protein concentrations were determined by measuring the absorbance at 280 nm and using calculated extinction coefficients (ExPASy ProtParam) of 31,400 M^−1^ cm^−1^ for BH_MeHis_1.6, BH_MeHis_1.7, ASB1.1–ASB1.3, ASB1.3 K39A and ASB1.3 F132A; 25,900 M^−1^ cm^−1^ for ASB1.3 W124A; 36,900 M^−1^ cm^−1^ for ASA1.1 and ASA1.2; and 42,400 M^−1^ cm^−1^ for ASA1.3–ASA1.5.

### Mass spectrometry

Purified protein samples were diluted using 0.1% (v/v) acetic acid to a final concentration of 0.5 mg ml^−1^. Mass spectrometry was performed using a 1200 series Agilent LC spectrometer by injection of 5 µl sample into 5% (v/v) MeCN (with 0.1% (v/v) formic acid) and desalted inline for 1 min. Protein was eluted over 1 min using 95% (v/v) MeCN with 5% (v/v) H_2_O. The resulting multiply charged spectrum was analysed using an Agilent QTOF 6510 instrument and deconvoluted using Agilent MassHunter software.

### Site saturation mutagenesis library construction

In each round, 20–24 positions were individually randomized using primers with NNK degenerate codons (for primer sequences, see Supplementary Table 9). DNA libraries were constructed using overlap extension PCR (the templates and targeted positions for each round are listed in Supplementary Tables 1 and 2). The assembled linear library fragments and the pBbE8K vector were digested using *NdeI* and *XhoI* endonucleases, gel purified and ligated using T4 ligase. For the first round of each evolution, existing libraries from the BH_MeHis_1.*x* evolution^25^ were used, with one 96-well plate screened per position. For subsequent rounds, mixed libraries containing three individually randomized positions were generated, each of which was screened over two 96-well plates.

### Shuffling by overlap extension PCR

After each round of evolution, beneficial diversity was combined by DNA shuffling of fragments generated by overlap extension PCR. Primers were designed to encode either the parent amino acid or the identified mutation and used to generate short fragments (up to five), which were gel-purified and mixed appropriately in overlap extension PCR to generate genes for all possible combinations of mutants. Genes were cloned as described above.

### Library screening

For protein expression and screening, all transfer and aliquoting steps were performed using Hamilton liquid-handling robots. Chemically competent *E. coli* DH10B cells containing pEVOL_PylRS_MeHis_/tRNA_CUA_ were transformed with the library plasmids. Freshly transformed clones were used to inoculate 150 μl 2×YT medium supplemented with kanamycin sulfate (25 μg ml^−1^) and chloramphenicol (25 μg ml^−1^) in Corning Costar 96-well microtitre round-bottom plates. As controls, each plate contained six clones of the parent template and two clones containing an empty pBbE8k vector. Plates were incubated overnight at 30 °C, 80% humidity and 850 r.p.m. Then, 20 µl of overnight culture was used to inoculate 480 μl 2×YT medium supplemented with kanamycin sulfate (25 μg ml^−1^), chloramphenicol (25 μg ml^−1^) and MeHis (1 mM) in 96-deep-well plates. The cultures were incubated at 30 °C, 80% humidity and 850 r.p.m. until an OD_600_ between 0.6 and 0.8 was reached. The cultures were induced with (l)-arabinose (final concentration 10 mM) and incubated for 20 h at 30 °C, 80% humidity and 850 r.p.m. Cells were collected by centrifugation (3,220 *g* for 10 min) and the supernatant discarded. The pelleted cells were resuspended in 400 μl lysis buffer (PBS (pH 7.4) supplemented with lysozyme (1.0 mg ml^−1^), polymixin B (0.5 mg ml^−1^) and DNase I (10 μg ml^−1^)) and incubated for 2 h at 30 °C, 80% humidity and 850 r.p.m. Cell debris was removed by centrifugation (3,220 *g* for 10 min).

#### ASB round 1

For ASB round 1, 80 μl clarified lysate was transferred to 96-well microtitre plates. Then, 100 μl of freshly prepared PBS (pH 7.4) containing MeCN and nucleophile **i** (final concentration 200 μM) was added and reactions were initiated by the addition of 20 μl PBS (pH 7.4) containing MeCN and electrophile **1** (final concentration 100 μM, final MeCN concentration 5% (v/v)). Reaction progression was monitored spectrophotometrically at 400 nm over 30 min using a CLARIOstar plate reader (BMG Labtech). Initial reaction rates for individual variants were normalized to the average of the six parent control wells on each plate.

#### ASB rounds 2 and 3

Screening proceeded as in ASB round 1 with reduced volumes of lysate (round 2: 50 μl; round 3: 60 μl) and nucleophile solutions adjusted to maintain identical final concentrations of reactants. The reaction time was also reduced to 15 min.

#### ASA round 1

For ASA round 1, 150 μl clarified lysate was transferred to 96-well microtitre plates. Then, 30 μl PBS (pH 7.4) containing MeCN and nucleophile **ii** (final concentration 1,000 μM) was added and reactions were initiated by the addition of 20 μl PBS (pH 7.4) containing MeCN and electrophile **2** (final concentration 100 μM, final MeCN concentration 5% (v/v)). Reaction progression was monitored spectrophotometrically as described above over 30 min.

#### ASA rounds 2–5

Screening proceeded as in ASA round 1 with reduced volumes of lysate (round 2: 130 μl; rounds 3–5: 100 μl) and nucleophile solutions adjusted to maintain identical final concentrations of reactants.

Following each round, the top (~1%) clones were rescreened in triplicate (Supplementary Fig. 7). Expression and screening were performed as described above but from glycerol stocks prepared from the original overnight cultures. Confirmed hits were evaluated in purified protein before shuffling.

### General procedure for analytical-scale biotransformations

Analytical-scale biotransformations were performed in PBS (pH 7.4) with 5% (v/v) MeCN using the concentrations of reactants and enzymes stated. The reactions were incubated with shaking at 800 r.p.m. at the stated temperature. For reversed-phase ultra-performance liquid chromatography (UPLC) analysis, reactions were quenched at the stated time by the addition of MeCN (1 volume), vortexed and the precipitated proteins removed by centrifugation (14,000 *g* for 10 min). For normal-phase HPLC analysis, reactions were quenched similarly but with EtOAc (1 volume). Next, 0.5 M NaOH (0.2 volume) was added after quenching to the biotransformations yielding product **3iii** to retain PNP in the aqueous phase. The organic phase was then separated and directly injected into the HPLC instrument. For GC analysis, reactions were quenched similarly with EtOAc (1.5 volume). The organic phase was then separated and dried over MgSO_4_, clarified by centrifugation (14,000 *g* for 10 min) and the supernatant injected into the GC instrument.

### Total turnover numbers

For ASB1.3, biotransformations containing **1** (200 µM), **i** (400 µM) and ASB1.3 (0.5 or 0.25 mol%) in PBS (pH 7.4) with 5% (v/v) MeCN were incubated at 30 °C with shaking at 800 r.p.m. Samples were taken each hour for 8 h. After each sample extraction, an additional 2 equiv. **i** was added to the reaction (2–5 µl of a 100 mM solution in MeCN). Samples were quenched by the addition of MeCN (1 volume), vortexed and the precipitated proteins removed by centrifugation (14,000 *g* for 10 min). The supernatants were then analysed by reversed-phase UPLC.

For ASA1.5, biotransformations containing **2** (100 µM), **ii** (200 µM) and ASA1.5 (2, 1 or 0.5 mol%) in PBS (pH 7.4) with 5% (v/v) MeCN were incubated at 30 °C with shaking at 800 r.p.m. Samples were taken at 2, 4, 22, 46 and 51 h, quenched by the addition of MeCN (1 volume), vortexed and the precipitated proteins removed by centrifugation (14,000 *g* for 10 min). The supernatants were then analysed by reversed-phase UPLC.

### Spectrophotometric assays

To monitor slow-to-moderate reaction profiles (*k*_obs_ ≲ 0.5 s^−1^), 200 µl biotransformations were performed in clear 96-well microplates in PBS (pH 7.4) with 5% (v/v) MeCN using the concentrations of reactants and enzymes stated. Using a CLARIOstar plate reader (BMG Labtech), reactions were initiated by the addition of a 5× stock of electrophile in PBS (pH 7.4) with 20% (v/v) MeCN using the autoinjector. The absorbance at 400 nm was measured over an appropriate time interval and converted to PNP concentration using a standard curve. For each reaction condition, traces from triplicate control reactions with no enzyme were averaged and subtracted from each enzyme reaction and any pre-injection offset was adjusted to 0.

To monitor fast reaction profiles (*k*_obs_ ≳ 0.5 s^−1^), biotransformations were performed in PBS (pH 7.4) with 5% (v/v) MeCN on a stopped-flow spectrophotometer (TgK Scientific) equipped with a tungsten halogen lamp. Single mixing experiments were performed using 2× stock solutions of enzyme and substrate in the drive syringes and the absorbance was measured at 400 nm.

To calculate intermediate formation rates, reactions containing electrophile and enzyme but no exogenous nucleophile were fitted to a modified single exponential model (equation (1)):

1 $$[\mathrm{PNP}]=(1-{{\rm{e}}}^{-bt})(a+mt)$$

where [PNP] is the observed PNP concentration, *t* is time, *b* is the apparent first-order rate constant, *a* is the apparent enzyme concentration and *m* is the rate of hydrolytic turnover.

Catalytic turnover rates were calculated from reactions containing electrophile, exogenous nucleophile and enzyme. UPLC was used to confirm that the amount of PNP released correlated 1:1 with the amount of product formed (Supplementary Fig. 8). The gradient at the start of the linear turnover phase of each reaction was calculated and divided by the enzyme concentration to give the turnover rate.

### Chromatographic analysis

Reversed-phase UPLC analysis was performed on a 1290 Infinity II Agilent LC system with a Kinetix 5 μm XB-C18 100 Å LC column (50 mm × 2.1 mm; Phenomenex). LC–MS analysis was performed on an identical system coupled to an Agilent 6135b single quadrupole mass spectrometer in positive mode. The separation methods used for all substrates and products are reported in Supplementary Table 4 and the extinction coefficients used to calculate conversions are provided in Supplementary Table 5. Peaks were assigned by comparison with synthesized standards and peak areas were integrated using Agilent’s OpenLab software.

Chiral analysis was performed on a normal-phase 1260 Agilent HPLC system with either a ChiralPak 3 μm IG-3 SFC column (50 mm × 3 mm) or a ChiralPak 3 μm IA-3 SFC column (50 mm × 4.6 mm; Daicel). The separation methods used for all product enantiomers are reported in Supplementary Table 6. For product **2ii**, chiral analysis was performed on a 7890A Agilent GC system equipped with a flame ionization detector (FID), a 7693 autosampler and a CP-Chirasil-DEX-CB column (25 m × 0.32 mm, film thickness 0.25 µm). The injector was maintained at 180 °C and used in splitless mode. Helium was used as the carrier gas with a flow rate of 1 ml min^−1^ and a pressure of 93 kPa. The temperature programme was started at 120 °C with a hold for 5 min, followed by an increase to 190 °C at a rate of 5 °C min^−1^ with a hold at 190 °C for 5 min. The FID was maintained at 200 °C with a flow of hydrogen of 30 ml min^−1^. Peaks were assigned by comparison with synthesized standards and peak areas were integrated using Agilent’s OpenLab software.

### Preparative-scale biotransformations

Butenolide **1i** was synthesized on a preparative scale (300 ml) using **1** (29.7 mg, 400 µM), **i** (37.5 mg, 800 µM) and ASB1.3 (2 µM) in PBS (pH 7.4) with 10% (v/v) MeCN. The reaction was incubated at 4 °C with rocking at 60 r.p.m. and aliquots (100 µl) were removed hourly to monitor the progress of the reaction by UPLC analysis. At 4.5 h, an additional 2 equiv. **i** was added (37.5 mg). At 6.5 h, the reaction had proceeded to 98% conversion and was quenched with MeCN (300 ml). The mixture was then filtered and concentrated under reduced pressure to ~250 ml and the product extracted with EtOAc (4 × 100 ml). The combined organic extracts were washed with brine (5 × 50 ml) interspersed with 0.5 M NaOH washes (4 × 50 ml) followed by a final brine wash (50 ml), dried over MgSO_4_, filtered and concentrated under reduced pressure. The crude product was purified by silica column chromatography (column 1: neat dichloromethane (DCM) to 1:49 MeOH–DCM; column 2: 5:1 to 1:1 cyclohexane–EtOAc) to give 5-[(6-oxocyclohex-1-en-1-yl)methyl]furan-2(5*H*)-one (**1i**) as a brown oil (15.8 mg, 69%). ^1^H NMR (400 MHz, CDCl_3_): *δ* = 7.41 (dd, *J* = 5.7, 1.5 Hz, 1 H), 6.93 (t, *J* = 4.2 Hz, 1 H), 6.03 (dd, *J* = 5.7, 2.0 Hz, 1 H), 5.23–5.15 (m, 1 H), 2.73 (dd, *J* = 13.9, 4.4 Hz, 1 H), 2.55 (dd, *J* = 13.9, 6.8 Hz, 1 H), 2.49–2.34 (m, 4 H), 2.07–1.89 ppm (m, 2 H). ^13^C NMR (101 MHz, CDCl_3_): *δ* = 199.4, 173.2, 156.4, 150.9, 133.1, 121.5, 81.8, 38.3, 33.2, 26.4, 23.0 ppm.

Methyl acrylate **2ii** was synthesized on a preparative scale (320 ml) using **2** (30.4 mg, 400 µM), **ii** (32.8 mg, 800 µM) and ASA1.5 (5 µM) in PBS (pH 7.4) with 10% (v/v) MeCN. The reaction was incubated at 4 °C with rocking at 60 r.p.m. and aliquots (100 µl) were removed daily to monitor the progress of the reaction by UPLC analysis. At 192.5 h, the reaction had proceeded to 98% conversion and was quenched with MeCN (320 ml). The mixture was worked up as described above and the crude product was purified by silica column chromatography (3:1 cyclohexane–EtOAc) to give methyl 2-[(3-acetyl-2-oxotetrahydrofuran-3-yl)methyl]acrylate (**2ii**) as a white powder (19.7 mg, 68%). ^1^H NMR (400 MHz, CDCl_3_): *δ* = 6.30 (s, 1 H), 5.67 (s, 1 H), 4.26 (td, *J* = 8.8, 3.2 Hz, 1 H), 4.14 (td, *J* = 9.0, 7.0 Hz, 1 H), 3.74 (s, 3 H), 3.28–3.19 (m, 1 H), 2.88–2.77 (m, 2 H), 2.36 (s, 3 H), 2.07 ppm (dt, *J* = 13.2, 8.8 Hz, 1 H). ^13^C NMR (101 MHz, CDCl_3_): *δ* = 201.9, 175.3, 167.1, 135.3, 129.6, 66.6, 61.3, 52.4, 35.6, 29.2, 26.0 ppm.

### Circular dichroism spectroscopy

UV–visible circular dichroism (CD) spectra were acquired using an Applied Photophysics Chiroscan CD instrument. Samples were prepared at the stated concentrations in DCM and loaded into a 1-mm pathlength quartz cuvette. Absorbance and CD spectra were acquired between 240 and 350 nm at 1-nm intervals with an accumulation time of 10 s per interval. Baseline correction of the spectra was performed by subtraction of the spectra of the solvent recorded under identical conditions.

### Absolute configuration determination by VCD spectroscopy

#### Experimental details

Infrared (IR) and VCD spectra were recorded on a Bruker Vertex 70/PMA 50 VCD spectrometer at 4 cm^−1^ spectral resolution by accumulating 32 scans for the IR analysis and ~100,000 scans (23 h accumulation time) for the VCD analysis. Samples were dissolved in CDCl_3_ at the concentrations given in the respective legends and measured using a BaF_2_ IR cell with an optical path length of 100 μm. Baseline correction of the VCD spectra was performed by subtraction of the spectra of the solvent recorded under identical conditions.

#### Computational details

Absolute configurations were derived from the experimental spectra by computation of IR and VCD spectra. Therefore, conformational sampling was carried out based on a systematic search algorithm at the Merck molecular force field level^54,55^. All conformers thus obtained were subjected to further geometry optimization at the B3LYP/6-311++G(d,p)/IEFPCM(CHCl_3_) level of theory using Gaussian 09 (Rev E.01)^56^. For final comparison with experiment, the IR and VCD spectra were simulated from the single-conformer spectra using the zero-point corrected energy (Δ*E*_ZPC_)-based Boltzmann weights and by assigning a Lorentzian band shape with a half width at half height of 10 cm^−1^ to the computed dipole and rotational strength.

### Protein crystallization, refinement and model building

Mutating residue 39 to Ala in ASB1.3 was found to improve the resolution of the corresponding diffraction data^50^ while having a minimal effect on the reaction rate or e.r. value (Extended Data Fig. 6). Crystals of ASB1.3 K39A were prepared by mixing 200 nl of 10 mg ml^−1^ protein in 50 mM HEPES (pH 7.0) with an equal volume of precipitant. Crystallization conditions were identified through a basic chemical space (BCS) screen (Molecular Dimensions): 0.1 M sodium citrate, 0.15 M ammonium acetate and 15% (v/v) polyethylene glycol (PEG) Smear High (pH 5.0, BCS C7). All trials were conducted by sitting-drop vapour diffusion and incubated at 4 °C. Crystals were cryoprotected in reservoir solution supplemented with 10% (v/v) PEG 200 and flash-cooled in liquid N_2_. For soaking with **1**, 2 µl of a 20 mM solution of **1** in MeCN was added to the reservoir, then 2 µl of the reservoir was added to the drop before freezing. All data were collected at the Diamond Light Source (Harwell, UK). Data reduction was performed with Diffraction Integration for Advanced Light Sources (DIALS) and the structure solved by molecular replacement using a search model derived from PDB 8BP0 (ref. ^25^). Iterative rounds of model building and refinement were performed in Crystallographic Object-Oriented Toolkit (COOT) and Phenix.refine, respectively^57^. Validation with MolProbity^58^ and PDB-REDO^59^ were incorporated into the iterative rebuild and refinement process. Data collection and refinement statistics are provided in Supplementary Table 7. Docking studies were performed with ICM-Pro^60^.

### Reporting summary

Further information on research design is available in the Nature Portfolio Reporting Summary linked to this article.

## Supplementary information

Supplementary Information Supplementary Figs. 1–10, Tables 1–9, Methods (chemical synthesis, DNA and protein sequences, chiral HPLC chromatograms) and references.

Reporting Summary

Supplementary Data 1 Computed conformers for (*R*)-**1i**, (*S*)-**2ii** and (*R*)-**3**.

## Source data

Source Data Fig. 4 Source data for Fig. 4a,b.

## Data Availability

Coordinates and structure factors have been deposited in the Protein Data Bank under accession number 9R2D. The data supporting the findings of this study are available within the article and its Supplementary Information. Data are also available from the corresponding authors upon request. Source data are provided with this paper.
