# Supplementary material for: Protein-confined imidazolium intermediates enable diverse biocatalytic C–C and C–N bond formations
Source: Nat Catal. 2026 Jul 31;9(8):882–92. doi: 10.1038/s41929-026-01587-8 (PMC13493319; doi:10.1038/s41929-026-01587-8)
Supplement: Supplementary file 1 [file 41929_2026_1587_MOESM1_ESM.pdf]

nature catalysis

Supplementary information

https://doi.org/10.1038/s41929-026-01587-8

Protein-confined imidazolium intermediates enable diverse biocatalytic C–C and C–N bond formations

In the format provided by the authors and unedited

Contents

Supplementary Figures 2

Supplementary Fig. 1: Divergent regiochemical outcomes of allylic substitution reactions 2

Supplementary Fig. 2: NMR traces of purified **1i** produced from a preparative-scale biotransformation 3

Supplementary Fig. 3: Absolute configuration determination by VCD spectroscopy 4

Supplementary Fig. 4: NMR traces of purified **2ii** produced from a preparative-scale biotransformation 5

Supplementary Fig. 5: Nucleophiles used in substrate screening 6

Supplementary Fig. 6: Structural modelling to predict stereochemistry of products derived from electrophile **3** 7

Supplementary Fig. 7: Retest plate data for the directed evolution of ASB1.3 and ASA1.5 9

Supplementary Fig. 8: Molar ratio between product formed and PNP released 10

Supplementary Fig. 9: ^{1}H-^{1}H COSY (400 MHz, $CDCl_3$) of product **1v** 11

Supplementary Fig. 10: Absorbance and CD spectra of products **1xiii** and **3iii** 12

Supplementary Tables 13

Supplementary Table 1: Directed evolution of ASB1.3 13

Supplementary Table 2: Directed evolution of ASA1.5 14

Supplementary Table 3: Reaction conditions for the substrate scope shown in Fig. 4 15

Supplementary Table 4: Reverse phase UPLC analysis methods 16

Supplementary Table 5: Extinction coefficients of electrophiles and products 17

Supplementary Table 6: Normal phase chiral HPLC analysis methods 18

Supplementary Table 7: Data collection and refinement statistics 19

Supplementary Table 8: Primers used in this study 20

Supplementary Table 9: Primers used for library generation and shuffling 21

Supplementary Methods 25

Chemical Synthesis 25

DNA and Protein Sequences for ASB1.3 and ASA1.5 34

Chiral HPLC Chromatograms 35

Supplementary References 45

Supplementary Figures

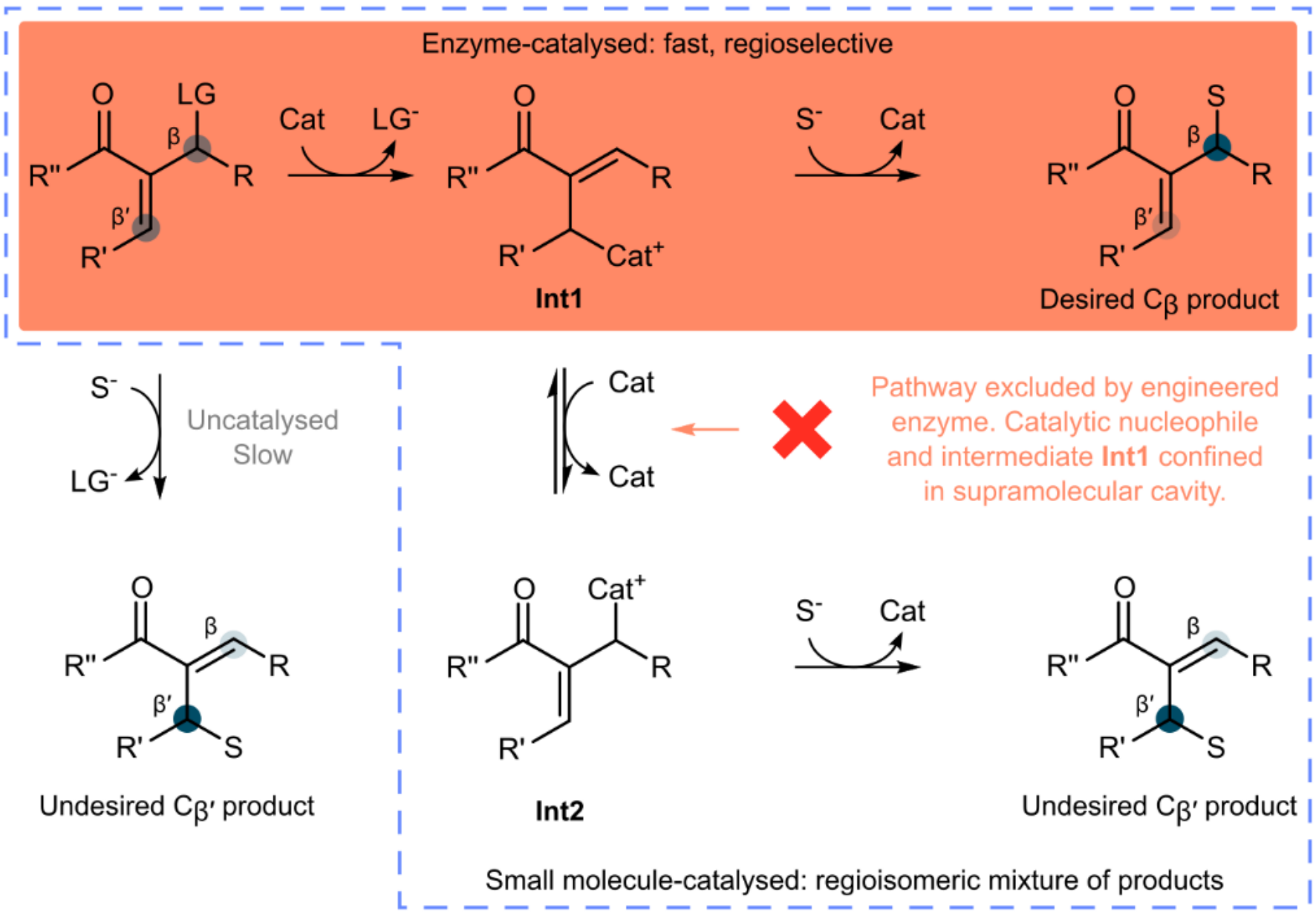

Supplementary Fig. 1: Divergent regiochemical outcomes of allylic substitution reactions. Proposed reaction pathways for allylic substitution reactions. Reactions performed with small catalytic nucleophiles (blue dashed outline) commonly lead to a mixture of regioisomeric C_{β} and $C_{\beta'}$ products (see references 34-37 from main text), which can arise from addition of substrate nucleophile (S) to intermediates **Int1** or **Int2**, respectively. Engineered allylic substitution enzymes promote regioselective transformations to generate C_{β} products by accelerating the pathway highlighted in orange. Furthermore, the competing pathway leading to **Int2** is inaccessible due to confinement of the catalytic nucleophile and **Int1** within a supramolecular protein cavity.

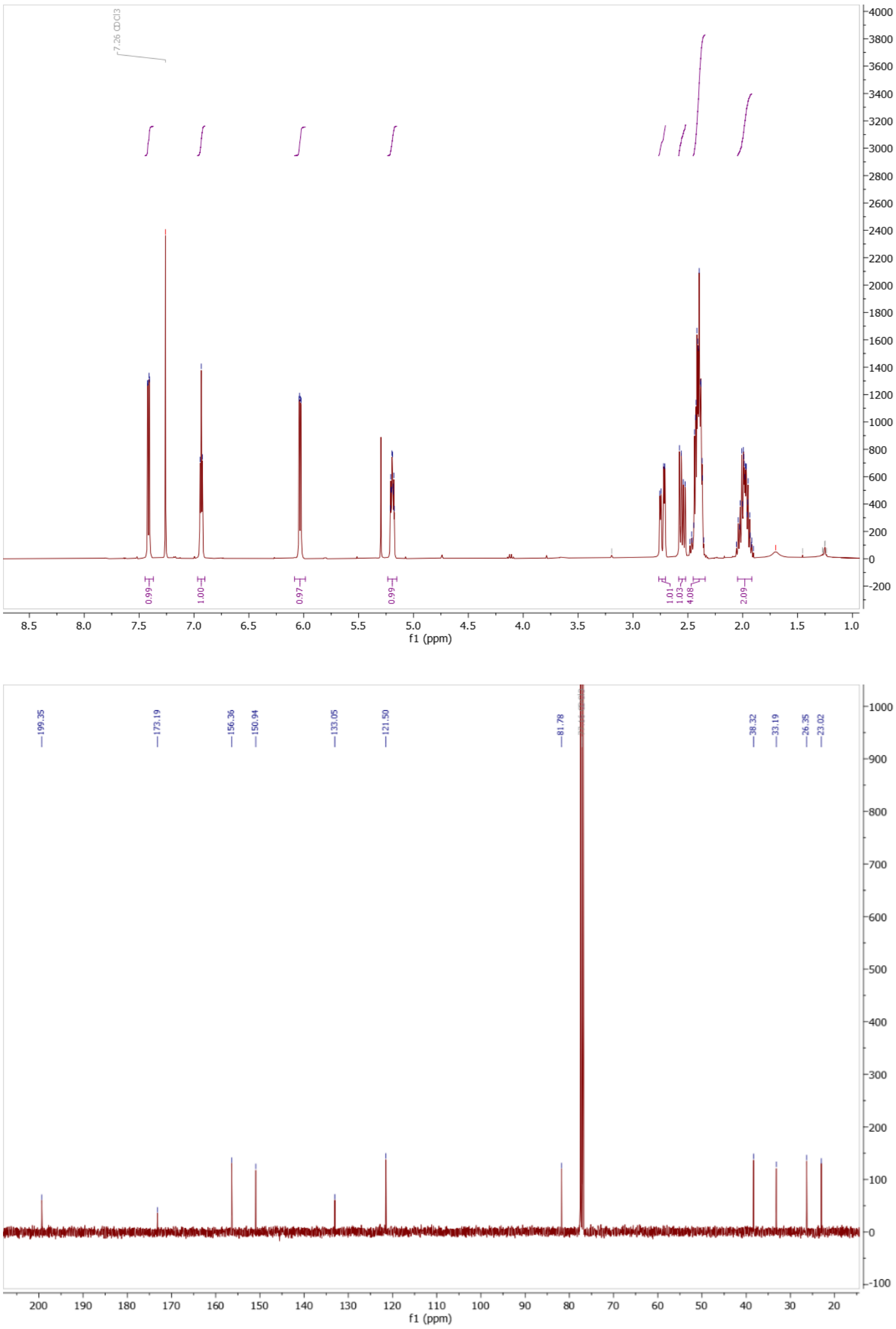

Supplementary Fig. 2: NMR traces of purified 1i produced from a preparative-scale biotransformation. ^{1}H NMR (400 MHz, $CDCl_3$, top) and ^{13}C NMR (101 MHz, $CDCl_3$, bottom) of product **1i** extracted from a preparative-scale biotransformation and purified by silica column chromatography.

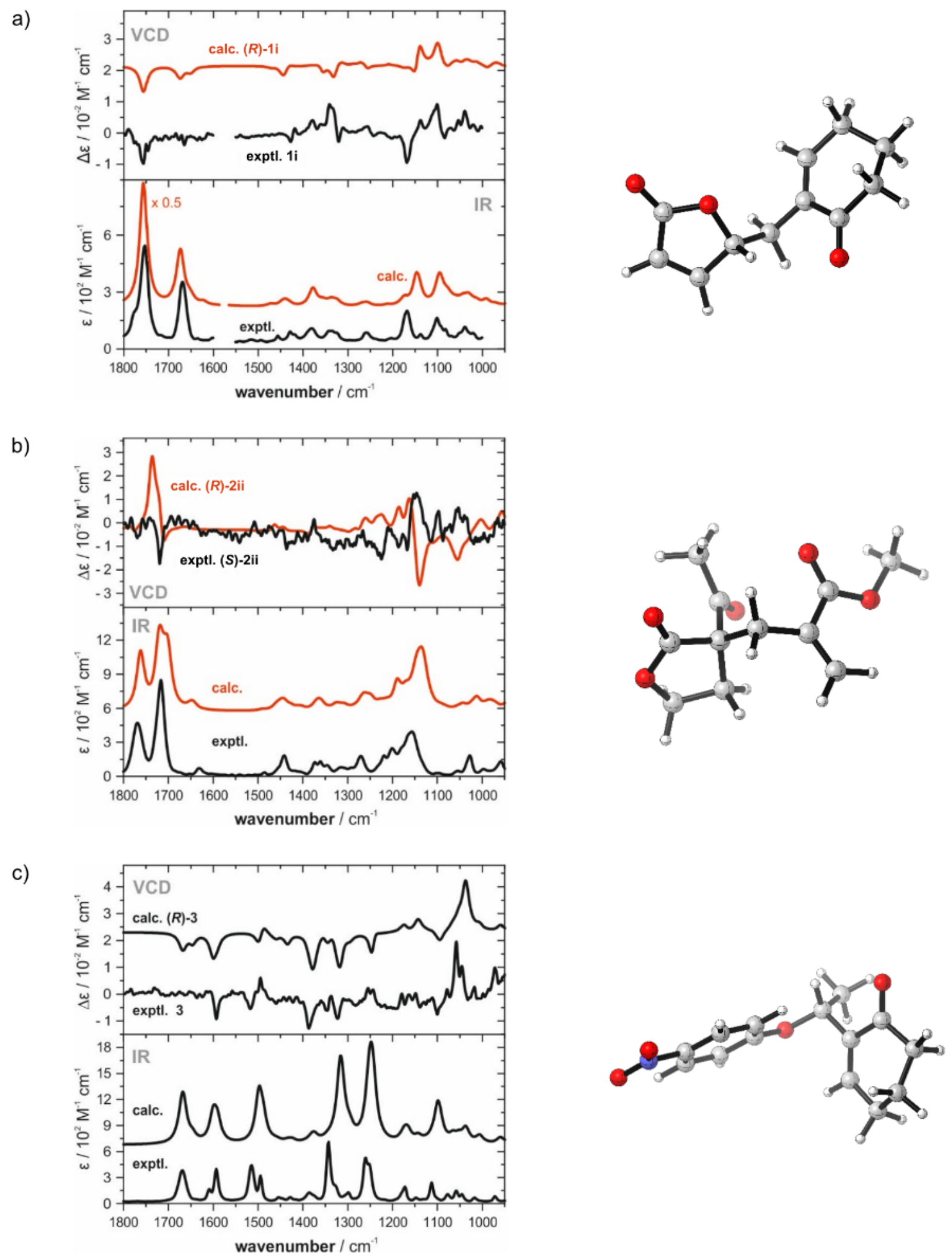

Supplementary Fig. 3: Absolute configuration determination by VCD spectroscopy. a) Left: comparison of the experimental (0.17 M for C=O region, 0.36 M for <1500 cm^{-1}, $CDCl_3$, 100 µm) and computed IR and VCD spectra of **1i**. Right: lowest energy conformation of (*R*)-**1i** (population of 54%, see Supplementary Data 1). **b)** Left: comparison of the experimental (0.088 M, $CDCl_3$, 100 µm) and computed IR and VCD spectra of **2ii**. A comparison is presented between the experimental spectra of (*S*)-**2ii** and the computed spectra of (*R*)-**2ii** for clarity. Right: lowest energy conformation of (*S*)-**2ii** (population of 20%, see Supplementary Data 1). **c)** Left: comparison of the experimental (0.19 M, $CDCl_3$, 50 µm) and computed IR and VCD spectra of **3**. Right: lowest energy conformation of (*R*)-**3** (population of 80%, see Supplementary Data 1).

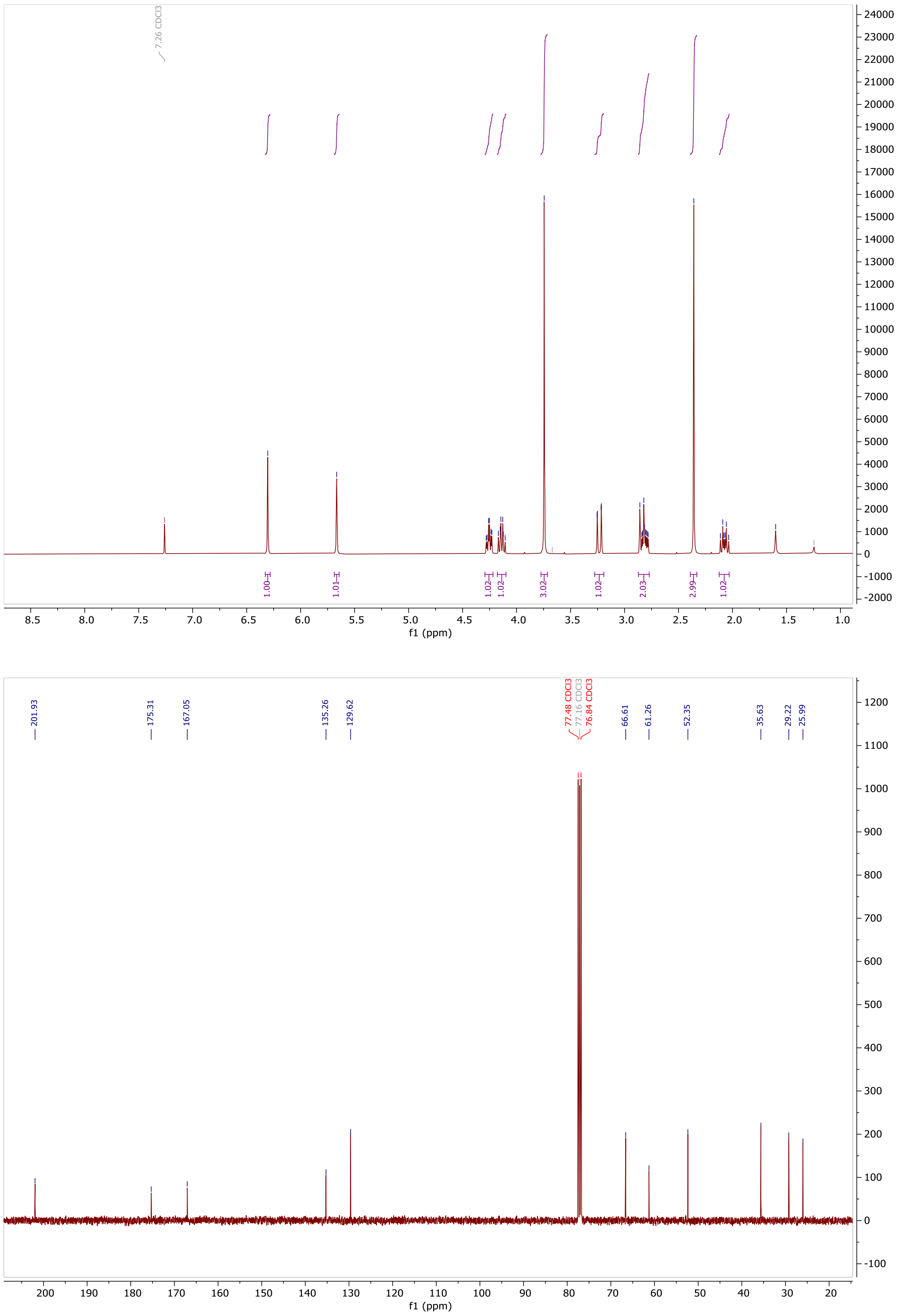

Supplementary Fig. 4: NMR traces of purified 2ii produced from a preparative-scale biotransformation. ^{1}H NMR (400 MHz, $CDCl_3$, top) and ^{13}C NMR (101 MHz, $CDCl_3$, bottom) of product **2ii** extracted from a preparative-scale biotransformation and purified by silica column chromatography.

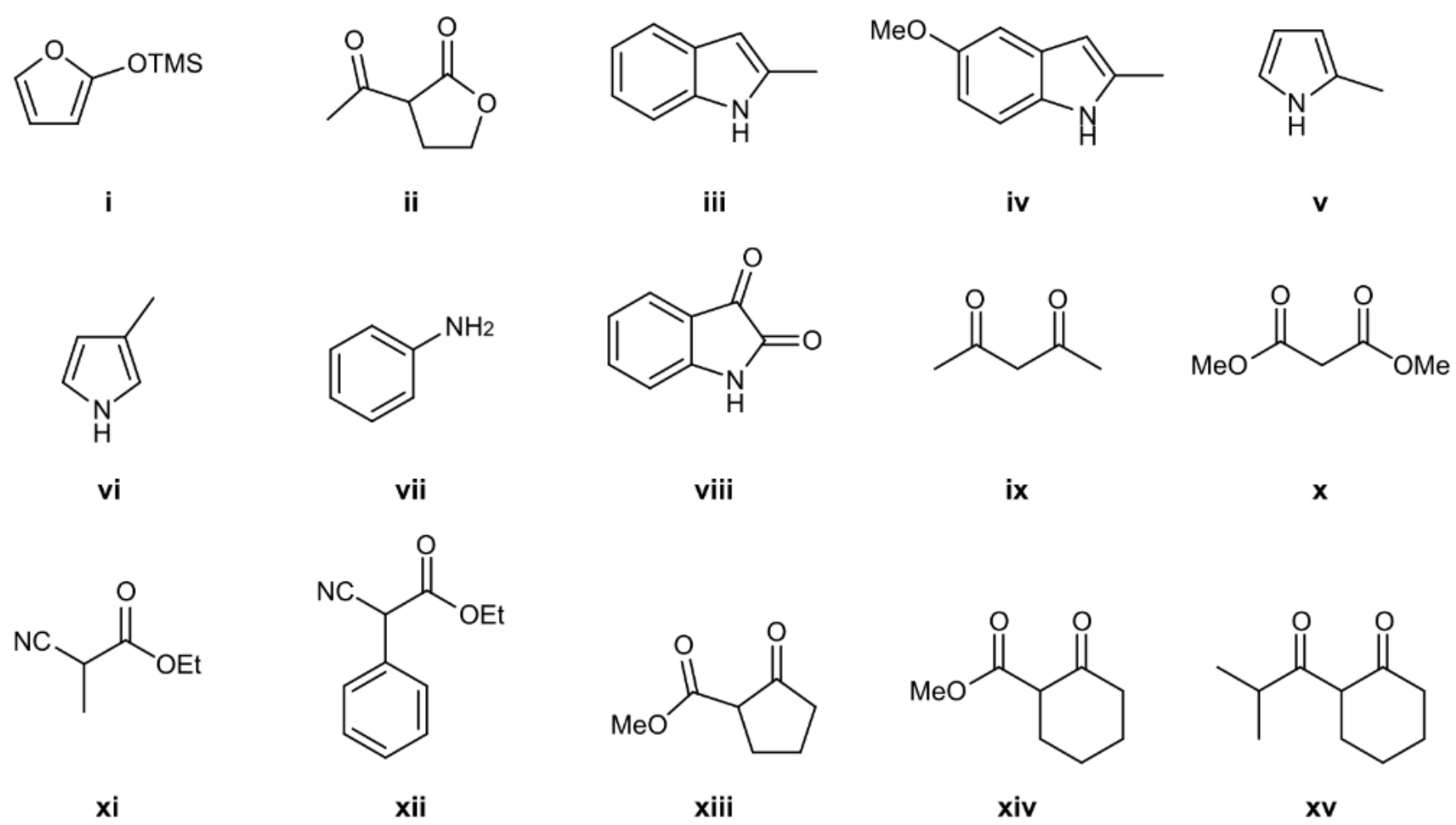

Supplementary Fig. 5: Nucleophiles used in substrate screening. For nucleophiles containing chiral centres, racemates were used.

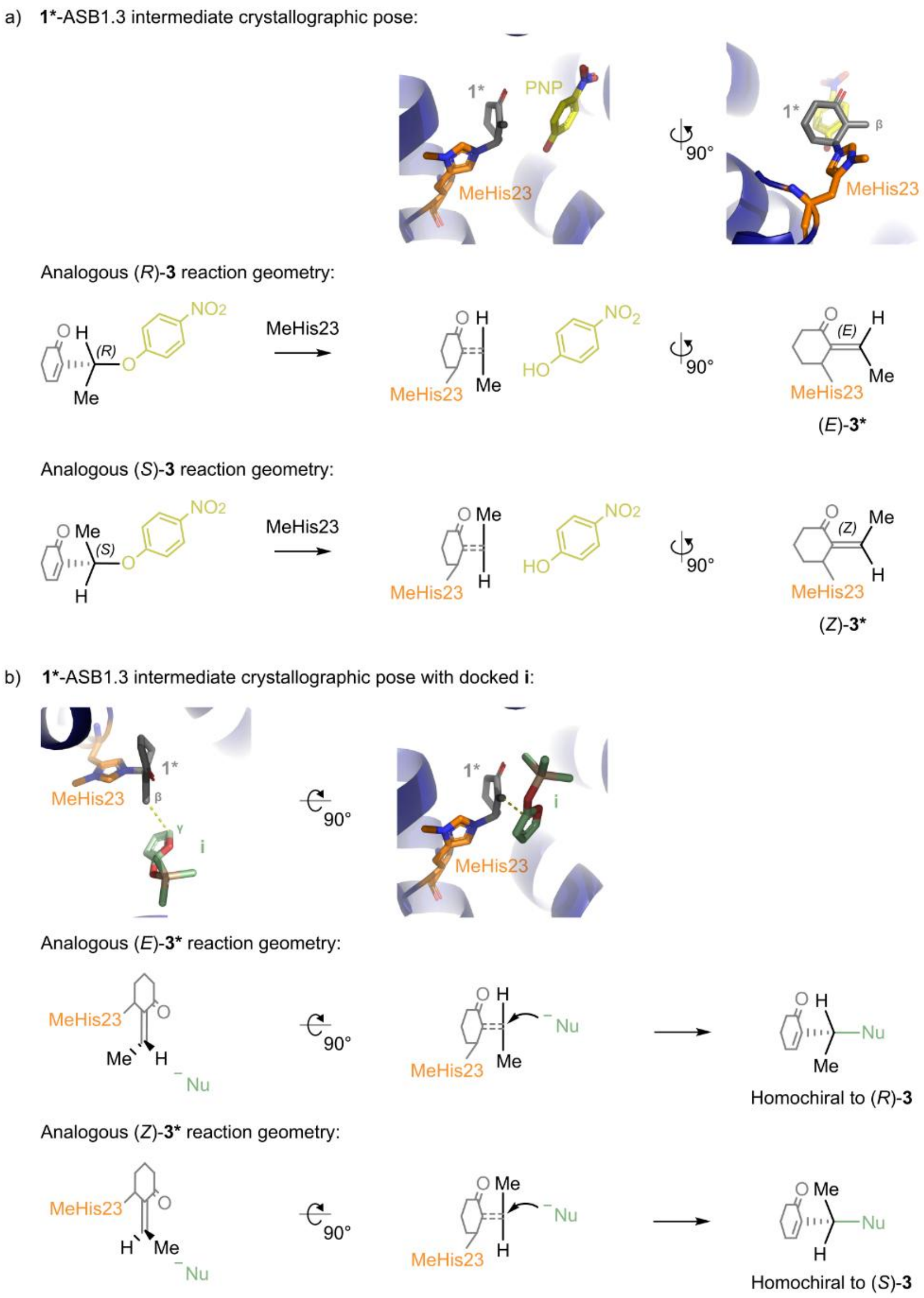

Supplementary Fig. 6: Structural modelling to predict stereochemistry of products derived from electrophile 3. a) The **1***-ASB1.3 crystal structure shows the PNP chromophore binding in a pocket adjacent to intermediate **1*** (top panels). If electrophile **3** binds in an analogous geometry, with the cyclohexenone and PNP moieties in the same positions as in the crystal structure, then in (*R*)-**3** the methyl group will point down (towards MeHis23, middle-left structure) and in (*S*)-**3** the methyl group will point up (away from MeHis23, bottom-left structure). Reaction of **3** with MeHis23 leads to the elimination of PNP, converting C_β into an sp^2 centre. Assuming the up/down positioning of the methyl group is maintained, the geometry of (*R*)-**3** will lead to the (*E*) isomer of **3*** (middle scheme), and (*S*)-**3** will lead to the (*Z*) isomer of **3*** (bottom scheme). **b)** Nucleophile addition to intermediate **1*** or **3*** reverts C_β to an sp^3 centre. The docking pose of nucleophile **i** in the **1***-ASB1.3 crystal structure is indicative of

addition to **1*** on the same face as PNP elimination (top panels; dotted line indicates direction of addition between C_γ of **i** and C_β of **1***). In the analogous **3*** reaction geometries, if nucleophile addition occurs on the same face as PNP elimination then addition to (*E*)-**3*** will result in products homochiral to (*R*)-**3** (middle scheme), and addition to (*Z*)-**3*** will result in products homochiral to (*S*)-**3** (bottom scheme).

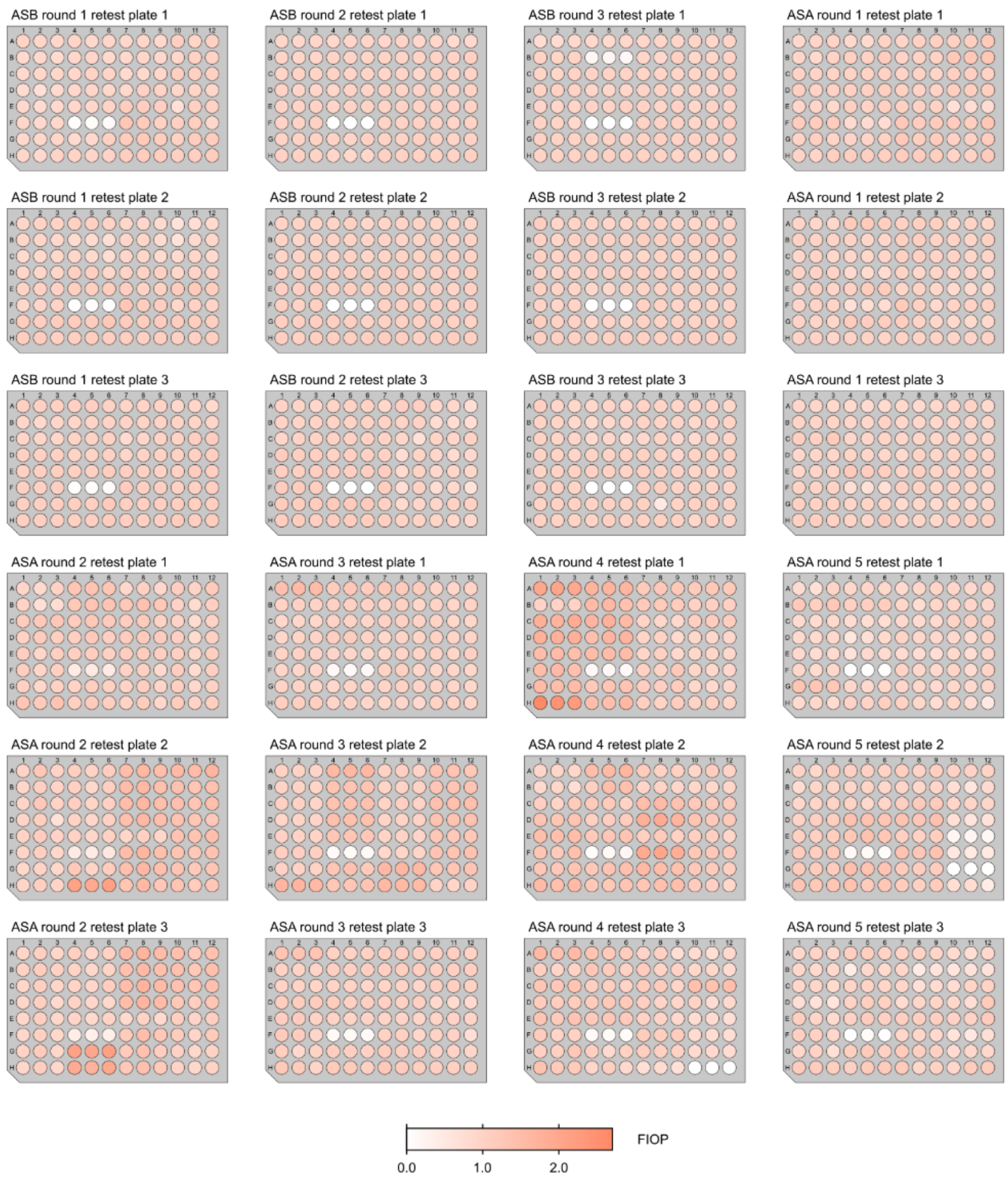

Supplementary Fig. 7: Retest plate data for the directed evolution of ASB1.3 and ASA1.5. Each retest plate consists of variants arrayed in horizontal triplicates (e.g. A1-3). For all plates, wells B1-3 and E7-9 were positive controls (the parent sequence for each round), and wells F4-6 were negative controls (empty vector). Initial reaction velocities are shown as fold improvements over parent (FIOPs) normalised to the positive control mean on each plate.

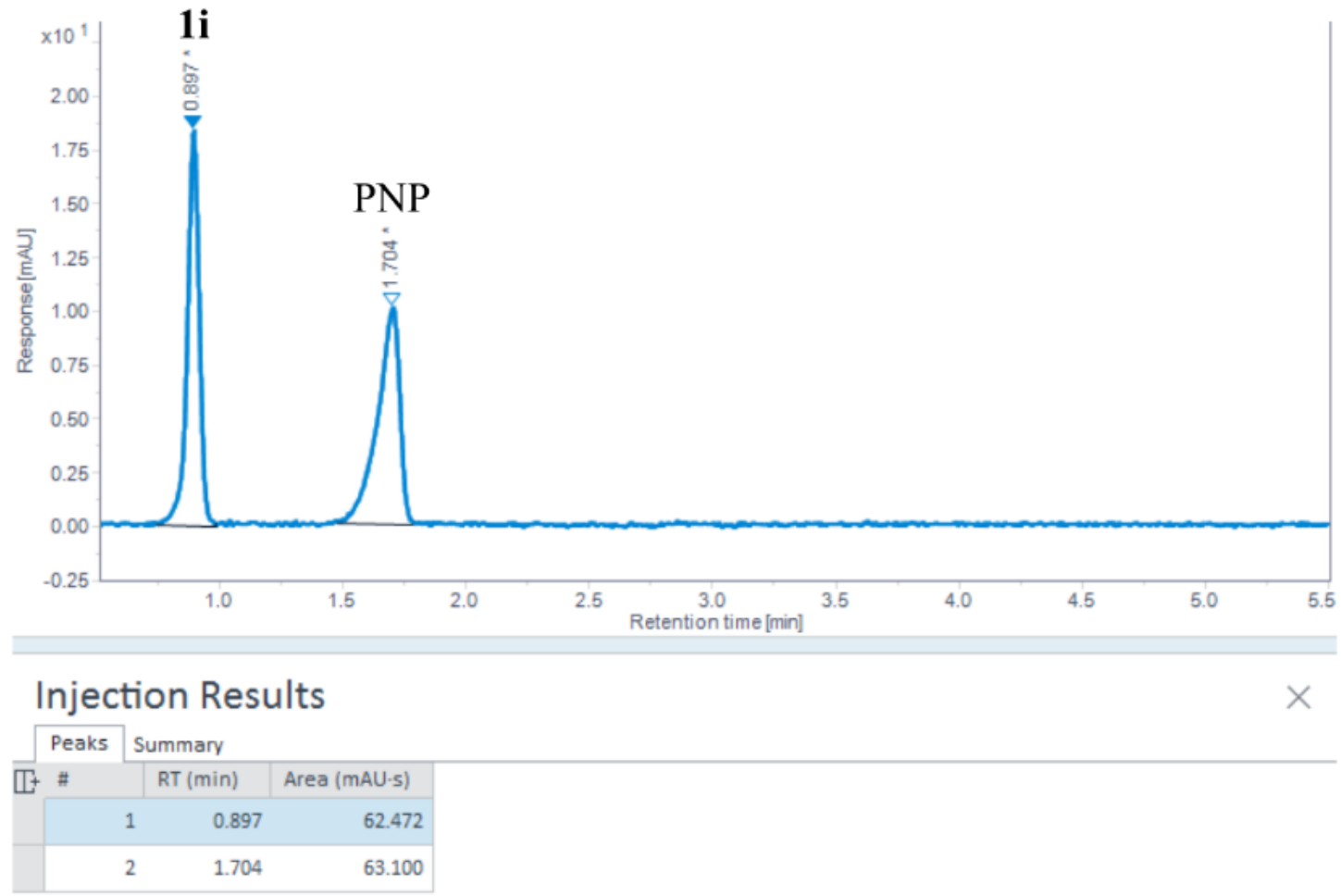

Compound	Peak area	Extinction coefficient / mM^{-1} cm^{-1}	Concentration / uM	Ratio
**1i**	62.5	290	215.5	1
PNP	63.1	293	215.4	0.9997

Supplementary Fig. 8: Molar ratio between product formed and PNP released. HPLC chromatogram for the reaction of **1** and **i** catalysed by ASB1.3. Peak areas for product **1i** and byproduct PNP are shown, and their molar ratio is calculated in the table. Reaction was performed using 200 µM **1**, 400 µM **i** and 5 µM ASB1.3 in PBS pH 7.4 with 5% MeCN for 3 hours.

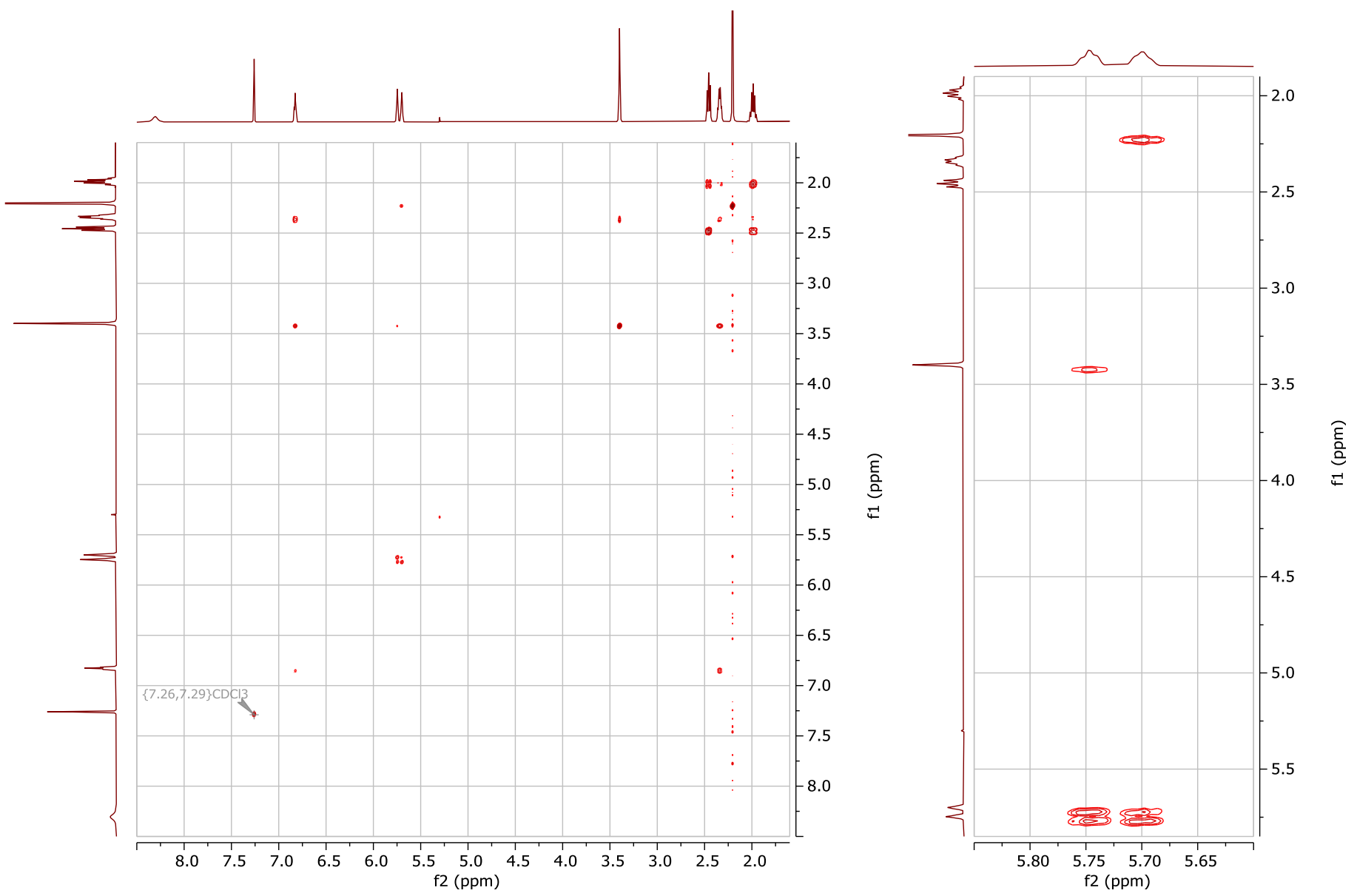

Supplementary Fig. 9: ^{1}H-^{1}H COSY (400 MHz, $CDCl_3$) of product 1v. Expansion (right) shows correlation peaks of pyrrole ring protons.

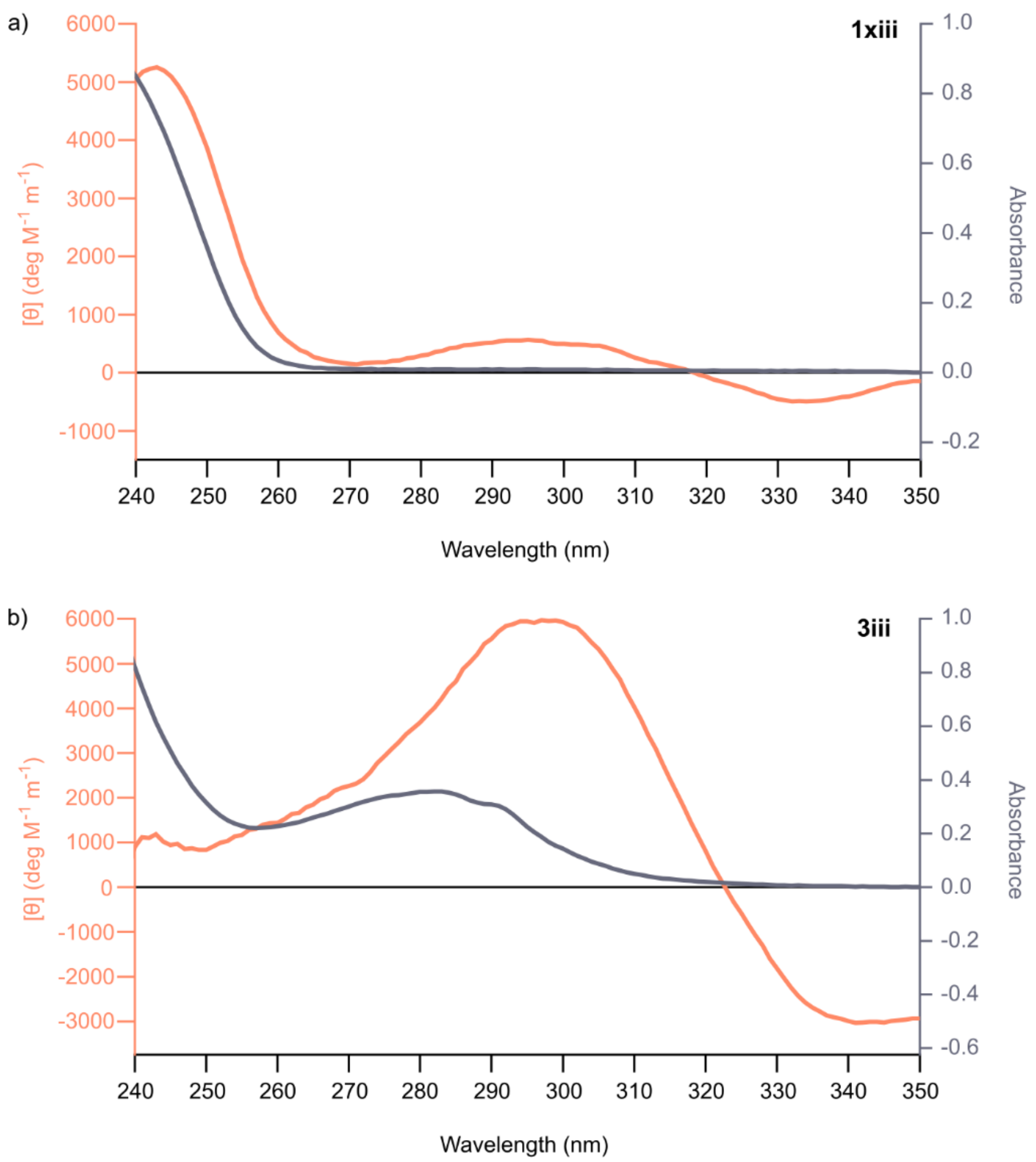

Supplementary Fig. 10: Absorbance and CD spectra of products 1xiii and 3iii. a) Absorbance (grey) and CD (orange) spectra of isolated product **1xiii**, measured at 800 µM, 52:48 *e.r.* in DCM. **b)** Absorbance (grey) and CD (orange) spectra of isolated product **3iii**, measured at 280 µM, 59:41 *e.r.* in DCM.

Supplementary Tables

Supplementary Table 1: Directed evolution of ASB1.3.

Round	Description	Clones screened	Beneficial mutations	Best variant
1	**Saturation mutagenesis** using libraries selected from rounds 7 and 9 of BH_{MeHis}1.8 evolution[1]<br>BH_{MeHis}1.6 scaffold: E26, M27, E100, L122, N123, D125<br>BH_{MeHis}1.7 scaffold: A20, R50, Y56, L68, L87, M94, A95, G99, L101, Y102, T126, A129, F132, T146, K155, E207	1936	E26Q, L68V, L68S, E100D, Y102A, Y102E, K155Q, E207V	**ASB1.1** = ASB1.0 L68V E100D = BH_{MeHis}1.6 L68V E100D
2	**Saturation mutagenesis** of L10, I14, I22, E26, W42, F45, F49, F53, Y61, V68, Q88, G91, A95, L101, Y102, L122, W124, F132, L136, K155	1144	L68M, K155A, K155E, K155F, K155P, K155MeHis	**ASB1.2** = ASB1.1 V68M K155F
3	**Saturation mutagenesis** of S9, G11, S15, A19, D46, K47, R50, E51, A64, R65, V71, M72, N123, N128, A129, I138, S148, A154, P156, N181, K184	1144	G11A, G11S, E51MeHis, E51P, E51S, V71I, A154MeHis	**ASB1.3** = ASB1.2 G11S E51P

Supplementary Table 2: Directed evolution of ASA1.5.

Round	Description	Clones screened	Beneficial mutations	Best variant
1	**Saturation mutagenesis** using libraries selected from rounds 7 and 9 of $BH_{MeHis}1.8$ evolution[1] $BH_{MeHis}1.6$ scaffold: E26, M27, E100, L122, N123, D125 $BH_{MeHis}1.7$ scaffold: A20, R50, Y56, L68, L87, M94, A95, G99, L101, Y102, T126, A129, F132, T146, K155, E207	1936	R50MeHis, F132W	**ASA1.1** = ASA1.0 F132W = $BH_{MeHis}1.7$ F132W
2	**Saturation mutagenesis** of L10, I14, I22, E26, R39, W42, F45, F49, R50, F53, Y61, F66, L68, L87, Q88, G91, G100, N123, W124, L136	1144	F45R, R50G, R50K R50Q, F53R, L68G, L68M, L68T, L68V	**ASA1.2** = ASA1.1 R50Q L68T
3	**Saturation mutagenesis** of S9, G11, S15, A19, R39, F45, D46, K47, E51, F53, A64, M72, A95, G100, N128, A129, L133, A154, K155, N181, K184	1144	G11K, K155F, N181W	**ASA1.3** = ASA1.2 K155F N181W
4	**Saturation mutagenesis** of G11, E26, D46, E51, S54, A57, Y61, V71, Q88, V92, Y102, I121, L122, N123, S148, P156, I160, L161, Y177, D180, P182, V183, K184, D185	1408	G11R, Y102S, I121A, I160G, I160S, I160V, P182C, P182G	**ASA1.4** = ASA1.3 G11R I121A I160V P182C
5	**Saturation mutagenesis** of L10, T12, I14, A19, V24, F45, F49, F53, A64, L87, G91, Y102, V120, L122, N123, W124, D125, N128, A129, T146, A154, L161, L192, E207	1408	V24G, V24Q, N123D, A154G, E207S	**ASA1.5** = ASA1.4 V24G N123D E207S

Supplementary Table 3: Reaction conditions for the substrate scope shown in Fig. 4. Reactions were performed in triplicate using 400 µM electrophile and 800 µM nucleophile in PBS pH 7.4 with 5% v/v MeCN.

Product	Electrophile	Nucleophile	Enzyme (mol%)	Time / hours	Mean % conversion ± s.d.	Mean *e.r.*
**1i**	**1**	**i**	ASB1.3 (1.25)	3	99.7 ± 0.1	94:6
**1ii**	**1**	**ii**	ASB1.1 (0.5)	3	>99.9 ± 0.0	85:15
**1xiii**	**1**	**xiii**	ASA1.2 (0.5)	1	99.9 ± 0.0	94:6
**1xiv**	**1**	**xiv**	ASB1.3 (2.5)	3	>99.9 ± 0.0	95:5
**1xi**	**1**	**xi**	ASA1.4 (2.5)	2	>99.9 ± 0.0	58:42
**1v**	**1**	**v**	ASA1.5 (0.5)	20	99 ± 0.1	N/A
**1iii**	**1**	**iii**	ASB1.3 (2.5)	2	90 ± 6.0	N/A
**1viii**	**1**	**viii**	ASA1.5 (2.5)	4	99.6 ± 0.04	N/A
**2ii**	**2**	**ii**	ASA1.5 (5)	6	93 ± 4.9	94:6
**2xiii**	**2**	**xiii**	ASA1.5 (5)	20	>99.9 ± 0.0	98:2
**2xii**	**2**	**xii**	ASA1.2 (5)	7	99 ± 0.2	66:34
**3iii**	*rac*-**3**	**iii**	ASB1.3 (5)	24	93 ± 1.8	60:40
**3iii**	(*R*)-**3**	**iii**	ASB1.3 (5)	24	>99.9 ± 0.0	98:2
**3iii**	(*S*)-**3**	**iii**	ASB1.3 (5)	24	53 ± 1.0	14:86
**3vii**	*rac*-**3**	**vii**	ASB1.3 (2.5)	2	98 ± 1.3	54:46
**3vii**	(*R*)-**3**	**vii**	ASB1.3 (2.5)	2	95 ± 0.4	89:11
**3vii**	(*S*)-**3**	**vii**	ASB1.3 (2.5)	2	93 ± 0.5	17:83

Supplementary Table 4: Reverse phase UPLC analysis methods. All methods used a flow rate of 1 mL min^{-1}. For products **1ii** and **1xi** the relevant normal phase methods reported in Supplementary Table 6 were used.

Product	Electrophile	Nucleophile	Mobile phase (% MeCN in H_2O)	Run time / min
**1i**	**1 or S4**	**i**	6.5% (0-3.5 min)<br>40% (3.51-5 min)<br>6.5% (5.01-6.5 min)	6.5
**1iii**	**1**	**iii**	25%	8
**1v**<br>**1viii**<br>**1xiii**<br>**1xiv**<br>**2xii**<br>**2xiii**<br>**3vii**	**1**<br>**1**<br>**1**<br>**1**<br>**2**<br>**2**<br>**3**	**v**<br>**viii**<br>**xiii**<br>**xiv**<br>**xii**<br>**xiii**<br>**vii**	5% (0-2 min)<br>5-60% (2-9 min)<br>5% (9.01-10 min)	10
**2ii**	**2**	**ii**	0% (0-2 min)<br>6.5% (2.01-8 min)<br>45% (8.01-9.5 min)<br>0% (9.51-11 min)	11
**3iii**	**3**	**iii**	25%	15

Supplementary Table 5: Extinction coefficients of electrophiles and products.

Compound	Extinction coefficient / mM^{-1} cm^{-1}	Measurement wavelength / nm
**1**	565	230
**1i**	290	230
**1ii**	269	230
**1iii**	447	230
**1v**	679	230
**1viii**	904	230
**1xi**	295	230
**1xiii**	85	230
**1xiv**	165	230
**2**	988	200
**2ii**	533	200
**2xii**	1047	200
**2xiii**	403	200
**3**	551	230
**3iii**	408	230
**3vii**	419	230
**S4**	517	230
PNP	293	230

Supplementary Table 6: Normal phase chiral HPLC analysis methods.

Compound	Column	Flow / mL min^{-1}	Mobile phase (% iPrOH in hexane)	Run time / min
**1i**	IG-3	1	5	20
**1ii**	IG-3	1	7	13
**1xi**	IG-3	1	7	8
**1xiii**	IG-3	1	5	10
**1xiv**	IG-3	1	5	8
**2xii**	IA-3	0.8	1	10
**2xiii**	IG-3	0.5	5	9
**3**	IA-3	0.8	1	5.5
**3iii**	IA-3	0.8	1	14
**3vii**	IG-3	0.5	1	40

Supplementary Table 7: Data collection and refinement statistics. Values in parentheses are for the highest resolution shell. R-free was calculated using ~5% of the data separate from the rest.

	**ASB1.3 (Lys39Ala)**
PDB accession number	9R2D
Wavelength (Å)	0.9763
Resolution range	37.43 - 1.689 (1.73 - 1.69)
Space group	C 2 2 21
Unit cell (a, b, c in Å; α, β, γ in °)	70.707, 142.475, 119.311; 90, 90, 90
Total reflections	905811 (47056)
Unique reflections	67749 (4796)
Multiplicity	13.4 (9.8)
Completeness (%)	98.75 (83.06)
Mean I/sigma(I)	11.35 (0.30)
Wilson B-factor	32.53
R-merge	0.09742 (2.861)
R-meas	0.1012 (3.019)
R-pim	0.02733 (0.9492)
$CC_{1/2}$	0.999 (0.361)
CC*	1 (0.728)
Reflections used in refinement	66898 (3987)
Reflections used for R-free	1908 (121)
R-work	0.1850 (0.3782)
R-free	0.2200 (0.4125)
Number of non-hydrogen atoms	4063
macromolecules	3738
ligands	58
solvent	267
Protein residues	458
RMS(bonds)	0.012
RMS(angles)	1.19
Ramachandran favored (%)	98.44
Ramachandran allowed (%)	1.33
Ramachandran outliers (%)	0.22
Rotamer outliers (%)	1.51
Clashscore	1.84
Average B-factor	42.23
macromolecules	42.11
ligands	45.31
solvent	43.18

Supplementary Table 8: Primers used in this study.

**Name**	**Primer sequence**
BH_{MeHis}1.6_NMH23_R	AATTTTATAAGCGCCTTCAACG
BH_{MeHis}1.6_NMH23A_F	GGCGCTTATAAAATT**GCG**GTGAAAGAGATGGAGGAAG
ASB1.3_K39_R	CGGGTTCAGCGGATA
ASB1.3_K39A_F	TATCCGCTGAACCCG**GCA**ACCCTGTGGGACG
ASB1.3_W124_R	ATTCAGGATCACGCCA
ASB1.3_W124A_F	GGCGTGATCCTGAAT**GCG**GATACCGAGAATGCCAC
ASB1.3_F132_R	TGCCGTGGCATTCT
ASB1.3_F132A_F	GAGAATGCCACGGCA**GCC**CTGGACGCACTGG

Supplementary Table 9: Primers used for library generation and shuffling. For round 1 libraries, see primers listed for rounds 7 and 9 in [1].

Name	Primer sequence
**Flanking primers**	
NDE_F	catgcatCATATGATTCGTGCGGTA
XHO_R	atgcatgcCTCGAGAGAGCCCTG
**Round 1 shuffling**	
NMH23A_F	GGCGCTTATAAAATT**GCG**GTGAAAGAGATGGAGGAAG
NMH23_R	AATTTTATAAGCGCCTTCAACG
R50NMR_F	TTGATAAACTGTTT**TAG**GAAGCGTTCTCTAACTATGC
R50_R	AAACAGTTTATCAAATTCGTCCC
L68V_F	CCGGCGCGTGATATC**GTG**GAAGAAGTAATGCGTAAACTGG
L68_R	GATATCACGCGCCG
E100D_F	GCGCGACGCTACGGC**GAT**CTGTACCCGGAAGTGG
E100_R	GCCGTAGCGTCG
E100D_Y102A_F	GCGCGACGCTACGGC**GAC**CTG**GCC**CCGGAAGTGGTGGA
E100D_Y102E_F	GCGCGACGCTACGGC**GAC**CTG**GAG**CCGGAAGTGGTGGA
Y102A_F	CGCTACGGCGAGCTG**GCC**CCGGAAGTGGTGGA
Y102E_F	CGCTACGGCGAGCTG**GAA**CCGGAAGTGGTGGA
Y102_R	CAGCTCGCCGTAG
N123NMH_F	GTTGGCGTGATCCTG**TAG**TGGGATACCGAGAATGC
N123D_F	GTTGGCGTGATCCTG**GAT**TGGGATACCGAGAATGC
N123_R	CAGGATCACGCCAAC
F132W_F	GAGAATGCCACGGCA**TGG**CTGGACGCACTGG
F132_R	TGCCGTGGCATTCT
E207M_F	AAAGGTGAGAAACGT**ATG**GAATTCTGGGATAAGGCG
E207V_F	AAAGGTGAGAAACGT**GTA**GAATTCTGGGATAAGGCG
E207_R	ACGTTTCTCACCTTTACG
**Round 2 libraries**	
AB_L10_R	GCTATCAAAGAATACCGCA
AB_L10_F	GTATTCTTTGATAGC**NNK**GGTACTCTGATTAGCGTTG
AB_I14_R	CAGAGTACCCAGGCTAT
AB_I14_F	AGCCTGGGTACTCTG**NNK**AGCGTTGAAGGCG
AB_I22_R	TTTATAAGCGCCTTCAACG
AB_I22_F	GAAGGCGCTTATAAA**NNK**TAGGTGAAAGAGATGGAGG
AB_E26_R	TTTCACCTAAATTTTATAAGCGC
AB_E26_F	AAAATTTAGGTGAAA**NNK**ATGGAGGAAGTGCTGG
AB_R39_R	CGGGTTCAGCGGATA
AB_R39_F	TATCCGCTGAACCCG**NNK**ACCCTGTGGGACG
B_W42_R	CAGGGTTTTCGGGTTC
B_W42_F	AACCCGAAAACCCTG**NNK**GACGAATTTGATAAACTGTTTCG
A_W42_R	CAGGGTTCGCGG
A_W42_F	AACCCGCGAACCCTG**NNK**GACGAATTTGATAAACTGTTTCG
AB_F45_R	TTCGTCCCACAGGG
AB_F45_F	ACCCTGTGGGACGAA**NNK**GATAAACTGTTTCGCGAAG
AB_F49_R	CAGTTTATCAAATTCGTCCCA
AB_F49_F	GAATTTGATAAACTG**NNK**CGCGAAGCGTTCTC
AB_R50_R	AAACAGTTTATCAAATTCGTCCC
AB_R50_F	TTTGATAAACTGTTT**NNK**GAAGCGTTCTCTAACTATGC
AB_F53_R	CGCTTCGCGAAACA
AB_F53_F	CTGTTTCGCGAAGCG**NNK**TCTAACTATGCGGGCAA
AB_Y61_R	CGGTTTGCCCGC
AB_Y61_F	TATGCGGGCAAACCG**NNK**CGTCCGGCGC
AB_F66_R	ACGCGCCGGAC
A_F66_F	TATCGTCCGGCGCGT**NNK**ATCCTGGAAGAAGTAATGC
B_V68_R	GATATCACGCGCCG
B_V68_F	CCGGCGCGTGATATC**NNK**GAAGAAGTAATGCGTAAACTG
A_L68_R	GATAAAACGCGCCGG
A_L68_F	CCGGCGCGTTTTATC**NNK**GAAGAAGTAATGCGTAAACTG
AB_L87_R	GTTTCCAGGGTATTTGAAACC
AB_L87_F	AAATACCCTGGAAAC**NNK**CAGGAAATCGGCGTG

AB_Q88_R	CAAGTTTCCAGGGTATTTGAAA
AB_Q88_F	TACCCTGGAAACTTG**NNK**GAAATCGGCGTGCG
AB_G91_R	GATTTCCTGCAAGTTTCCA
AB_G91_F	AACTTGCAGGAAATC**NNK**GTGCGTATGGCGC
AB_A95_R	CATACGCACGCCG
B_A95_F	ATCGGCGTGCGTATG**NNK**CGACGCTACGGC
AB_G100_R	GCCGTAGCGTCG
AB_G100_F	GCGCGACGCTACGGC**NNK**CTGTACCCGGAAGTG
B_L101_R	ATCGCCGTAGCGT
B_L101_F	CGACGCTACGGCGAT**NNK**TACCCGGAAGTGGTG
B_Y102_R	CAGATCGCCGTAGC
B_Y102_F	CGCTACGGCGATCTG**NNK**CCGGAAGTGGTGGA
AB_L122_R	GATCACGCCAACGTG
AB_L122_F	CACGTTGGCGTGATC**NNK**AATTGGGATACCGAGAATG
AB_N123_R	CAGGATCACGCCAAC
AB_N123_F	GTTGGCGTGATCCTG**NNK**TGGGATACCGAGAATGC
AB_W124_R	ATTCAGGATCACGCCA
AB_W124_F	GGCGTGATCCTGAAT**NNK**GATACCGAGAATGCCAC
AB_F132_R	TGCCGTGGCATTCT
AB_F132_F	GAGAATGCCACGGCA**NNK**CTGGACGCACTGG
B_L136_R	TGCGTCCAGGAATGC
B_L136_F	GCATTCCTGGACGCA**NNK**GGCATCAAAGACCTGTT
A_L136_R	TGCGTCCAGCCAT
A_L136_F	GCATGGCTGGACGCA**NNK**GGCATCAAAGACCTGTT
AB_K155_R	AGCGAAACCAGCTTCT
AB_K155_F	GAAGCTGGTTTCGCT**NNK**CCGCACCCACG
**Round 2 shuffling**	
A_R50G_F	TTTGATAAACTGTTT**GGC**GAAGCGTTCTCTAACTATGC
A_R50Q_F	TTTGATAAACTGTTT**CAG**GAAGCGTTCTCTAACTATGC
B_L68M_F	CCGGCGCGTGATATC**ATG**GAAGAAGTAATGCGTAAACTGG
**Round 3 libraries**	
3AB_S9_R	ATCAAAGAATACCGCACG
3AB_S9_F	GCGGTATTCTTTGAT**NNK**CTGGGTACTCTGATTAGCG
3AB_G11_R	CAGGCTATCAAAGAATACCG
3AB_G11_F	TTCTTTGATAGCCTG**NNK**ACTCTGATTAGCGTTGAAG
3AB_S15_R	AATCAGAGTACCCAGGC
3AB_S15_F	CTGGGTACTCTGATT**NNK**GTTGAAGGCGCTTATAAAATTTAG
3AB_A19_R	GCCTTCAACGCTAATCAG
3AB_A19_F	ATTAGCGTTGAAGGC**NNK**TATAAAATTTAGGTGAAAGAGATGGAG
3A_F45_F	ACCCTGTGGGACGAA**NNK**GATAAACTGTTTCAGGAAGCG
3AB_D46_R	AAATTCGTCCCACAGGG
3A_D46_F	CTGTGGGACGAATTT**NNK**AAACTGTTTCAGGAAGCG
3B_D46_F	CTGTGGGACGAATTT**NNK**AAACTGTTTCGCGAAGC
3AB_K47_R	ATCAAATTCGTCCCACAG
3A_K47_F	TGGGACGAATTTGAT**NNK**CTGTTTCAGGAAGCGTTC
3B_K47_F	TGGGACGAATTTGAT**NNK**CTGTTTCGCGAAGCG
3A_E51_R	CTGAAACAGTTTATCAAATTCGTC
3A_E51_F	GATAAACTGTTTCAG**NNK**GCGTTCTCTAACTATGCG
3B_E51_R	GCGAAACAGTTTATCAAATTCG
3B_E51_F	GATAAACTGTTTCGC**NNK**GCGTTCTCTAACTATGCG
3A_F53_R	CGCTTCCTGAAACAGTTTAT
3A_F53_F	CTGTTTCAGGAAGCG**NNK**TCTAACTATGCGGGCAA
3AB_A64_R	CGGACGATACGGTTTG
3A_A64_F	AAACCGTATCGTCCG**NNK**CGTTTTATCACGGAAGAAGT
3B_A64_F	AAACCGTATCGTCCG**NNK**CGTGATATCATGGAAGAAGTAATG
3B_R65_R	CGCCGGACGATAC
3B_R65_F	CCGTATCGTCCGGCG**NNK**GATATCATGGAAGAAGTAATGCG
3B_V71_R	TTCTTCCATGATATCACGC
3B_V71_F	GATATCATGGAAGAA**NNK**ATGCGTAAACTGGCG
3A_M72_R	TACTTCTTCCGTGATAAAACG
3A_M72_F	ATCACGGAAGAAGTA**NNK**CGTAAACTGGCGGAAAA
3B_M72_R	TACTTCTTCCATGATATCACGC
3B_M72_F	ATCATGGAAGAAGTA**NNK**CGTAAACTGGCGGAAAA
3A_G100_F	GCGCGACGCTACGGC**NNK**CTGTACCCGGAAGTGG

3AB_N128_R	CTCGGTATCCCAATTCAG
3A_N128_F	AATTGGGATACCGAG**NNK**GCCACGGCATGG
3B_N128_F	AATTGGGATACCGAG**NNK**GCCACGGCATTCC
3AB_A129_R	ATTCTCGGTATCCCAATTCA
3A_A129_F	TGGGATACCGAGAAT**NNK**ACGGCATGGCTGG
3B_A129_F	TGGGATACCGAGAAT**NNK**ACGGCATTCCTGGA
3A_L133_R	CCATGCCGTGGC
3A_L133_F	AATGCCACGGCATGG**NNK**GACGCACTGGGC
3B_I138_R	GCCCAGTGCGTC
3B_I138_F	CTGGACGCACTGGGC**NNK**AAAGACCTGTTCGATTCCA
3B_S148_R	CGTGGTGATGGAATCG
3B_S148_F	GATTCCATCACCACG**NNK**GAAGAAGCTGGTTTCGC
3AB_A154_R	GAAACCAGCTTCTTCAGAC
3A_A154_F	GAAGAAGCTGGTTTC**NNK**AAACCGCACCCAC
3B_A154_F	GAAGAAGCTGGTTTC**NNK**TTTCCGCACCCACG
3A_K155_R	AGCGAAACCAGCTTCTT
3B_P156_R	AAAAGCGAAACCAGCTTC
3B_P156_F	GCTGGTTTCGCTTTT**NNK**CACCCACGCATCCT
3AB_N181_R	GTCACCAACGTACACTG
3AB_N181_F	GTGTACGTTGGTGAC**NNK**CCGGTCAAAGACGC
3AB_K184_R	GACCGGGTTGTCAC
3AB_K184_F	GGTGACAACCCGGTC**NNK**GACGCGGGTGGT
**Round 3 shuffling**	
3A_G11K_F	TTCTTTGATAGCCTG**AAG**ACTCTGATTAGCGTTGAAG
3A_K155F_F	GAAGCTGGTTTCGCT**TTC**CCGCACCCACG
3A_N181W_F	GTGTACGTTGGTGAC**TGG**CCGGTCAAAGACGC
3B_G11S_F	TTCTTTGATAGCCTG**AGT**ACTCTGATTAGCGTTGAAG
3B_G11A_F	TTCTTTGATAGCCTG**GCT**ACTCTGATTAGCGTTGAAG
3B_E51S_F	GATAAACTGTTTCGC**TCA**GCGTTCTCTAACTATGCG
3B_E51P_F	GATAAACTGTTTCGC**CCA**GCGTTCTCTAACTATGCG
3B_A154H_F	GAAGAAGCTGGTTTC**CAT**TTCCGCACCCACG
**Round 4 libraries**	
4A_S54_R	GAACGCTTCCTGAAACAG
4A_S54_F	TTTCAGGAAGCGTTC**NNK**AACTATGCGGGCAAAC
4A_A57_R	ATAGTTAGAGAACGCTTCCT
4A_A57_F	GCGTTCTCTAACTAT**NNK**GGCAAACCGTATCGTC
4A_V71_R	TTCTTCCGTGATAAAACGC
4A_V71_F	TTTATCACGGAAGAA**NNK**ATGCGTAAACTGGCG
4A_V92_R	GCCGATTTCCTGCAAG
4A_V92_F	TTGCAGGAAATCGGC**NNK**CGTATGGCGCGAC
4A_Y102_R	CAGCCCGCCGTA
4A_Y102_F	CGCTACGGCGGGCTG**NNK**CCGGAAGTGGTGGAA
4A_I121_R	CACGCCAACGTGATATTTA
4A_I121_F	TATCACGTTGGCGTG**NNK**CTGAATTGGGATACCGAGA
4A_P156_R	GAAAGCGAAACCAGCTTC
4A_P156_F	GCTGGTTTCGCTTTC**NNK**CACCCACGCATCCT
4A_I160_R	GCGTGGGTGCG
4A_I160_F	TTCCCGCACCCACGC**NNK**CTCGAACTGGCTCTGAA
4A_L161_R	GATGCGTGGGTGC
4A_L161_F	CCGCACCCACGCATC**NNK**GAACTGGCTCTGAAGAAAG
4A_Y177_R	CACTGCTTTCTCGCCTTTA
4A_Y177_F	GGCGAGAAAGCAGTG**NNK**GTTGGTGACTGGCC
4A_D180_R	ACCAACGTACACTGCTTT
4A_D180_F	GCAGTGTACGTTGGT**NNK**TGGCCGGTCAAAGA
4A_P182_R	CCAGTCACCAACGTACA
4A_P182_F	TACGTTGGTGACTGG**NNK**GTCAAAGACGCGGG
4A_V183_R	CGGCCAGTCACCAA
4A_V183_F	GTTGGTGACTGGCCG**NNK**AAAGACGCGGGTGG
4A_K184_R	GACCGGCCAGTCA
4A_K184_F	GGTGACTGGCCGGTC**NNK**GACGCGGGTGGTT
4A_D185_R	TTTGACCGGCCAGT
4A_D185_F	GACTGGCCGGTCAAA**NNK**GCGGGTGGTTCTAAGAA
**Round 4 shuffling**	
4A_I121A_F	TATCACGTTGGCGTG**GCT**CTGAATTGGGATACCGAG

4A_I160G_F	TTCCCGCACCCACGC**GGT**CTCGAACTGGCTCTGA
4A_I160S_F	TTCCCGCACCCACGC**TCG**CTCGAACTGGCTCTGA
4A_I160V_F	TTCCCGCACCCACGC**GTG**CTCGAACTGGCTCTGA
4A_P182C_F	TACGTTGGTGACTGG**TGT**GTCAAAGACGCGGG
**Round 5 libraries**	
5A_L10_F	GTATTCTTTGATAGC**NNK**CGGACTCTGATTAGCG
5A_T12_R	CCGCAGGCTATCAAAG
5A_T12_F	TTTGATAGCCTGCGG**NNK**CTGATTAGCGTTGAAGGC
5A_I14_R	CAGAGTCCGCAGG
5A_I14_F	AGCCTGCGGACTCTG**NNK**AGCGTTGAAGGCG
5A_V24_R	CTAAATTTTATAAGCGCCTTCAAC
5A_V24_F	GCTTATAAAATTTAG**NNK**AAAGAGATGGAGGAAGTG
5A_F49_F	GAATTTGATAAACTG**NNK**CAGGAAGCGTTCTCTAAC
5A_V120_R	GCCAACGTGATATTTACCTTT
5A_V120_F	AAATATCACGTTGGC**NNK**GCTCTGAATTGGGATACC
5A_L122_R	AGCCACGCCAAC
5A_L122_F	CACGTTGGCGTGGCT**NNK**AATTGGGATACCGAGAATG
5A_N123_R	CAGAGCCACGCCA
5A_N123_F	GTTGGCGTGGCTCTG**NNK**TGGGATACCGAGAATGC
5A_W124_R	ATTCAGAGCCACGC
5A_W124_F	GGCGTGGCTCTGAAT**NNK**GATACCGAGAATGCCAC
5A_D125_R	CCAATTCAGAGCCACG
5A_D125_F	GTGGCTCTGAATTGG**NNK**ACCGAGAATGCCAC
5A_T146_R	GATGGAATCGAACAGGTC
5A_T146_F	CTGTTCGATTCCATC**NNK**ACGTCTGAAGAAGCTGG
5A_A154_F	GAAGAAGCTGGTTTC**NNK**TTCCCGCACCCAC
5A_L161_R	CACGCGTGGGTG
5A_L161_F	CCGCACCCACGCGTG**NNK**GAACTGGCTCTGAAGAAAG
5A_L192_R	GTTCTTAGAACCACCCG
5A_L192_F	GGTGGTTCTAAGAAC**NNK**GGTATGACTAGCATCCTG
5A_E207_R	ACGTTTCTCACCTTTACGA
5A_E207_F	AAAGGTGAGAAACGT**NNK**TTCTGGGATAAGGCGG
**Round 5 shuffling**	
5A_V24G_F	GCTTATAAAATTTAG**GGG**AAAGAGATGGAGGAAGTG
5A_V24Q_F	GCTTATAAAATTTAG**CAG**AAAGAGATGGAGGAAGTG
5A_N123D_F	GTTGGCGTGGCTCTG**GAT**TGGGATACCGAGAATGC
5A_A154G_F	GAAGAAGCTGGTTTC**GGT**TTCCCGCACCCA
5A_E207S_F	AAAGGTGAGAAACGT**TCA**TTCTGGGATAAGGCGG

Supplementary Methods

Chemical Synthesis

Preparation of substrates

2-(Hydroxymethyl)cyclohex-2-en-1-one (S1). A round bottom flask was charged with cyclohexenone (2.4 g, 25 mmol), THF (20 mL), 37% w/v aqueous formaldehyde (3.94 mL, 50 mmol) and 4-dimethylaminopyridine (305.4 mg, 2.5 mmol), which was then stirred at room temperature for 8 days. The reaction mixture was acidified with 1 M HCl to pH 3, then H_2O (20 mL) was added and the product extracted with DCM (3 x 20 mL). The combined organic extracts were washed with brine (2 x 30 mL), dried over $MgSO_4$, filtered, and concentrated under reduced pressure. The crude product was purified by silica column chromatography (3:1 to 1:1 cyclohexane:EtOAc) to give the product as a brown oil. Yield 1.0754 g (34.1%). The spectral data are consistent with literature values[2]. ^{1}H NMR (400 MHz, $CDCl_3$) δ 6.93 (t, J = 4.1 Hz, 1H), 4.24 (d, J = 1.3 Hz, 2H), 2.47 – 2.37 (m, 5H), 2.01 (p, J = 6.2 Hz, 2H). ^{13}C NMR (101 MHz, $CDCl_3$) δ 200.8, 147.1, 138.4, 62.3, 38.4, 25.8, 22.9.

2-((4-Nitrophenoxy)methyl)cyclohex-2-en-1-one (1). A round bottom flask was charged with 4-nitrophenol (181.6 mg, 1.31 mmol), triphenylphosphine (343.2 mg, 1.31 mmol) and THF (12 mL), which was then stirred in an ice bath under N_2. **S1** (150 mg, 1.19 mmol) was separately dissolved in THF (3 mL), then added dropwise to the reaction mixture on ice. Diisopropyl azodicarboxylate (264.8 mg, 1.31 mmol) was added dropwise to the reaction mixture on ice. The reaction mixture was stirred on ice for 30 min, then stirred at room temperature overnight. Diethyl ether (30 mL) was added to the reaction mixture, which was then washed with 0.1M NaOH (3 x 15 mL) and brine (2 x 15 mL), dried over $MgSO_4$, filtered, and concentrated under reduced pressure. The crude product was purified by silica column chromatography (column 1: neat DCM, column 2: 5:1 to 4:1 cyclohexane:EtOAc) to give the product as a white powder. Yield 117.0 mg (39.8%). ^{1}H NMR (400 MHz, $CDCl_3$) δ 8.24 – 8.17 (m, 2H), 7.14 (t, J = 4.2 Hz, 1H), 7.03 – 6.96 (m, 2H), 4.79 (q, J = 1.8 Hz, 2H), 2.55 – 2.44 (m, 4H), 2.07 (p, J = 6.2 Hz, 2H). ^{13}C NMR (101 MHz, $CDCl_3$) δ 198.3, 163.6, 148.2, 141.8, 134.1, 126.1, 114.8, 65.4, 38.2, 25.9, 22.8. HRMS (APCI$^+$): Calc.d for $C_{13}H_{14}NO_4^+$ ($[M+H]^+$) 248.0917, found 248.0921.

Methyl 2-(bromomethyl)acrylate (S2). A round bottom flask was charged with methyl 2-(hydroxymethyl)acrylate (348 mg, 3 mmol) and DCM (15 mL), which was then stirred in an ice bath under N_2. DMF (231 uL, 3 mmol) then PBr_3 (315 uL, 3.3 mmol) were added dropwise, and the reaction was stirred at room temperature for 1 hour. The reaction was quenched with

H_2O (12 mL) on ice, and the product was extracted with DCM (3 x 20 mL). The combined organic extracts were concentrated under reduced pressure. The crude product was purified by silica column chromatography (neat DCM) to give the product as a brown oil. Yield 470.2 mg (87.6%). The spectral data are consistent with literature values[3]. ^1H NMR (400 MHz, $CDCl_3$) δ 6.33 (d, J = 0.8 Hz, 1H), 5.96 (d, J = 0.9 Hz, 1H), 4.18 (d, J = 0.9 Hz, 2H), 3.81 (s, 3H). ^{13}C NMR (101 MHz, $CDCl_3$) δ 165.5, 137.4, 129.4, 52.4, 29.4.

Methyl 2-((4-nitrophenoxy)methyl)acrylate (2). A round bottom flask was charged with K_2CO_3 (331.7 mg, 2.4 mmol) and 3 Å molecular sieves and placed under N_2. 4-Nitrophenol (170.5 mg, 1.2 mmol) dissolved in anhydrous DMF (4 mL) was added and the mixture stirred at room temperature for 5 min. **S2** (219.6 mg, 1.2 mmol) dissolved in anhydrous DMF (2 mL) was added and the mixture stirred at room temperature for 4 hours. The reaction was quenched with H_2O (25 mL), and the product was extracted with EtOAc (3 x 40 mL). The combined organic extracts were washed with brine (2 x 50 mL), dried over $MgSO_4$, filtered, and concentrated under reduced pressure. The crude product was purified by silica column chromatography (neat DCM) to give the product as a white powder. Yield 238.0 mg (81.8%). ^1H NMR (400 MHz, $CDCl_3$) δ 8.26 – 8.17 (m, 2H), 7.05 – 6.97 (m, 2H), 6.46 (q, J = 1.3 Hz, 1H), 6.01 (td, J = 1.8, 0.9 Hz, 1H), 4.85 (t, J = 1.6 Hz, 2H), 3.83 (s, 3H). ^{13}C NMR (101 MHz, $CDCl_3$) δ 165.7, 163.3, 142.0, 134.8, 127.5, 126.1, 114.9, 66.8, 52.3. HRMS ($APCI^+$): Calc.d for $C_{11}H_{12}NO_5^+$ ($[M+H]^+$) 238.0709, found 238.0705.

2-(1-Hydroxyethyl)cyclohex-2-en-1-one (S3). A round bottom flask was charged with cyclohexenone (3.00 g, 31.2 mmol), imidazole (212.5 mg, 3.12 mmol), THF (6.22 mL) and H_2O (6.22 mL), which was then stirred at room temperature under N_2. Acetaldehyde (13.75 g, 312 mmol) was added to the reaction mixture, which was then stirred at 45 °C for 10 days. The reaction mixture was acidified with 1 M HCl to pH 2, then the product was extracted with DCM (3 x 40 mL). The combined organic extracts were washed with 1 M $NaHCO_3$ (2 x 50 mL) and brine (2 x 50 mL), dried over $MgSO_4$, filtered, and concentrated under reduced pressure. The crude product was purified by silica column chromatography (4:1 to 2:1 cyclohexane:EtOAc) to give the product as a brown oil. Yield 1.1656 g (26.6%). The spectral data are consistent with literature values[4]. ^1H NMR (400 MHz, $CDCl_3$) δ 6.90 (t, J = 4.2 Hz, 1H), 4.62 – 4.51 (m, 1H), 3.01 (d, J = 5.6 Hz, 1H), 2.47 – 2.37 (m, 4H), 2.03 – 1.95 (m, 2H), 1.34 (d, J = 6.5 Hz, 3H). ^{13}C NMR (101 MHz, $CDCl_3$) δ 201.0, 145.1, 142.0, 66.8, 38.8, 25.8, 22.7, 21.8.

2-(1-(4-Nitrophenoxy)ethyl)cyclohex-2-en-1-one (3). A round bottom flask was charged with **S3** (140.2 mg, 1 mmol), THF (9 mL), 4-nitrophenol (208.7 mg, 1.5 mmol) and triphenylphosphine (498.4 mg, 1.9 mmol), which was then stirred in an ice bath under N_2. Diisopropyl azodicarboxylate (404.4 mg, 2 mmol) was separately dissolved in THF (1 mL), then added dropwise to the reaction mixture on ice. The reaction mixture was stirred on ice for 30 min, then stirred at room temperature overnight. The reaction was worked up as above for **1**, and the crude product was purified by silica column chromatography (column 1: neat DCM, column 2: 4:1 cyclohexane:EtOAc) to give the product as a white powder. Yield 131.0 mg (50.1%). ^{1}H NMR (400 MHz, $CDCl_3$) δ 8.20 – 8.09 (m, 2H), 6.99 (t, J = 4.2 Hz, 1H), 6.89 – 6.81 (m, 2H), 5.43 (q, J = 6.4 Hz, 1H), 2.49 (dd, J = 7.8, 5.7 Hz, 2H), 2.44 – 2.30 (m, 2H), 2.06 – 1.92 (m, 2H), 1.46 (d, J = 6.3 Hz, 3H). ^{13}C NMR (101 MHz, $CDCl_3$) δ 198.2, 162.7, 146.2, 141.6, 139.7, 126.0, 115.4, 70.4, 38.4, 25.8, 22.7, 22.0. HRMS (APCI$^+$): Calc.d for $C_{14}H_{16}NO_4^+$ ($[M+H]^+$) 262.1073, found 262.1074. The racemate was separated by Reach Separations (ChiralPak® 5 µm IG column, 250 x 21 mm) to give the enantiomers (*R*)-**3** (44.4 mg, 99.9:0.1 *e.r.*) and (*S*)-**3** (47.1 mg, 98.9:1.1 *e.r.*).

(6-Oxocyclohex-1-en-1-yl)methyl acetate (S4). A round bottom flask was charged with **S1** (138.9 mg, 1.10 mmol), acetic anhydride (138.9 µL, 1.47 mmol) and pyridine (694.5 µL, 8.62 mmol), which was then stirred at room temperature overnight. EtOAc (20 mL) was added to the reaction mixture, which was then washed with 1 M $NaHCO_3$ (2 × 10 mL), 10% w/v $CuSO_4$ (2 × 10 mL) and brine (10 mL), dried over $MgSO_4$, filtered, and concentrated under reduced pressure to give the product as a brown oil. Yield 126.8 mg (68.5%). The spectral data are consistent with literature values[5,6]. ^{1}H NMR (400 MHz, $CDCl_3$) δ 6.95 (t, J = 4.2 Hz, 1H), 4.67 (d, J = 1.5 Hz, 2H), 2.43 – 2.34 (m, 4H), 2.03 – 1.94 (m, 5H). ^{13}C NMR (101 MHz, $CDCl_3$) δ 198.0, 170.7, 148.7, 134.4, 61.4, 38.1, 25.8, 22.7, 20.9.

Methyl 2-(acetoxymethyl)acrylate (S5). A round bottom flask was charged with methyl 2-(hydroxymethyl)acrylate (1 g, 8.61 mmol), acetic anhydride (1.08 mL, 11.45 mmol) and pyridine (5.42 mL, 67.27 mmol), which was then stirred at room temperature overnight. The reaction was worked up as above to give the product as a brown oil. Yield 1.1696 g (85.9%). The spectral data are consistent with literature values[7]. ^{1}H NMR (400 MHz, $CDCl_3$) δ 6.36 (d, J = 1.1 Hz, 1H), 5.85 (d, J = 1.4 Hz, 1H), 4.80 (s, 2H), 3.79 (s, 3H), 2.10 (s, 3H). ^{13}C NMR (101 MHz, $CDCl_3$) δ 170.6, 165.8, 135.4, 127.7, 62.6, 52.2, 21.0.

1-(6-Oxocyclohex-1-en-1-yl)ethyl acetate (S6). A round bottom flask was charged with **S3** (200 mg, 1.43 mmol), acetic anhydride (179 µL, 1.90 mmol) and pyridine (897 µL, 11.13 mmol), which was then stirred at room temperature overnight. EtOAc (30 mL) was added to the reaction mixture, which was then washed with 1 M $NaHCO_3$ (2 × 10 mL), 10% w/v $CuSO_4$ (3 × 10 mL) and brine (2 x 10 mL), dried over $MgSO_4$, filtered, and concentrated under reduced pressure. The crude product was purified by silica column chromatography (4:1 cyclohexane:EtOAc) to give the product as a brown oil. Yield 197.8 mg (75.9%). ^{1}H NMR (400 MHz, $CDCl_3$) δ 6.92 (t, J = 4.2 Hz, 1H), 5.72 (q, J = 6.5 Hz, 1H), 2.46 – 2.36 (m, 4H), 2.05 (s, 3H), 2.01 – 1.93 (m, 2H), 1.33 (d, J = 6.5 Hz, 3H). ^{13}C NMR (101 MHz, $CDCl_3$) δ 197.6, 170.0, 144.6, 140.0, 67.4, 38.6, 25.8, 22.7, 21.4, 20.5. HRMS (ESI$^+$): Calc.d for $C_{10}H_{14}O_3Na^+$ ([M+Na]$^+$) 205.0835, found 205.0834.

Preparation of product standards

5-((6-Oxocyclohex-1-en-1-yl)methyl)furan-2(5*H*)-one (1i). Two conical flasks were each charged with PBS pH 7.4 (36.7 mL), DMSO (3 mL), **1** (50 mM in DMSO, 2 mL), **i** (50 mM in DMSO, 5 mL) and BH32.8[8] (150.3 µM in PBS pH 7.4, 3.33 mL), and incubated at 30 °C, 180 r.p.m. for 3 days. The reactions were pooled and the product was extracted with EtOAc (4 x 50 mL). The combined organic extracts were washed with 0.1 M NaOH (4 x 50 mL) and brine (2 x 50 mL), dried over $MgSO_4$, filtered, and concentrated under reduced pressure. The crude product was purified by silica column chromatography (column 1: 2:1 cyclohexane:EtOAc, column 2: 1:49 MeOH:DCM) to give the product as a brown oil. Yield 6.9 mg (17.9%). ^{1}H NMR (400 MHz, $CDCl_3$) δ 7.41 (dd, J = 5.7, 1.5 Hz, 1H), 6.93 (t, J = 4.2 Hz, 1H), 6.03 (dd, J = 5.7, 2.0 Hz, 1H), 5.19 (ddt, J = 6.4, 4.4, 1.8 Hz, 1H), 2.73 (dd, J = 14.0, 4.4 Hz, 1H), 2.55 (dd, J = 13.9, 6.8 Hz, 1H), 2.49 – 2.34 (m, 4H), 2.07 – 1.88 (m, 2H). ^{13}C NMR (101 MHz, $CDCl_3$) δ 199.4, 173.2, 156.4, 150.9, 133.0, 121.5, 81.8, 38.3, 33.2, 26.3, 23.0. HRMS (ESI$^+$): Calc.d for $C_{11}H_{13}O_3^+$ ([M+H]$^+$) 193.0858, found 193.0857.

3-Acetyl-3-((6-oxocyclohex-1-en-1-yl)methyl)dihydrofuran-2(3*H*)-one (1ii). A round bottom flask was charged with **S4** (90 mg, 0.535 mmol), THF (2 mL), H_2O (0.66 mL), and 1,4-diazabicyclo[2.2.2]octane (120.0 mg, 1.07 mmol), which was stirred at room temperature for 15 min. **ii** (75.4 mg, 0.589 mmol) was added and the reaction was stirred at room temperature for 5 days. EtOAc (60 mL) was added to the reaction mixture, which was then washed with brine (2 x 40 mL), dried over $MgSO_4$, filtered, and concentrated under reduced pressure. The crude product was purified by silica column chromatography (3:1 *n*-

hexane:EtOAc to neat EtOAc) to give the product as a brown oil. Yield 35.1 mg (27.8%). ^{1}H NMR (400 MHz, $CDCl_3$) δ 6.88 (t, J = 4.2 Hz, 1H), 4.21 (td, J = 8.7, 3.6 Hz, 1H), 4.10 (dt, J = 8.9, 7.1 Hz, 1H), 3.14 (d, J = 14.1 Hz, 1H), 2.69 (ddd, J = 13.2, 7.0, 3.6 Hz, 1H), 2.63 (d, J = 14.1 Hz, 1H), 2.46 – 2.33 (m, 7H), 2.07 – 1.91 (m, 3H). ^{13}C NMR (101 MHz, $CDCl_3$) δ 202.2, 199.1, 175.7, 150.3, 134.7, 66.5, 61.7, 38.3, 32.9, 29.4, 26.5, 26.1, 22.9. HRMS (ESI$^+$): Calc.d for $C_{13}H_{17}O_4^+$ ([M+H]$^+$) 237.1121, found 237.1116.

2-((2-Methyl-1*H*-indol-3-yl)methyl)cyclohex-2-en-1-one (1iii). A conical flask was charged with PBS pH 7.4 (38 mL), DMSO (3 mL), **S4** (100 mM in DMSO, 3 mL), **iii** (100 mM in DMSO, 6 mL) and BH32.8[8] (893.4 µM in PBS pH 7.4, 10 mL), and incubated at 30 °C, 180 r.p.m. for 4 days. The product was extracted with EtOAc (3 x 50 mL), and the combined organic extracts were washed with H_2O (3 x 50 mL) and brine (2 x 50 mL), dried over $MgSO_4$, filtered, and concentrated under reduced pressure. The crude product was purified by silica column chromatography (3:1 cyclohexane:EtOAc) to give the product as a brown oil. Yield 17.5 mg (24.4%). ^{1}H NMR (400 MHz, DMSO-d_6) δ 10.73 (s, 1H), 7.29 – 7.18 (m, 2H), 7.00 – 6.91 (m, 1H), 6.91 – 6.83 (m, 1H), 6.49 (t, J = 4.2 Hz, 1H), 3.44 (d, J = 1.9 Hz, 2H), 2.39 – 2.33 (m, 2H), 2.29 (s, 3H), 2.26 – 2.19 (m, 2H), 1.85 (p, J = 6.1 Hz, 2H). ^{13}C NMR (101 MHz, DMSO-d_6) δ 198.5, 145.4, 137.6, 135.2, 132.5, 128.3, 119.8, 118.1, 117.6, 110.3, 107.0, 38.1, 25.3, 23.0, 22.8, 11.3. HRMS (ESI$^+$): Calc.d for $C_{16}H_{18}NO^+$ ([M+H]$^+$) 240.1382, found 240.1379.

2-((5-Methyl-1*H*-pyrrol-2-yl)methyl)cyclohex-2-en-1-one (1v). A conical flask was charged with PBS pH 7.4 (224.3 mL), MeCN (17 mL), **1** (25 mM in MeCN, 4 mL), **v** (50 mM in MeCN, 4 mL) and BH_{MeHis}1.6[1] (150 µM in PBS pH 7.4, 667 µL), and incubated at 30 °C, 120 r.p.m. overnight. The reaction was quenched with MeCN (250 mL) and incubated further for 1 hour. Protein precipitate was removed by centrifugation (3,220 x g for 10 min) and the supernatant was concentrated under reduced pressure to ~200 mL. The concentrate was filtered and the product extracted with EtOAc (3 x 75 mL). The combined organic extracts were washed with 0.1 M NaOH (4 x 50 mL) and brine (2 x 50 mL), dried over $MgSO_4$, filtered, and concentrated under reduced pressure. The crude product was purified by silica column chromatography (4:1 cyclohexane:EtOAc) to give the product as a brown oil. Yield 6.9 mg (36.5%). ^{1}H NMR (400 MHz, $CDCl_3$) δ 8.31 (s, 1H), 6.83 (t, J = 4.3 Hz, 1H), 5.74 (d, J = 2.9 Hz, 1H), 5.70 (d, J = 2.9 Hz, 1H), 3.40 (s, 2H), 2.49 – 2.43 (m, 2H), 2.34 (q, J = 5.6 Hz, 2H), 2.21 (s, 3H), 1.99 (p, J = 6.3 Hz, 2H). ^{13}C NMR (101 MHz, $CDCl_3$) δ 201.4, 147.6, 138.8, 129.0, 127.0, 105.4, 105.4, 38.6, 29.6, 26.2, 23.0, 13.2. ^{1}H COSY shown in Supplementary Fig. 9. HRMS (ESI$^+$): Calc.d for $C_{12}H_{16}NO^+$ ([M+H]$^+$) 190.1226, found 190.1225.

1-((6-Oxocyclohex-1-en-1-yl)methyl)indoline-2,3-dione (1viii). A conical flask was charged with PBS pH 7.4 (224.3 mL), MeCN (17 mL), **1** (25 mM in MeCN, 4 mL), **viii** (50 mM in MeCN, 4 mL) and $BH_{MeHis}1.6$[1] (150 µM in PBS pH 7.4, 667 µL), and incubated at 30 °C, 120 r.p.m. for 3 days. The reaction was quenched with MeCN (250 mL) and incubated further for 1 hour. The mixture was filtered, and the filtrate was concentrated under reduced pressure to ~200 mL. The product was extracted with EtOAc (3 x 75 mL), and the combined organic extracts were washed with 0.1 M NaOH (4 x 50 mL) and brine (2 x 50 mL), dried over $MgSO_4$, filtered, and concentrated under reduced pressure. The crude product was purified by silica column chromatography (3:1 cyclohexane:EtOAc) to give the product as a yellow powder. Yield 15.9 mg (62.3%). ^{1}H NMR (400 MHz, $CDCl_3$) δ 7.66 – 7.49 (m, 2H), 7.11 (t, J = 7.5 Hz, 1H), 7.06 – 6.97 (m, 2H), 4.51 (s, 2H), 2.53 – 2.45 (m, 2H), 2.39 (ddq, J = 7.9, 3.8, 1.9 Hz, 2H), 2.01 (p, J = 6.2 Hz, 2H). ^{13}C NMR (101 MHz, $CDCl_3$) δ 198.6, 183.4, 158.8, 150.9, 149.3, 138.7, 132.9, 125.4, 124.0, 117.7, 111.4, 38.4, 38.2, 26.0, 22.7. HRMS (ESI^+): Calc.d for $C_{15}H_{14}NO_3^+$ ($[M+H]^+$) 256.0967, found 256.0966.

Ethyl 2-cyano-2-methyl-3-(6-oxocyclohex-1-en-1-yl)propanoate (1xi). A round bottom flask was charged with **S4** (100 mg, 0.595 mmol), THF (2.23 mL), H_2O (0.74 mL), and 1,4-diazabicyclo[2.2.2]octane (133.3 mg, 1.188 mmol), which was stirred at room temperature for 15 min. **xi** (83.2 mg, 0.655 mmol) was added and the reaction was stirred at room temperature for 7 days. The reaction mixture was acidified with 1 M HCl to pH 5, then the product was extracted with EtOAc (3 x 20 mL). The combined organic extracts were washed with brine (2 x 30 mL), dried over $MgSO_4$, filtered, and concentrated under reduced pressure. The crude product was purified by silica column chromatography (3:1 cyclohexane:EtOAc) to give the product as a brown oil. Yield 70.2 mg (50.2%). ^{1}H NMR (400 MHz, $CDCl_3$) δ 7.04 (t, J = 4.2 Hz, 1H), 4.28 – 4.15 (m, 2H), 2.83 (d, J = 13.6 Hz, 1H), 2.73 (d, J = 13.7 Hz, 1H), 2.49 – 2.40 (m, 4H), 2.02 (p, J = 6.4 Hz, 2H), 1.55 (s, 3H), 1.31 (t, J = 7.2 Hz, 3H). ^{13}C NMR (101 MHz, $CDCl_3$) δ 198.3, 168.8, 150.7, 134.0, 119.9, 63.0, 44.6, 38.2, 36.3, 26.5, 23.2, 22.9, 14.1. HRMS (ESI^+): Calc.d for $C_{13}H_{18}NO_3^+$ ($[M+H]^+$) 236.1280, found 236.1279.

Methyl 2-oxo-1-((6-oxocyclohex-1-en-1-yl)methyl)cyclopentane-1-carboxylate (1xiii). Two conical flasks were each charged with PBS pH 7.4 (4 mL), DMSO (3 mL), **S4** (100 mM in DMSO, 3 mL), **xiii** (200 mM in DMSO, 6 mL) and BH32.8[8] (550 µM in PBS pH 7.4, 4 mL), and incubated at 30 °C, 180 r.p.m. for 7 days. The reactions were pooled and the product was extracted with EtOAc (2 x 100 mL). The combined organic extracts were washed with H_2O (3 x 50 mL) and brine (2 x 50 mL), dried over $MgSO_4$, filtered, and concentrated under reduced

pressure. The crude product was purified by silica column chromatography (column 1: neat DCM, column 2: 10:1 to 1:1 *n*-hexane:EtOAc) to give the product as a brown oil. Yield 55.8 mg (37.2%). ^{1}H NMR (400 MHz, $CDCl_3$) δ 6.88 (t, J = 4.2 Hz, 1H), 3.69 (s, 3H), 2.89 (d, J = 13.8 Hz, 1H), 2.58 (d, J = 13.8 Hz, 1H), 2.43 – 2.21 (m, 7H), 2.02 – 1.80 (m, 5H). ^{13}C NMR (101 MHz, $CDCl_3$) δ 214.6, 199.4, 171.6, 149.6, 135.5, 61.1, 52.6, 38.4, 38.1, 33.4, 32.0, 26.5, 23.0, 19.7. HRMS (ESI^+): Calc.d for $C_{14}H_{19}O_4^+$ ($[M+H]^+$) 251.1277, found 251.1275. CD and absorbance spectra shown in Supplementary Fig. 10a.

Methyl 2-oxo-1-((6-oxocyclohex-1-en-1-yl)methyl)cyclohexane-1-carboxylate (1xiv). A round bottom flask was charged with **S1** (126.2 mg, 1 mmol), DCM (10 mL), **xiv** (312.4 mg, 2 mmol) and $FeCl_3\cdot6H_2O$ (27.0 mg, 0.1 mmol), which was stirred at 50 °C for 12 hours. The reaction mixture was washed with H_2O (3 x 5 mL), dried over $MgSO_4$, filtered, and concentrated under reduced pressure. The crude product was purified by silica column chromatography (4:1 to 2:1 hexane:EtOAc) to give the product as a brown oil. Yield 26.0 mg (9.8%). ^{1}H NMR (400 MHz, $CDCl_3$) δ 6.72 (t, J = 4.2 Hz, 1H), 3.68 (s, 3H), 2.95 (d, J = 13.7 Hz, 1H), 2.45 – 2.30 (m, 9H), 2.01 – 1.92 (m, 3H), 1.67 – 1.53 (m, 3H). ^{13}C NMR (101 MHz, $CDCl_3$) δ 207.4, 199.0, 171.5, 149.4, 135.3, 60.6, 52.5, 41.3, 38.4, 35.9, 33.4, 27.7, 26.5, 23.0, 22.6. HRMS (ESI^+): Calc.d for $C_{15}H_{21}O_4^+$ ($[M+H]^+$) 265.1434, found 265.1427.

Methyl 2-((3-acetyl-2-oxotetrahydrofuran-3-yl)methyl)acrylate (2ii). A round bottom flask was charged with **S5** (200 mg, 1.265 mmol), THF (2 mL), H_2O (2 mL), and 1,4-diazabicyclo[2.2.2]octane (170.2 mg, 1.518 mmol), which was stirred at room temperature for 15 min. **ii** (178.2 mg, 1.391 mmol) was added and the reaction was stirred at room temperature overnight. The reaction mixture was acidified with 0.5 M HCl to pH 5, then the product was extracted with EtOAc (3 x 5 mL). The combined organic extracts were washed with 1 M $NaHCO_3$ (10 mL) and brine (2 x 10 mL), dried over $MgSO_4$, filtered, and concentrated under reduced pressure. The crude product was purified by silica column chromatography (4:1 to 3:1 cyclohexane:EtOAc) to give the product as a white powder. Yield 190 mg (66.4%). ^{1}H NMR (400 MHz, $CDCl_3$) δ 6.31 (s, 1H), 5.67 (s, 1H), 4.26 (td, J = 8.8, 3.2 Hz, 1H), 4.14 (dt, J = 9.0, 7.0 Hz, 1H), 3.75 (s, 3H), 3.24 (dd, J = 14.9, 1.1 Hz, 1H), 2.89 – 2.78 (m, 2H), 2.36 (s, 3H), 2.12 – 2.04 (m, 1H). ^{13}C NMR (101 MHz, $CDCl_3$) δ 201.9, 175.3, 167.1, 135.3, 129.6, 66.6, 61.3, 52.4, 35.6, 29.2, 26.0. HRMS (ESI^+): Calc.d for $C_{11}H_{14}O_5Na^+$ ($[M+Na]^+$) 249.0733, found 249.0728.

1-Ethyl 5-methyl 2-cyano-4-methylene-2-phenylpentanedioate (2xii). A round bottom flask was charged with **S5** (100 mg, 0.632 mmol), THF (1 mL), H_2O (1 mL), and 1,4-diazabicyclo[2.2.2]octane (85.1 mg, 0.759 mmol), which was stirred at room temperature for 15 min. **xii** (131.7 mg, 0.696 mmol) was added and the reaction was stirred at room temperature overnight. The reaction mixture was acidified with 1 M HCl to pH 5, then the product was extracted with EtOAc (3 x 4 mL). The combined organic extracts were washed with 1 M $NaHCO_3$ (10 mL) and brine (2 x 8 mL), dried over $MgSO_4$, filtered, and concentrated under reduced pressure. The crude product was purified by silica column chromatography (4:1 cyclohexane:EtOAc to neat EtOAc) to give the product as a brown oil. Yield 129.2 mg (71.1%). ^{1}H NMR (400 MHz, $CDCl_3$) δ 7.56 – 7.49 (m, 2H), 7.43 – 7.35 (m, 3H), 6.34 (s, 1H), 5.69 (s, 1H), 4.25 (q, J = 7.1 Hz, 2H), 3.65 (s, 3H), 3.40 (d, J = 14.5 Hz, 1H), 3.25 (d, J = 14.4 Hz, 1H), 1.25 (t, J = 7.1 Hz, 3H). ^{13}C NMR (101 MHz, $CDCl_3$) δ 167.2, 166.9, 134.5, 134.0, 130.3, 129.2, 129.1, 126.5, 117.8, 63.6, 54.1, 52.3, 38.7, 13.9. HRMS (ESI$^+$): Calc.d for $C_{16}H_{18}NO_4^+$ ([M+H]$^+$) 288.1230, found 288.1229.

Methyl 1-(2-(methoxycarbonyl)allyl)-2-oxocyclopentane-1-carboxylate (2xiii). A round bottom flask was charged with **S5** (100 mg, 0.632 mmol), THF (1 mL), H_2O (1 mL), and 1,4-diazabicyclo[2.2.2]octane (85.1 mg, 0.759 mmol), which was stirred at room temperature for 15 min. **xiii** (98.9 mg, 0.696 mmol) was added and the reaction was stirred at room temperature for 5 days. The reaction mixture was acidified with 0.5 M HCl to pH 5, then the product was extracted with EtOAc (3 x 5 mL). The combined organic extracts were washed with 1 M $NaHCO_3$ (10 mL) and brine (2 x 10 mL), dried over $MgSO_4$, filtered, and concentrated under reduced pressure. The crude product was purified by silica column chromatography (4:1 cyclohexane:EtOAc) to give the product as a colourless oil. Yield 116.7 mg (76.8%). ^{1}H NMR (400 MHz, $CDCl_3$) δ 6.25 (d, J = 1.3 Hz, 1H), 5.66 (d, J = 1.3 Hz, 1H), 3.74 (s, 3H), 3.69 (s, 3H), 3.04 (dd, J = 14.3, 1.0 Hz, 1H), 2.70 (dd, J = 14.3, 1.0 Hz, 1H), 2.49 – 2.22 (m, 3H), 1.99 – 1.85 (m, 3H). ^{13}C NMR (101 MHz, $CDCl_3$) δ 214.1, 171.1, 167.7, 136.3, 129.1, 61.0, 52.8, 52.2, 38.0, 34.6, 32.9, 19.7. HRMS (ESI$^+$): Calc.d for $C_{12}H_{16}O_5Na^+$ ([M+Na]$^+$) 263.0890, found 263.0899.

2-(1-(2-Methyl-1*H*-indol-3-yl)ethyl)cyclohex-2-en-1-one (3iii). A conical flask was charged with PBS pH 7.4 (224.3 mL), MeCN (17 mL), **3** (25 mM in MeCN, 4 mL), **iii** (50 mM in MeCN, 4 mL) and $BH_{MeHis}1.6$[1] (150 µM in PBS pH 7.4, 667 µL), and incubated at 30 °C, 120 r.p.m.. Additional enzyme was added after 4 days (150 µM in PBS pH 7.4, 1.334 mL) and 8 days (150 µM in PBS pH 7.4, 2 mL). After 15 days the reaction was quenched with MeCN

(250 mL) and incubated further for 1 hour. The mixture was filtered, and the filtrate was concentrated under reduced pressure to ~200 mL. The product was extracted with EtOAc (3 x 75 mL), and the combined organic extracts were washed with 0.1 M NaOH (4 x 40 mL) and brine (2 x 50 mL), dried over $MgSO_4$, filtered, and concentrated under reduced pressure. The crude product was purified by silica column chromatography (5:1 to 4:1 cyclohexane:EtOAc) to give the product as a brown powder. Yield 9.6 mg (37.9%). ^1H NMR (400 MHz, $CDCl_3$) δ 7.73 (s, 1H), 7.53 (d, J = 7.8 Hz, 1H), 7.25 (d, J = 9.3 Hz, 1H), 7.03 (dt, J = 23.3, 7.3 Hz, 2H), 6.91 (t, J = 4.3 Hz, 1H), 4.35 – 4.24 (m, 1H), 2.43 (s, 3H), 2.38 (dd, J = 7.8, 5.5 Hz, 3H), 1.95 (dp, J = 18.7, 6.8 Hz, 3H), 1.52 (d, J = 7.3 Hz, 3H). ^{13}C NMR (101 MHz, $CDCl_3$) δ 199.5, 143.9, 143.2, 135.5, 131.4, 128.0, 120.5, 119.5, 119.0, 113.8, 110.5, 39.0, 29.6, 26.1, 23.1, 19.4, 12.4. HRMS (ESI^+): Calc.d for $C_{17}H_{20}NO^+$ ($[M+H]^+$) 254.1539, found 254.1549. CD and absorbance spectra shown in Supplementary Fig. 10b.

2-(1-(Phenylamino)ethyl)cyclohex-2-en-1-one (3vii). A round bottom flask was charged with **S6** (50 mg, 0.274 mmol), THF (0.5 mL), H_2O (0.5 mL), and 1,4-diazabicyclo[2.2.2]octane (36.9 mg, 0.329 mmol), which was stirred at room temperature for 15 min. **vii** (28.1 mg, 0.301 mmol) was added and the reaction was stirred at room temperature overnight. The reaction mixture was acidified with 1 M HCl to pH 5, then the product was extracted with EtOAc (4 x 3 mL). The combined organic extracts were washed with 1 M $NaHCO_3$ (10 mL) and brine (2 x 8 mL), dried over $MgSO_4$, filtered, and concentrated under reduced pressure. The crude product was purified by silica column chromatography (5:1 cyclohexane:EtOAc) to give the product as a brown powder. Yield 11.8 mg (20.0%). The spectral data are consistent with literature values[9]. ^1H NMR (400 MHz, $CDCl_3$) δ 7.15 – 7.09 (m, 2H), 6.90 (t, J = 4.2 Hz, 1H), 6.66 (t, J = 7.2 Hz, 1H), 6.48 (d, J = 8.0 Hz, 2H), 4.38 (q, J = 6.7 Hz, 1H), 3.97 (s, 1H), 2.44 (dd, J = 7.7, 5.8 Hz, 2H), 2.33 (q, J = 5.5 Hz, 2H), 1.95 (dtd, J = 8.3, 6.5, 2.1 Hz, 2H), 1.34 (d, J = 6.6 Hz, 3H). ^{13}C NMR (101 MHz, $CDCl_3$) δ 199.7, 147.0, 145.2, 140.7, 129.3, 117.4, 113.4, 48.6, 39.0, 25.9, 22.9, 22.3.

DNA and Protein Sequences for ASB1.3 and ASA1.5

MeHis = N_{δ}-methylhistidine

ASB1.3

ATGATTCGTGCGGTATTCTTTGATAGCCTGAGTACTCTGATTAGCGTTGAAGGCGCT
TATAAAATTTAGGTGAAAGAGATGGAGGAAGTGCTGGGTGACTATCCGCTGAACC
CGAAAACCCTGTGGGACGAATTTGATAAACTGTTTCGCCCAGCGTTCTCTAACTAT
GCGGGCAAACCGTATCGTCCGGCGCGTGATATCATGGAAGAAGTAATGCGTAAAC
TGGCGGAAAAGTACGGTTTCAAATACCCTGGAAACTTGCAGGAAATCGGCGTGCG
TATGGCGCGACGCTACGGCGATCTGTACCCGGAAGTGGTGGAAGTACTGAAATCT
CTGAAAGGTAAATATCACGTTGGCGTGATCCTGAATTGGGATACCGAGAATGCCAC
GGCATTCCTGGACGCACTGGGCATCAAAGACCTGTTCGATTCCATCACCACGTCTG
AAGAAGCTGGTTTCGCTTTTCCGCACCCACGCATCCTCGAACTGGCTCTGAAGAA
AGCCGGCGTTAAAGGCGAGAAAGCAGTGTACGTTGGTGACAACCCGGTCAAAGA
CGCGGGTGGTTCTAAGAACCTGGGTATGACTAGCATCCTGCTGGATCGTAAAGGT
GAGAAACGTGAATTCTGGGATAAGGCGGACTTTCGTGTCTCCGACCTGCGCGAAG
TTATTAAGATTGTTGACGAACTGAACGGTCAGGGCTCTCTCGAGCACCACCACCA
CCACCACTGA

MIRAVFFDSLSTLISVEGAYKI **(MeHis)** VKEMEEVLGDYPLNPKTLWDEFDKLFRPAF
SNYAGKPYRPARDIMEEVMRKLAEKYGFKYPGNLQEIGVRMARRYGDLYPEVVEVL
KSLKGKYHVGVILNWDTENATAFLDALGIKDLFDSITTSEEAGFAFPHPRILELALKK
AGVKGEKAVYVGDNPVKDAGGSKNLGMTSILLDRKGEKREFWDKADFRVSDLREV
IKIVDELNGQGSLEHHHHHH

ASA1.5

ATGATTCGTGCGGTATTCTTTGATAGCCTGCGGACTCTGATTAGCGTTGAAGGCGC
TTATAAAATTTAGGGGAAAGAGATGGAGGAAGTGCTGGGTGACTATCCGCTGAAC
CCGCGAACCCTGTGGGACGAATTTGATAAACTGTTTCAGGAAGCGTTCTCTAACTA
TGCGGGCAAACCGTATCGTCCGGCGCGTTTTATCACGGAAGAAGTAATGCGTAAA
CTGGCGGAAAAGTACGGTTTCAAATACCCTGGAAACTTGCAGGAAATCGGCGTGC
GTATGGCGCGACGCTACGGCGGGCTGTACCCGGAAGTGGTGGAAGTACTGAAATC
TCTGAAAGGTAAATATCACGTTGGCGTGGCTCTGGATTGGGATACCGAGAATGCCA
CGGCATGGCTGGACGCACTGGGCATCAAAGACCTGTTCGATTCCATCACCACGTC
TGAAGAAGCTGGTTTCGCTTTCCCGCACCCACGCGTGCTCGAACTGGCTCTGAAG
AAAGCCGGCGTTAAAGGCGAGAAAGCAGTGTACGTTGGTGACTGGTGTGTCAAA
GACGCGGGTGGTTCTAAGAACCTGGGTATGACTAGCATCCTGCTGGATCGTAAAG
GTGAGAAACGTTCATTCTGGGATAAGGCGGACTTTCGTGTCTCCGACCTGCGCGA
AGTTATTAAGATTGTTGACGAACTGAACGGTCAGGGCTCTCTCGAGCACCACCAC
CACCACCACTGA

MIRAVFFDSLRTLISVEGAYKI **(MeHis)** GKEMEEVLGDYPLNPRTLWDEFDKLFQEAF
SNYAGKPYRPARFITEEVMRKLAEKYGFKYPGNLQEIGVRMARRYGGLYPEVVEVL
KSLKGKYHVGVALDWDTENATAWLDALGIKDLFDSITTSEEAGFAFPHPRVLELALK
KAGVKGEKAVYVGDWCVKDAGGSKNLGMTSILLDRKGEKRSFWDKADFRVSDLRE
VIKIVDELNGQGSLEHHHHHH

Chiral HPLC Chromatograms

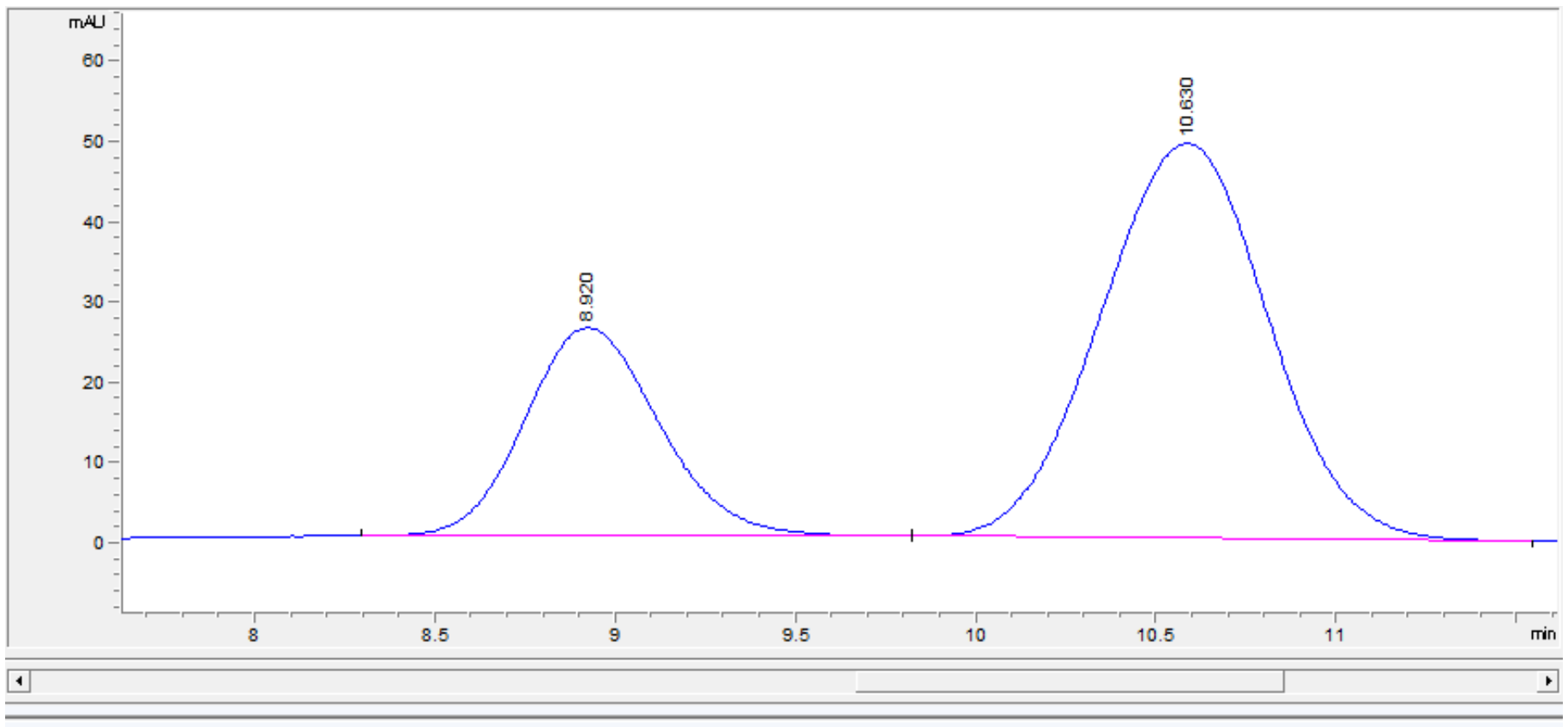

#	Time	Area	Height	Width	Area%	Symmetry
1	8.92	646.3	25.9	0.3851	29.614	0.847
2	10.63	1536.1	49.2	0.4942	70.386	1.03

Chiral HPLC chromatogram of **1i** standard

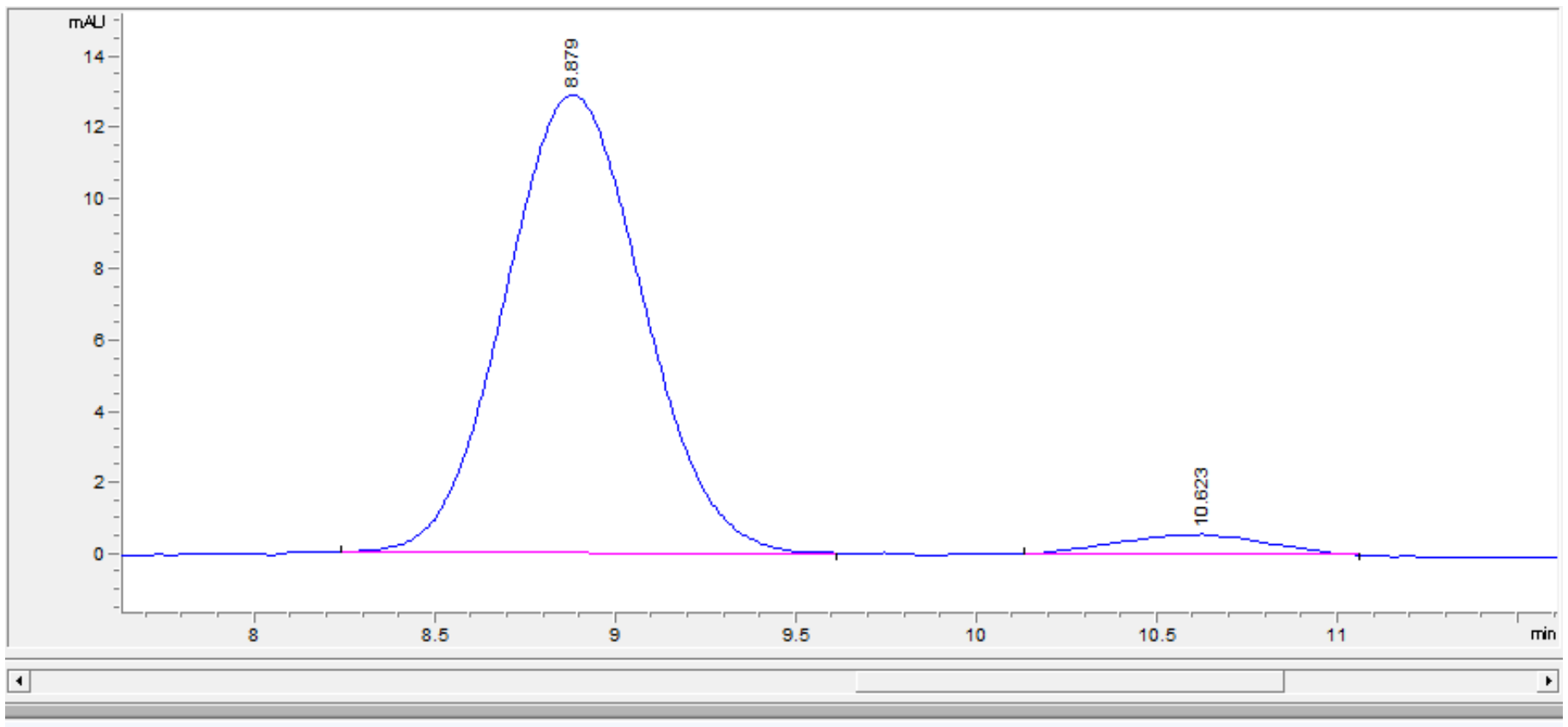

#	Time	Area	Height	Width	Area%	Symmetry
1	8.879	343.7	12.9	0.4438	95.435	0.929
2	10.623	16.4	5.8E-1	0.4752	4.565	1.222

Chiral HPLC chromatogram of (*R*)-**1i** from a biotransformation

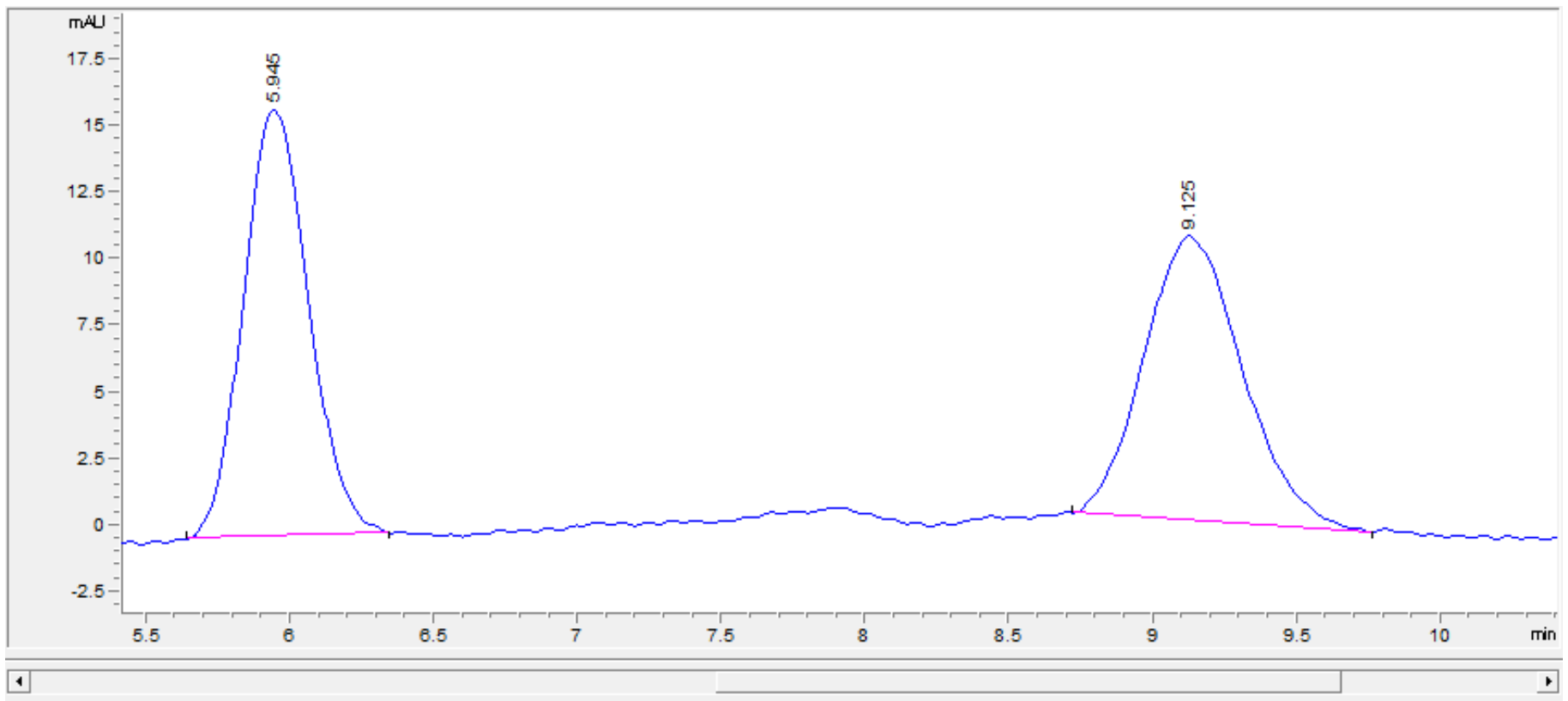

#	Time	Area	Height	Width	Area%	Symmetry
1	5.945	254	16.1	0.2486	49.870	0.87
2	9.125	255.3	10.7	0.3267	50.130	0.825

Chiral HPLC chromatogram of **1ii** standard

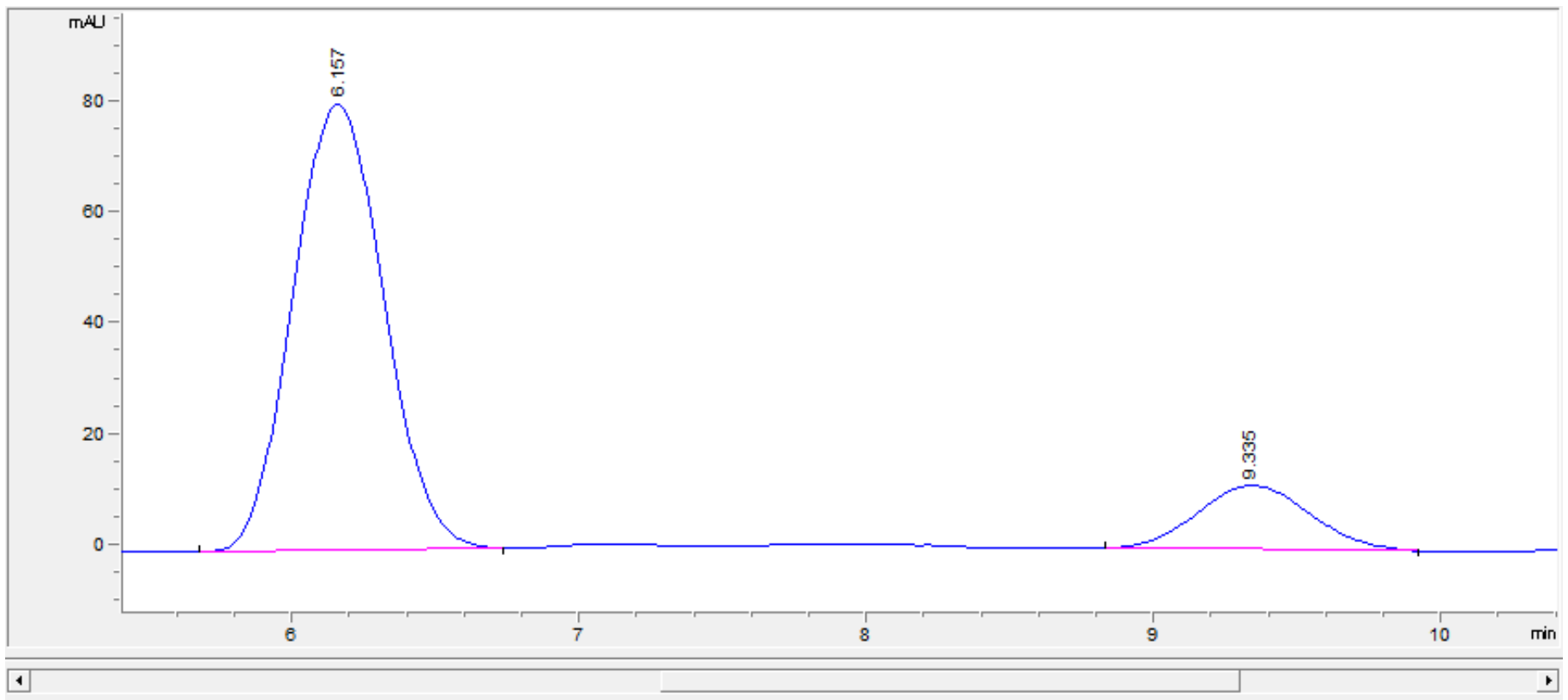

#	Time	Area	Height	Width	Area%	Symmetry
1	6.157	1772.3	80.5	0.3509	84.881	0.939
2	9.335	315.7	11.5	0.4572	15.119	0.885

Chiral HPLC chromatogram of **1ii** from a biotransformation

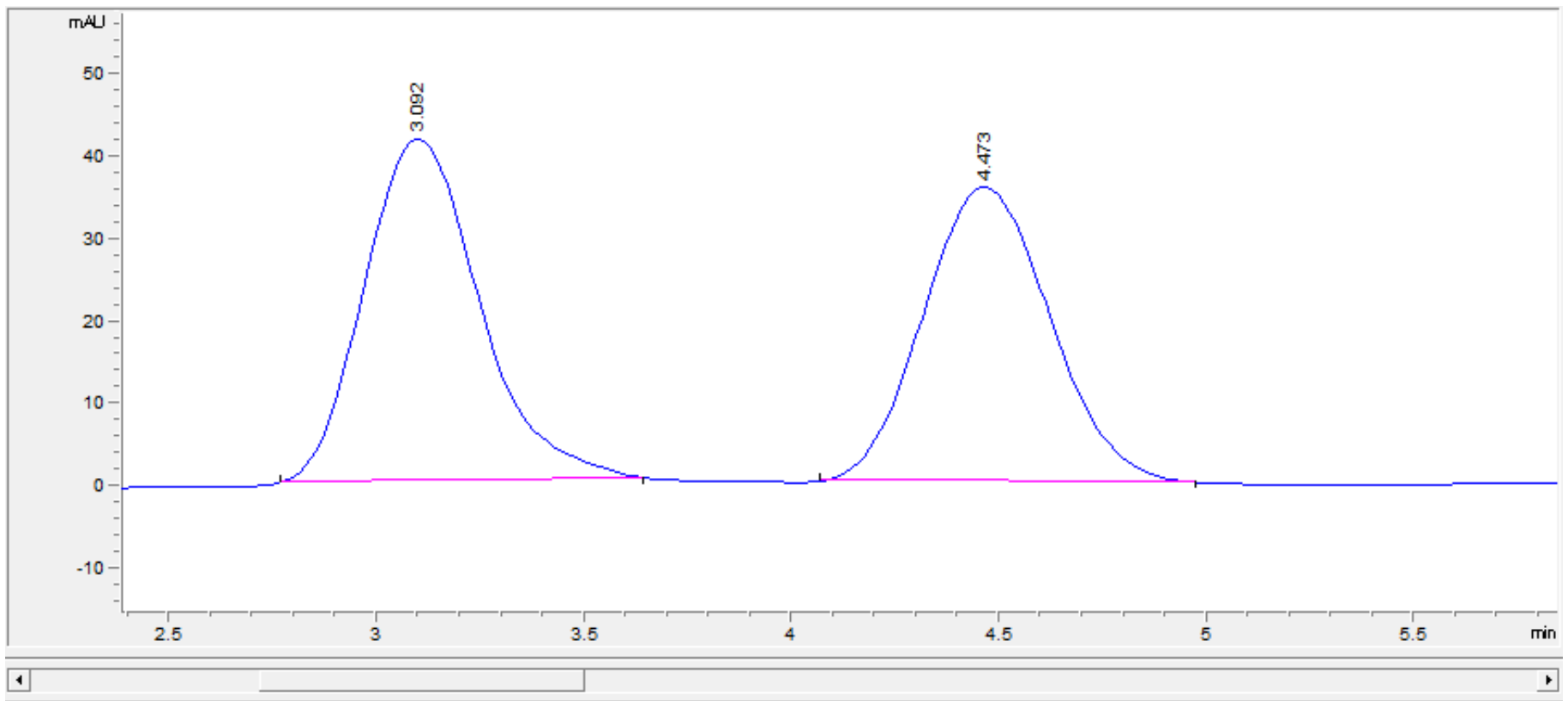

#	Time	Area	Height	Width	Area%	Symmetry
1	3.092	378.3	41.6	0.1517	51.386	0.852
2	4.473	357.9	35.7	0.1669	48.614	0.913

Chiral HPLC chromatogram of **1xi** standard

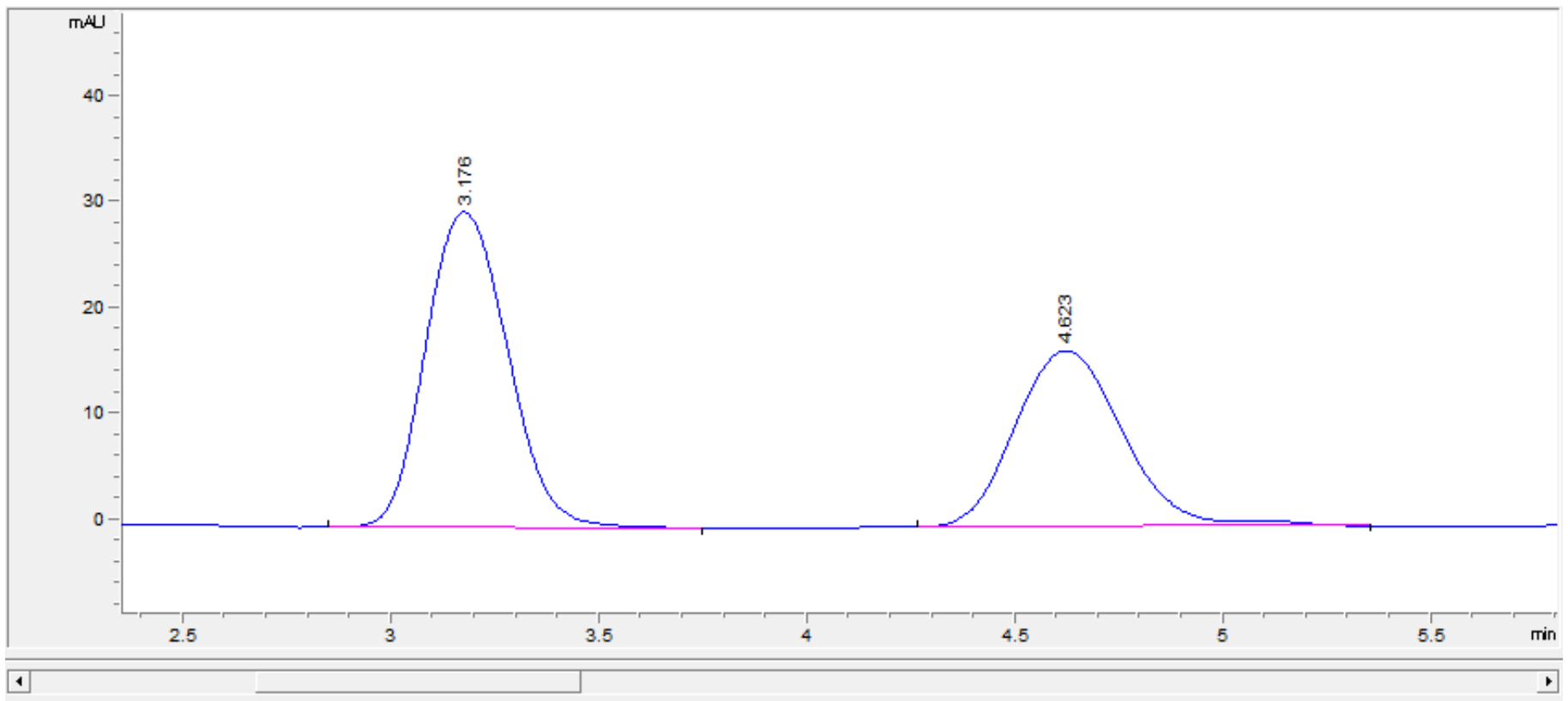

#	Time	Area	Height	Width	Area%	Symmetry
1	3.176	409.3	29.8	0.2181	57.533	0.846
2	4.623	302.1	16.6	0.2904	42.467	0.839

Chiral HPLC chromatogram of **1xi** from a biotransformation

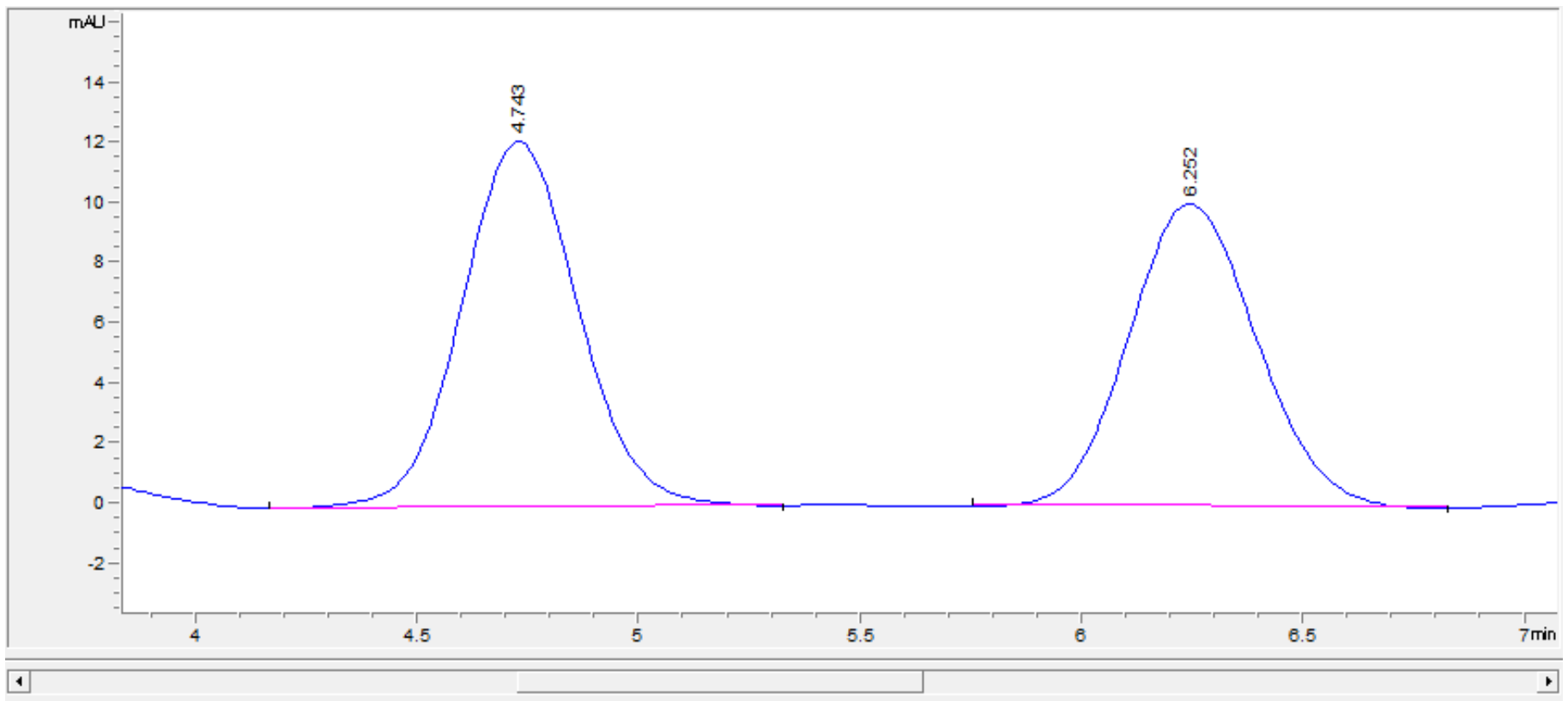

#	Time	Area	Height	Width	Area%	Symmetry
1	4.743	130.7	12.2	0.1673	52.302	0.93
2	6.252	119.2	10.1	0.1858	47.698	0.896

Chiral HPLC chromatogram of **1xiii** standard

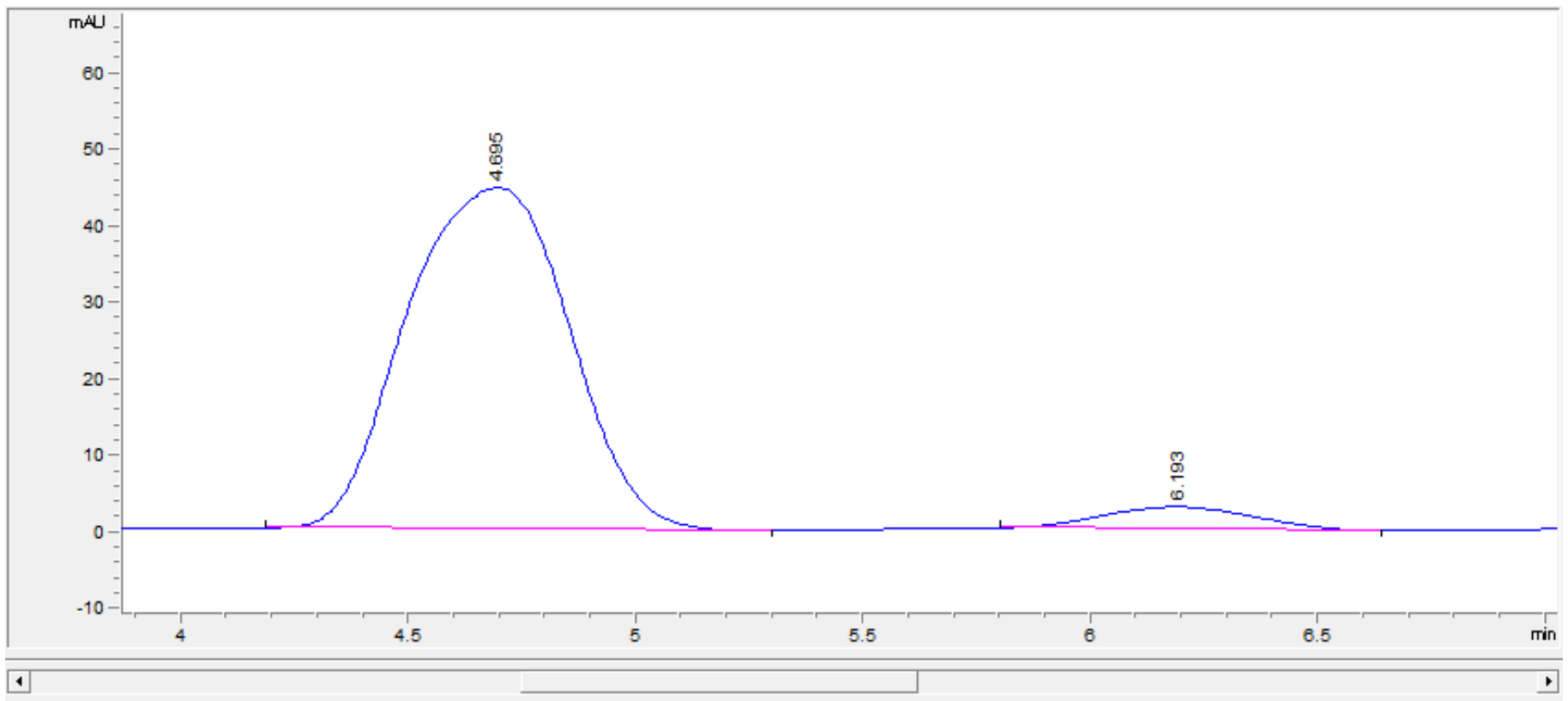

#	Time	Area	Height	Width	Area%	Symmetry
1	4.695	1107.6	44.6	0.4078	94.322	1.188
2	6.193	66.7	2.9	0.3012	5.678	0.981

Chiral HPLC chromatogram of **1xiii** from a biotransformation

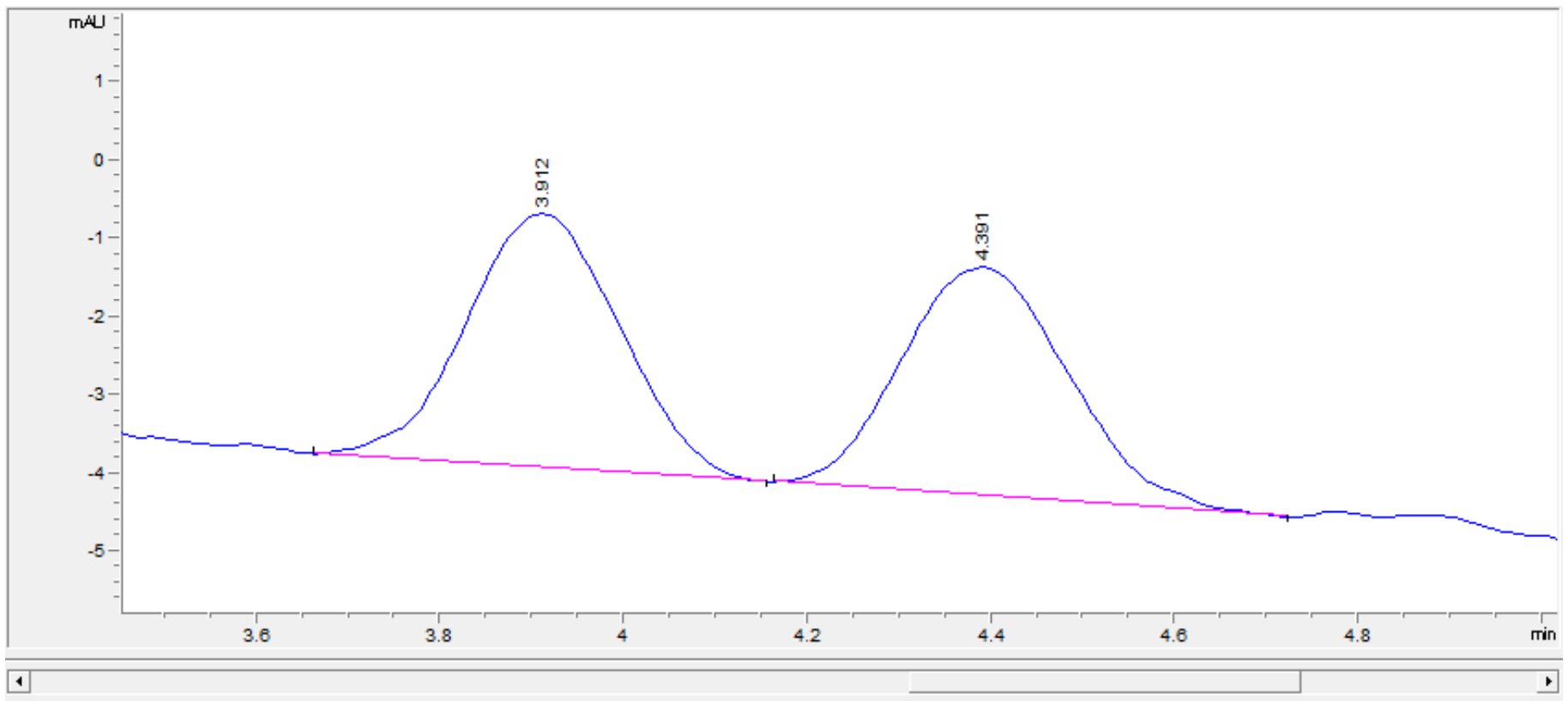

#	Time	Area	Height	Width	Area%	Symmetry
1	3.912	37.7	3.2	0.1714	50.518	0.991
2	4.391	36.9	2.9	0.1929	49.482	0.911

Chiral HPLC chromatogram of **1xiv** standard

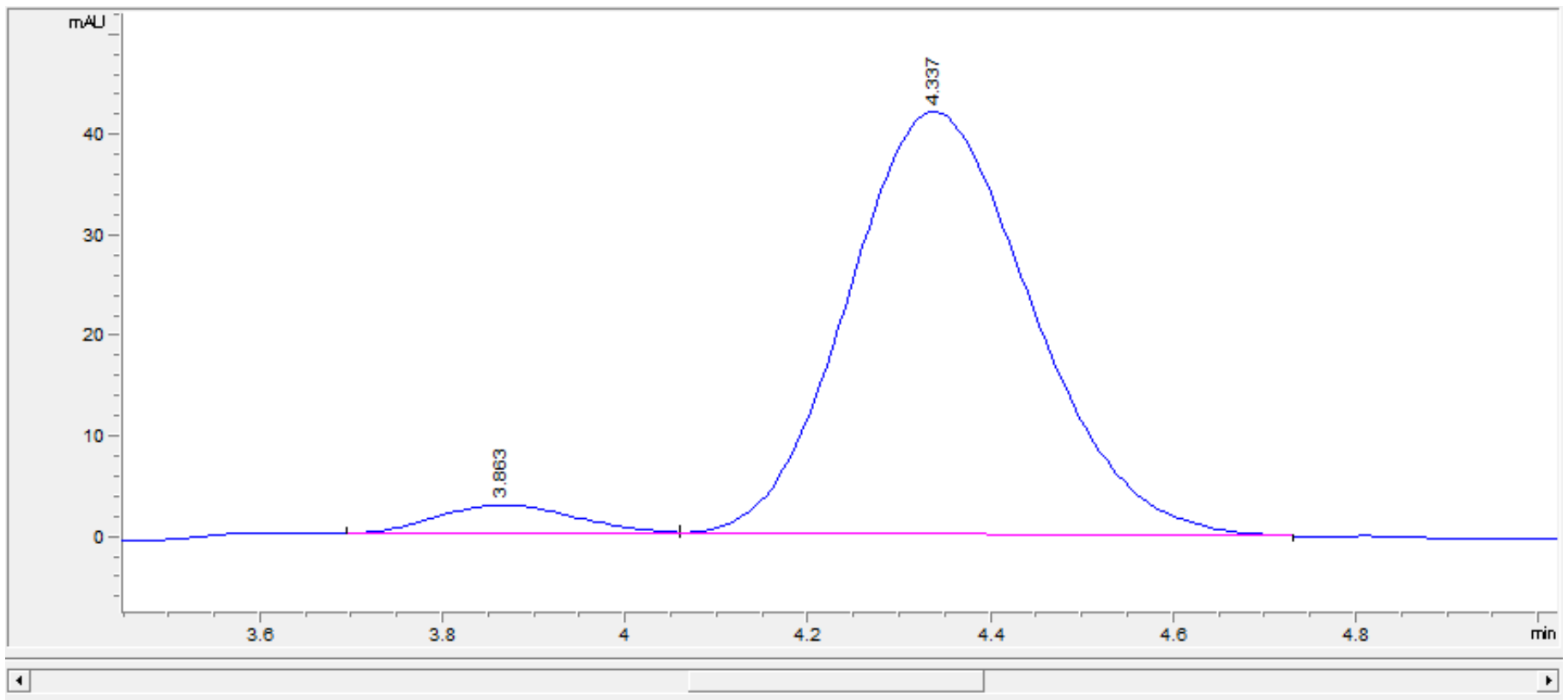

#	Time	Area	Height	Width	Area%	Symmetry
1	3.863	32	2.9	0.1819	5.125	0.813
2	4.337	592	42.2	0.234	94.875	0.856

Chiral HPLC chromatogram of **1xiv** from a biotransformation

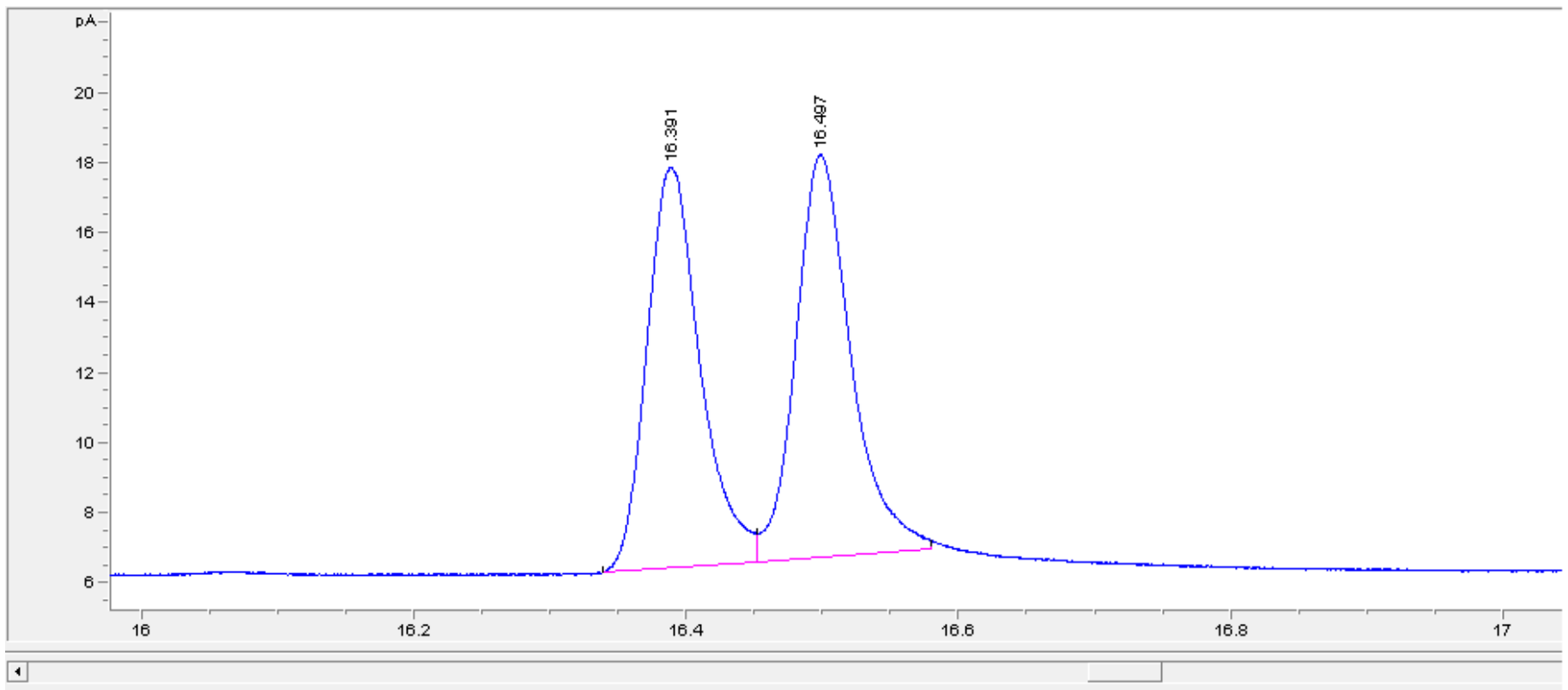

#	Time	Area	Height	Width	Area%	Symmetry
1	16.391	33.2	11.4	0.044	48.791	0.775
2	16.497	34.9	11.5	0.0466	51.209	0.77

Chiral GC chromatogram of **2ii** standard

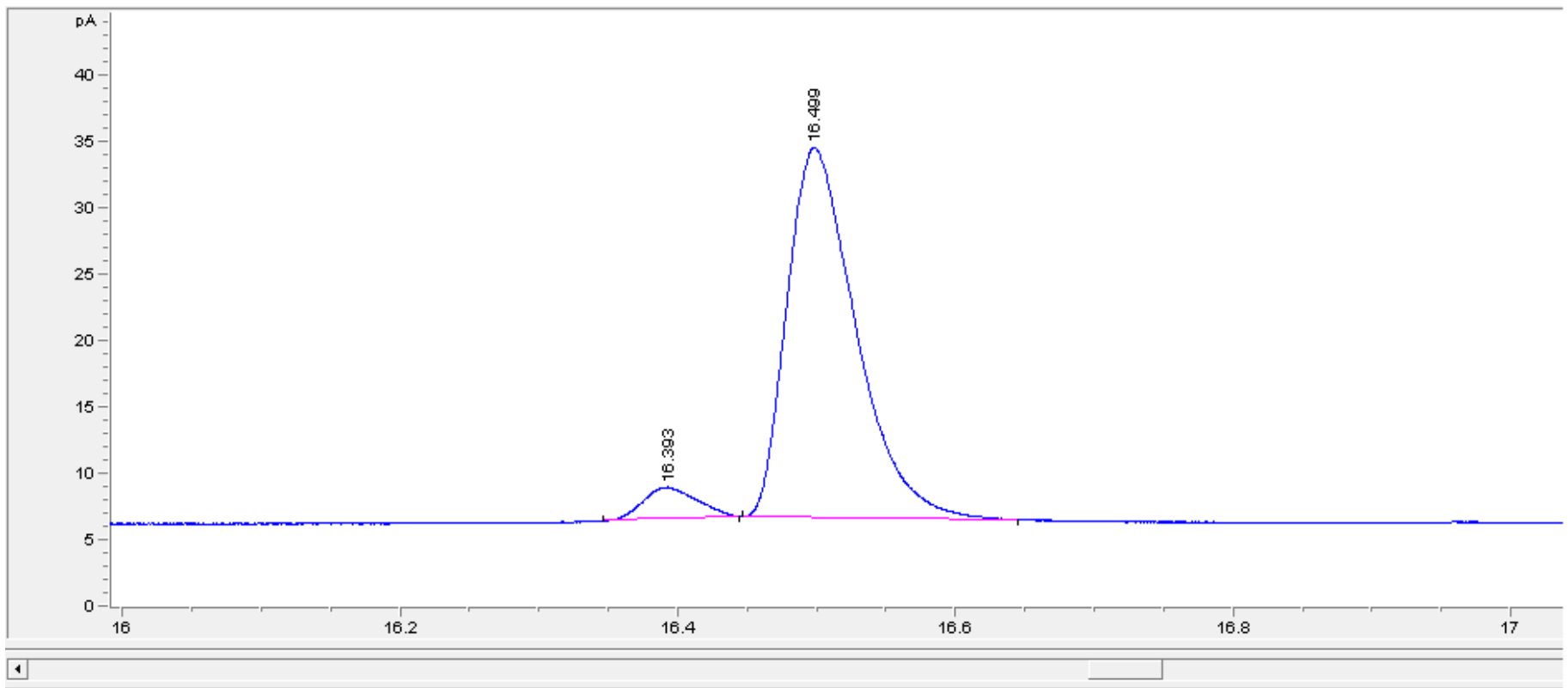

#	Time	Area	Height	Width	Area%	Symmetry
1	16.393	6.3	2.4	0.0444	6.154	0.859
2	16.499	95.6	27.8	0.0573	93.846	0.658

Chiral GC chromatogram of (*S*)-**2ii** from a biotransformation

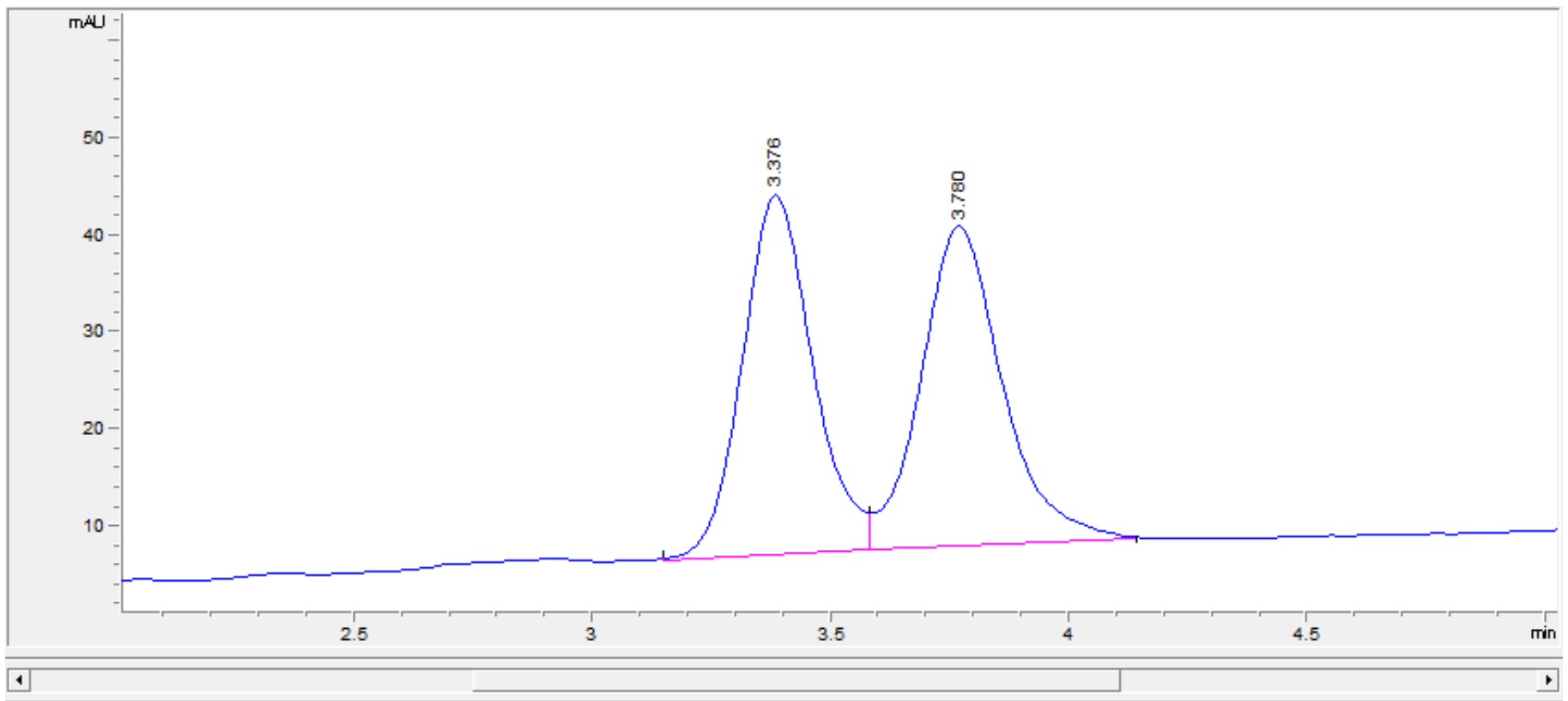

#	Time	Area	Height	Width	Area%	Symmetry
1	3.376	263.5	37.2	0.118	49.397	0.86
2	3.78	269.9	33	0.1362	50.603	0.85

Chiral HPLC chromatogram of **2xii** standard

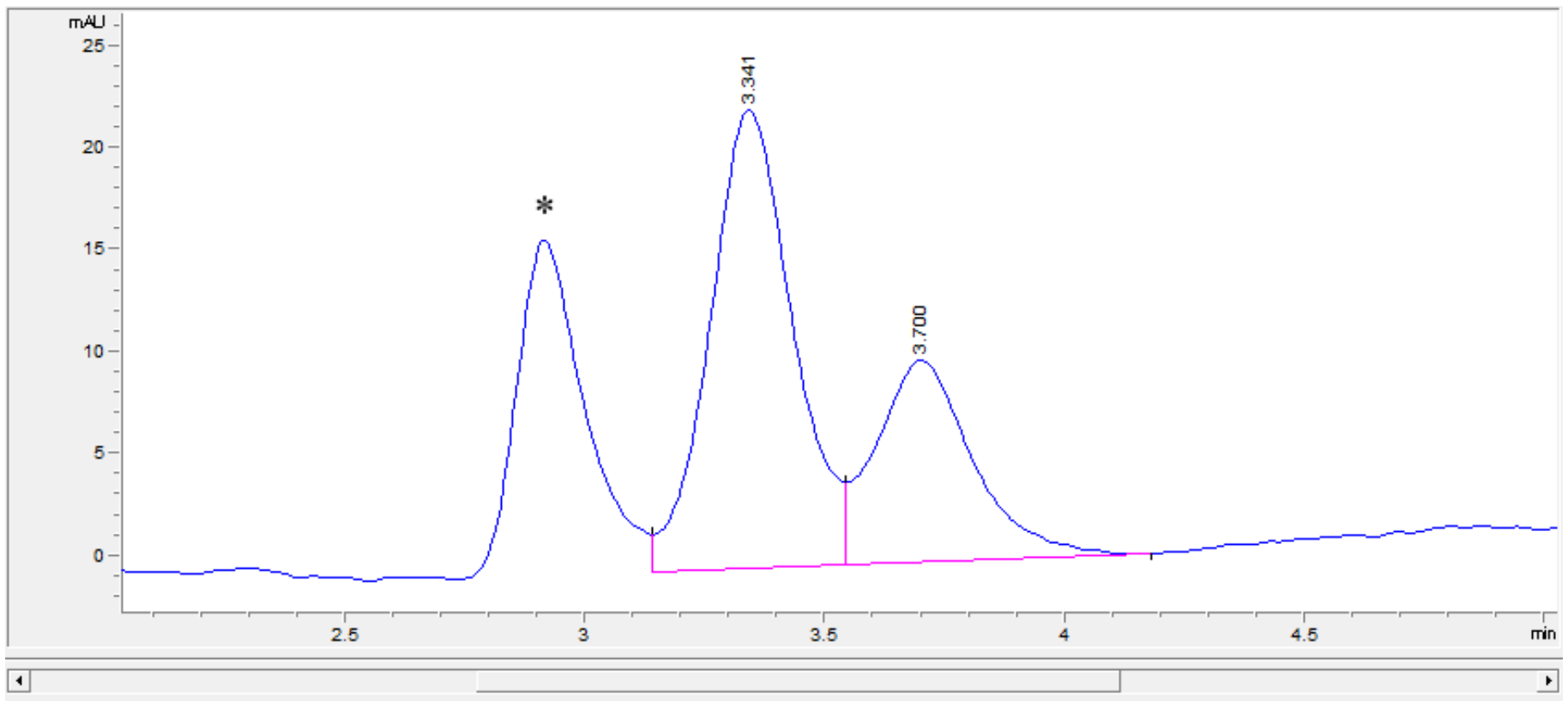

#	Time	Area	Height	Width	Area%	Symmetry
1	3.341	270.8	22.5	0.1785	65.847	0.861
2	3.7	140.5	9.9	0.2115	34.153	0.802

Chiral HPLC chromatogram of **2xii** from a biotransformation

* peak is PNP byproduct

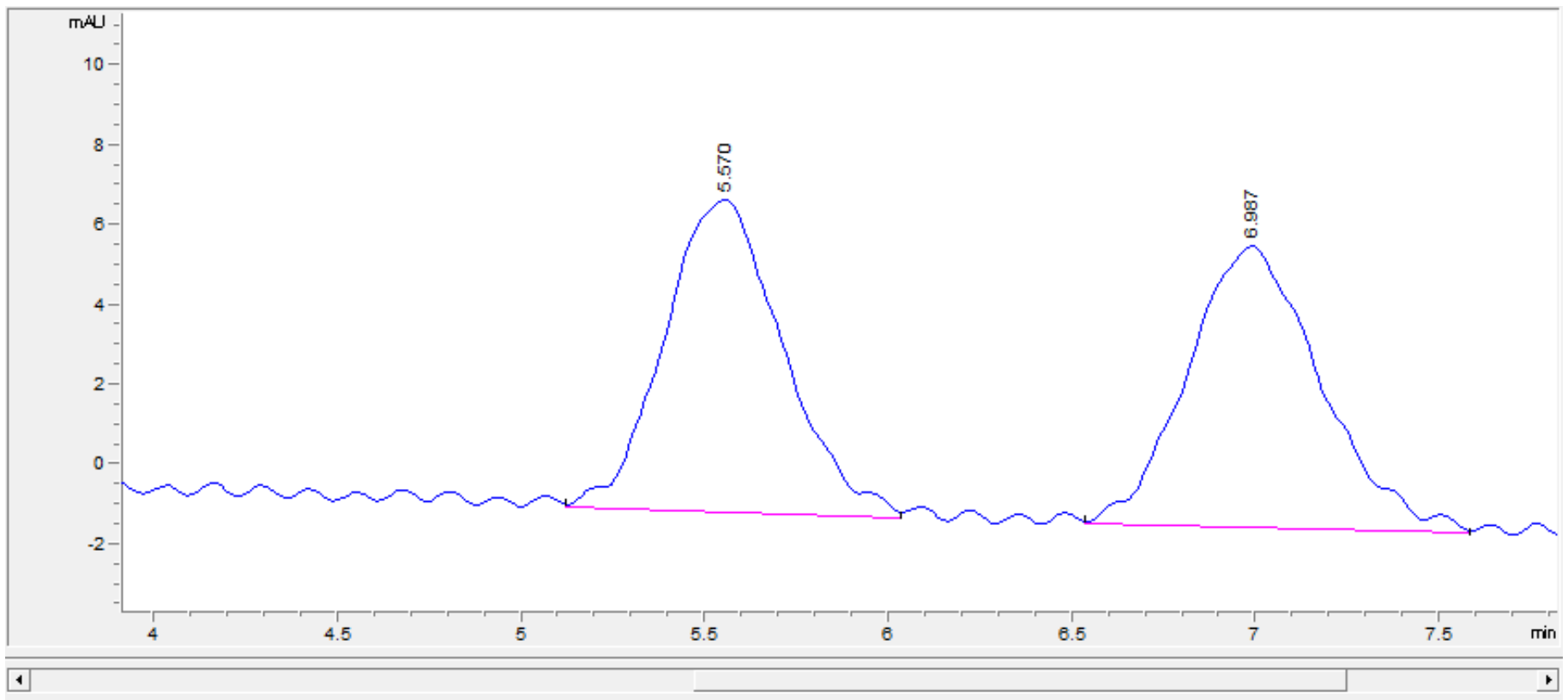

#	Time	Area	Height	Width	Area%	Symmetry
1	5.57	89	7.9	0.1642	49.698	0.975
2	6.987	90.1	7.1	0.1642	50.302	0.895

Chiral HPLC chromatogram of **2xiii** standard

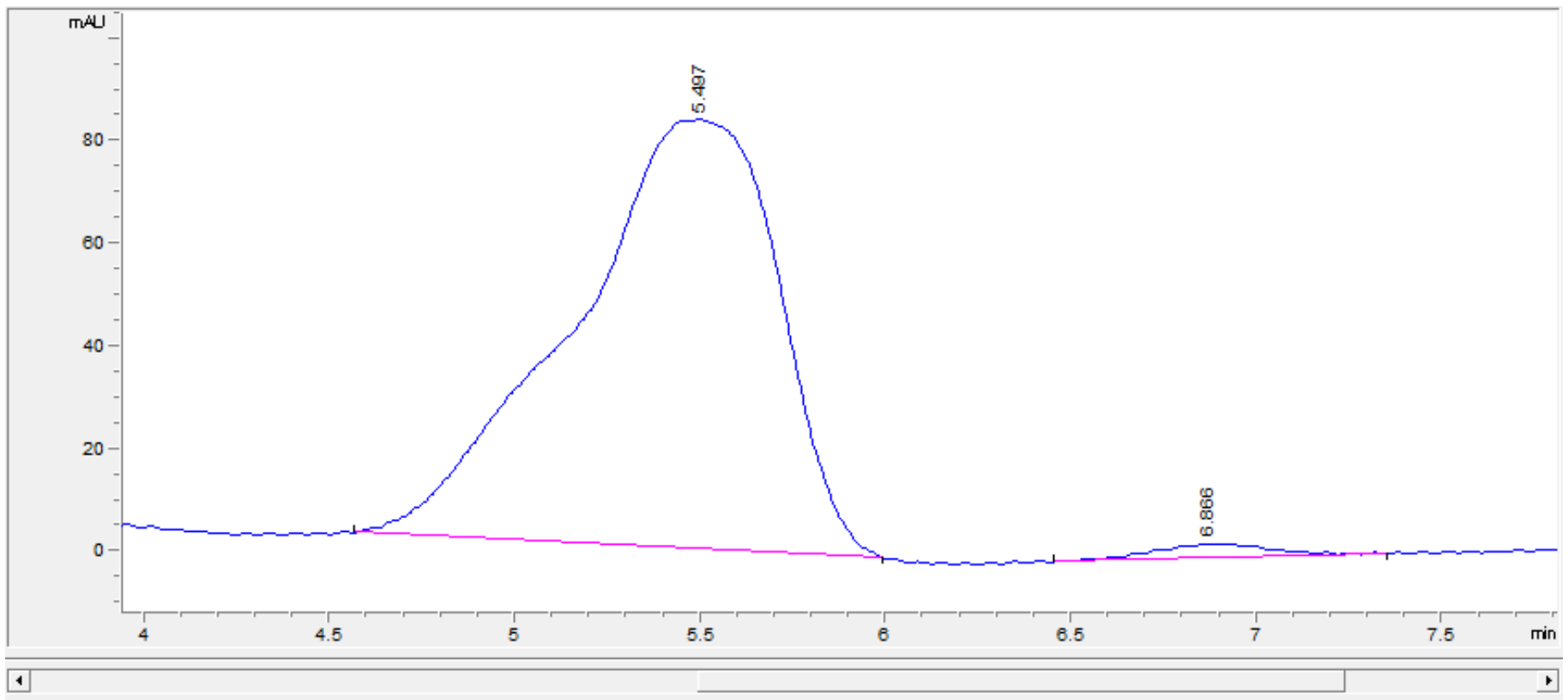

#	Time	Area	Height	Width	Area%	Symmetry
1	5.497	3215.7	83.7	0.6401	97.972	1.577
2	6.866	66.6	3	0.3741	2.028	0.889

Chiral HPLC chromatogram of **2xiii** from a biotransformation

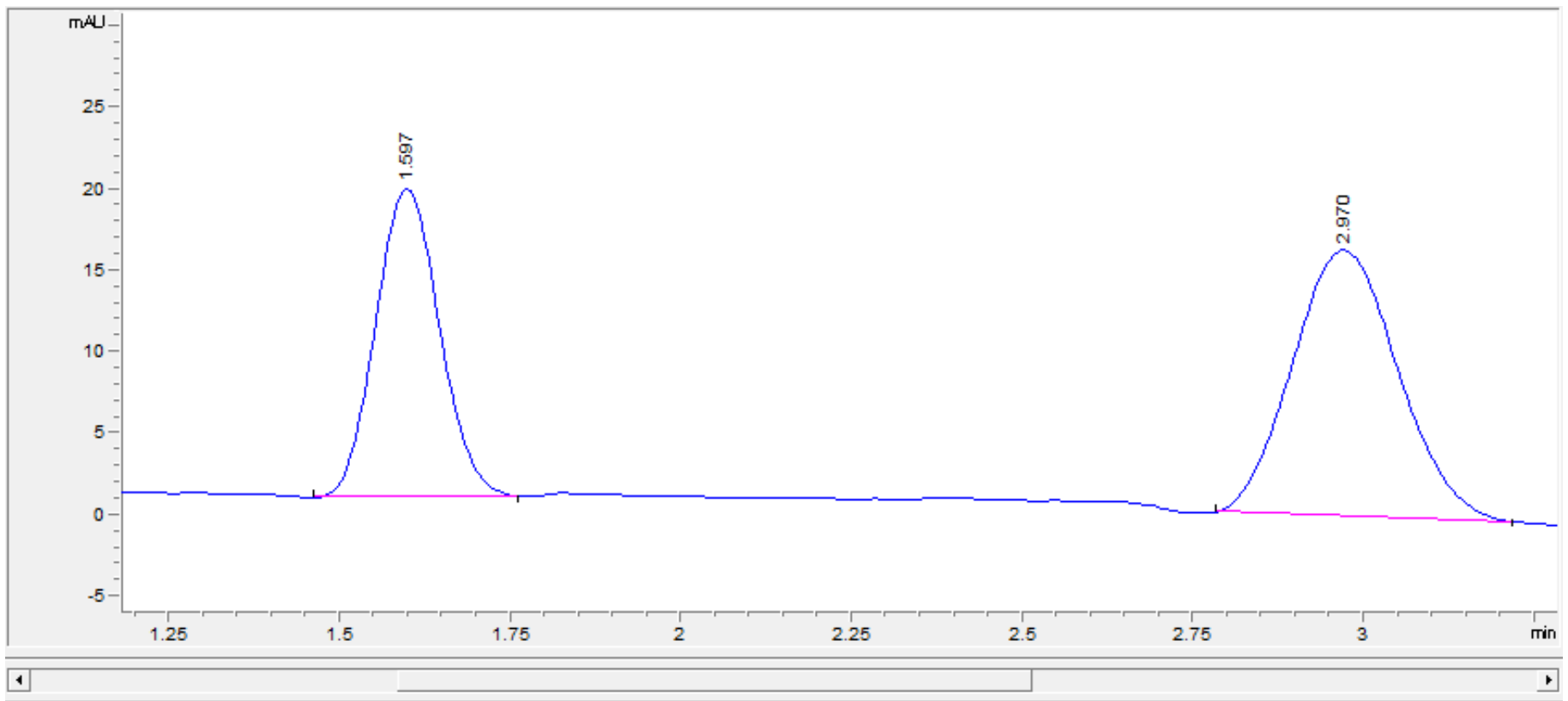

#	Time	Area	Height	Width	Area%	Symmetry
1	1.597	123.1	18.9	0.1018	41.079	0.893
2	2.97	176.5	16.4	0.1799	58.921	0.904

Chiral HPLC chromatogram of **3iii** standard

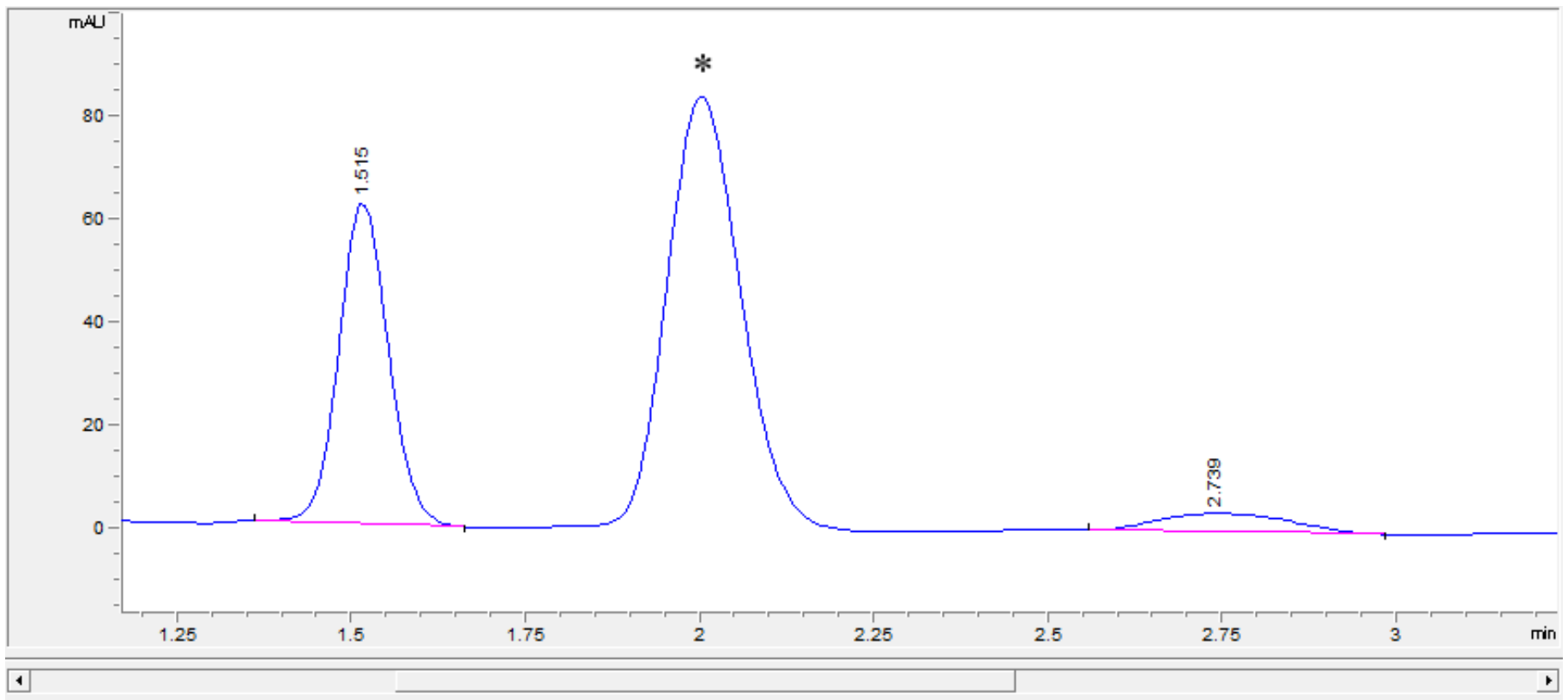

#	Time	Area	Height	Width	Area%	Symmetry
1	1.515	307.3	62.4	0.082	86.647	0.895
2	2.739	47.4	3.6	0.2201	13.353	0.693

Chiral HPLC chromatogram of (*R*)-**3iii** from a biotransformation† using (*S*)-**3**

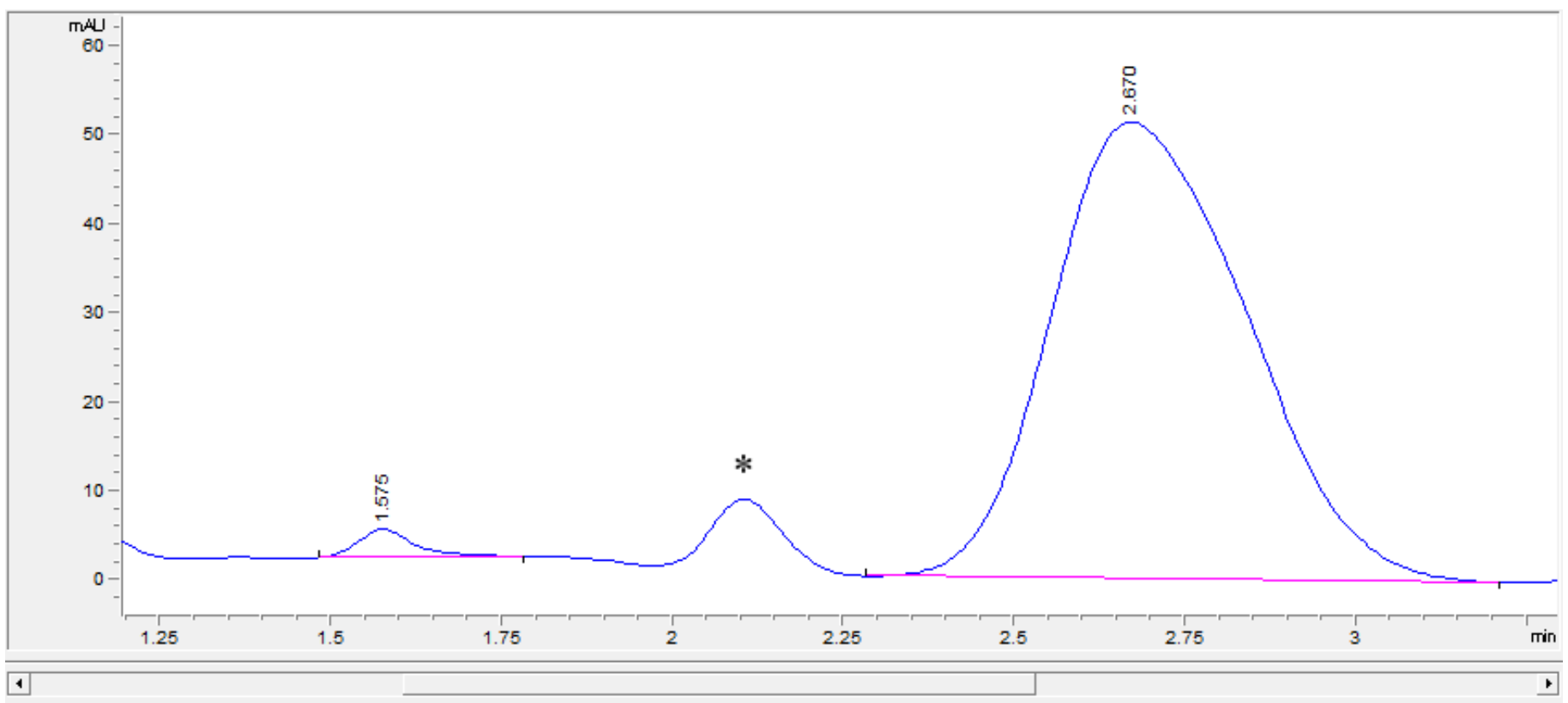

#	Time	Area	Height	Width	Area%	Symmetry
1	1.575	18.5	3.1	0.0986	1.770	0.706
2	2.67	1024.9	51.3	0.3272	98.230	0.679

Chiral HPLC chromatogram of (*S*)-**3iii** from a biotransformation† using (*R*)-**3**

* peaks are unreacted starting material

† NaOH was added after quenching to retain PNP in the aqueous phase, avoiding overlap with **3iii** at 2.9 min

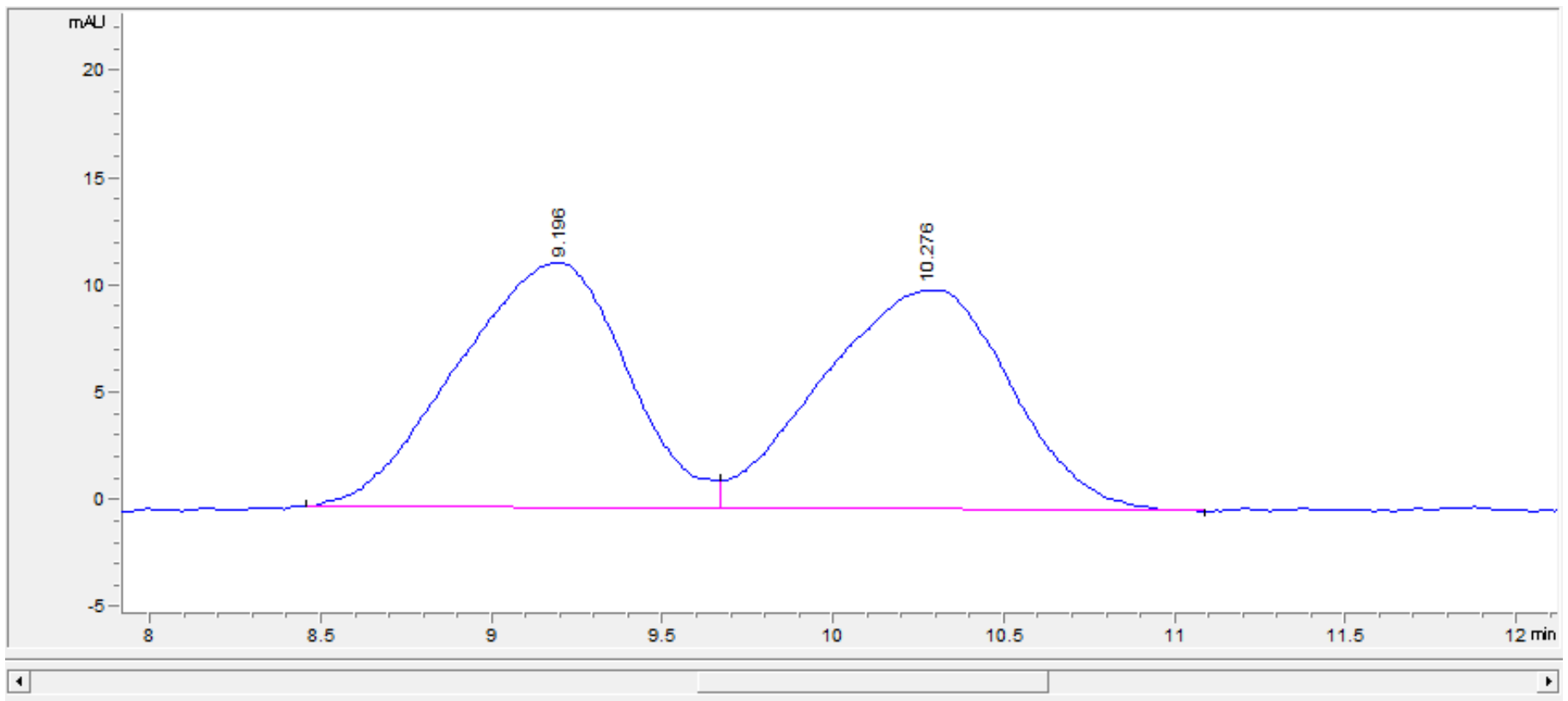

#	Time	Area	Height	Width	Area%	Symmetry
1	9.196	395.8	11.4	0.4549	50.223	1.461
2	10.276	392.3	10.3	0.4584	49.777	1.243

Chiral HPLC chromatogram of **3vii** standard

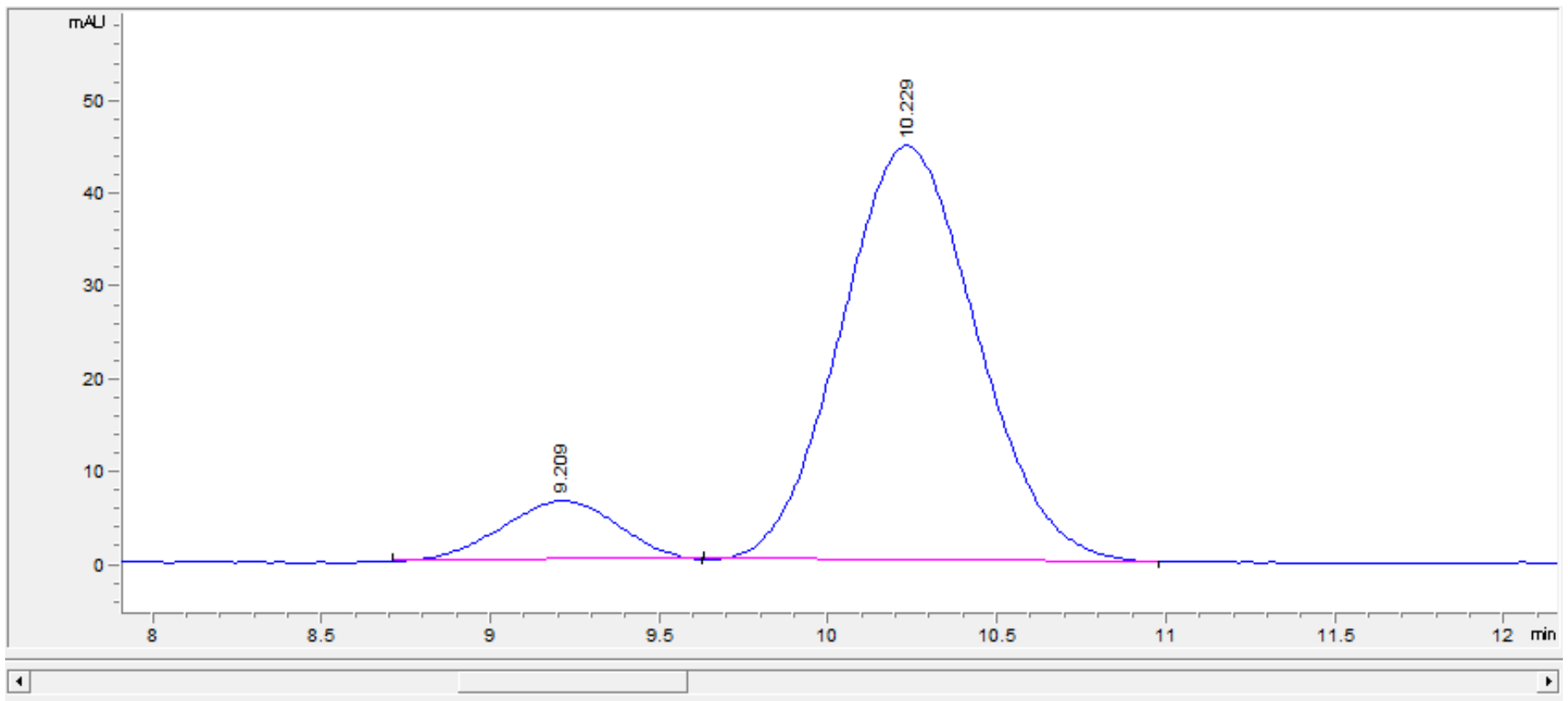

#	Time	Area	Height	Width	Area%	Symmetry
1	9.209	153.8	6.5	0.2937	10.961	1.08
2	10.229	1249.3	44.7	0.4322	89.039	0.905

Chiral HPLC chromatogram of (*R*)-**3vii** from a biotransformation using (*R*)-**3**

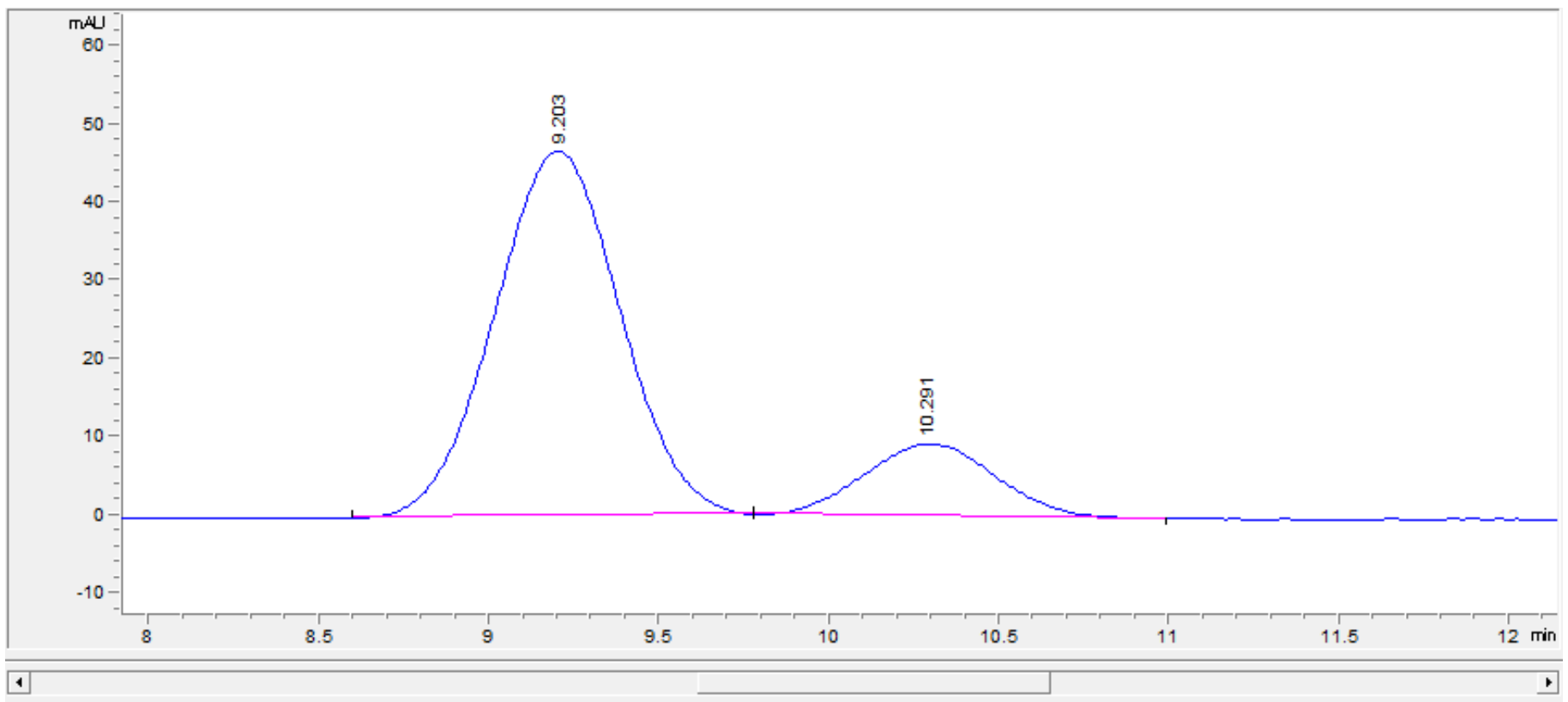

#	Time	Area	Height	Width	Area%	Symmetry
1	9.203	1201.6	46.7	0.3985	82.777	1.01
2	10.291	250	9.3	0.3532	17.223	0.927

Chiral HPLC chromatogram of (*S*)-**3vii** from a biotransformation using (*S*)-**3**

Supplementary References

1. Hutton, A. E. *et al.* A non-canonical nucleophile unlocks a new mechanistic pathway in a designed enzyme. *Nat. Commun.* **15**, 1956 (2024).
2. Gharpure, S. J., Niranjana, P. & Porwal, S. K. Cascade Radical Cyclization to Vinylogous Carbonates/Carbamates for the Synthesis of Oxa- and Aza-Angular Triquinanes: Diastereoselectivity Depends on the Ring Size of Radical Precursor. *Synthesis* **50**, 2954-2967 (2018).
3. Nicponski, D. R. 4-(Dimethylamino)pyridine as a catalyst for the lactonization of 4-hydroxy-2-methylenebutanoate esters. *Tetrahedron Lett.* **55**, 2075-2077 (2014).
4. Ren, H.-X. *et al.* Substituted (*E*)-2-Methylene-3,4-cyclohexenones through Direct and Convenient Synthesis from Cyclohexenone-MBH Alcohol in the Presence of DMAP. *Eur. J. Org. Chem.* **2019**, 715-719 (2019).
5. Bai, C. *et al.* O^2-(6-Oxocyclohex-1-en-1-yl)methyl diazen-1-ium-1,2-diolates: a new class of nitric oxide donors activatable by GSH/GSTπ with both anti-proliferative and anti-metastatic activities against melanoma. *ChemComm* **53**, 5059-5062 (2017).
6. Rezgui, F. & El Gaïed, M. M. Réaction régiospécifique des carbanions stabilisés avec la 2-(acétoxyméthyl)cyclohex-2-én-1-one: Synthèse de diénones bicycliques. *Tetrahedron* **53**, 15711-15716 (1997).
7. White, D. H., Noble, A., Booker-Milburn, K. I. & Aggarwal, V. K. Diastereoselective Photoredox-Catalyzed [3 + 2] Cycloadditions of N-Sulfonyl Cyclopropylamines with Electron-Deficient Olefins. *Org. Lett.* **23**, 3038-3042 (2021).
8. Crawshaw, R. *et al.* Engineering an efficient and enantioselective enzyme for the Morita–Baylis–Hillman reaction. *Nat. Chem.* **14**, 313-320 (2022).
9. Baioui, N., Elleuch, H. & Rezgui, F. Transition-metal-free nucleophilic allylic substitutions of Morita–Baylis–Hillman bromides with aliphatic and aromatic amines. *Synth. Commun.* **46**, 1796-1802 (2016).
